# Effect on *Arabica* Coffee Flavor Quality of Enhanced Fermentation With *Pichia membranifaciens* Through Change Microbial Communities and Chemical Compounds

**DOI:** 10.1002/fsn3.70512

**Published:** 2025-06-28

**Authors:** Xiaojing Shen, Qi Wang, Jia Zheng, Xingyu Li, Song Li, Yanhua Yin, Mengli Shang, Kunyi Liu, Wenjuan Yuan, Jilai Zhang

**Affiliations:** ^1^ College of Science & College of Food Science and Technology & College of Resources and Environment Yunnan Agricultural University Kunming China; ^2^ School of Wuliangye Technology and Food Engineering Yibin Vocational and Technical College Yibin China; ^3^ Wuliangye Yibin Co. Ltd. Yibin China

**Keywords:** chemical compound, *Coffea arabica*, enhanced fermentation, flavor, microbial community, *Pichia membranifaciens*

## Abstract

To stabilize *Arabica* coffee quality by enhanced fermentation with *Pichia membranifaciens*, high‐throughput sequencing, UPLC–MS/MS, HS‐SPME‐GC–MS, and SCA cupping protocol were employed for comprehensive analysis of the coffee fermentation process. Gene sequencing showed that the predominant microorganisms at the genus level were *Weissella*, *Lactococcus*, *Trichococcus*, *Leuconostoc*, and *Massila* for bacteria and *Pichia*, *Hanseniaspora*, *Lachancea*, *Candida*, and *Cystofilobasidium* for fungi. Meanwhile, 122 and 122 differentially changed nonvolatile compounds (VIP > 1, *p* < 0.05, FC > 1.5 or FC < 0.65) from 2275 nonvolatile compounds were found between PE2 versus PB2 and PE3 versus PB3, respectively. Furthermore, 26 differentially changed volatile compounds (VIP > 1, *p* < 0.05, FC > 2.0 or FC < 0.50) were found between PE and PB. Therefore, enhanced fermentation with *P. membranifaciens* inhibited the growth of other microorganisms and changed the chemical compounds during the fermentation to stabilize flavor quality.

## Graphical abstract

*Weissella*, *Lactococcus*, *Trichococcus*, *Leuconostoc*, and *Massila* at the bacterial general level and *Pichia*, *Hanseniaspora*, *Lachancea*, *Candida*, and *Cystofilobasidium* at the fungi general level were the predominant microorganisms during the enhanced fermentation of *Arabica* coffee. Meanwhile, 122 differentially changed nonvolatile compounds (VIP > 1, *p* < 0.05, FC > 1.5 or FC < 0.65) were found between PE and PB. Furthermore, 26 differentially changed volatile compounds (VIP > 1, *p* < 0.05, FC > 2.0 or FC < 0.50) were found between PE and PB.

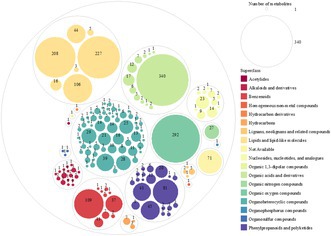

## 1 Introduction

Coffee is one of the most consumed beverages in the world and an important agricultural economic crop in coffee plant regions, such as Brazil, Indonesia, India, Colombia, Ethiopia, Honduras, Peru, Mexico, Guatemala, Nicaragua, China, Vietnam, Costa Rica, Uganda, Papua New Guinea, and other countries around the world (Nawaz et al. 2024; Shen et al. 2022; Silva et al. 2013; Elhalis et al. 2023). In June 2024, world coffee exports amounted to 10.78 million bags. According to USDA (United States Department of Agriculture) statistical data, arabica, robusta, and total coffee production reach 99,855, 76,380, and 176,235 thousand of 60 kg bags, respectively. Furthermore, world coffee production for 2024/25 is forecast to 7.1 million bags. In addition, the ICO Composite Indicator Price (I‐CIP) averaged 236.54 US cents/lb., which increased 4.3% from June 2024 and 48.9% compared with the July 2023 I‐CIP.

The first step of the wet processing is removing the coffee pulp to obtain the green coffee beans, which is crucial for forming coffee flavor. In this process, microorganisms in coffee fermentation produce various enzymes to degrade mucilage and metabolite for coffee precursor substances of coffee flavor (Haile and Kang 2019). However, spontaneous fermentation of coffee leads to unstable coffee flavor quality, such as color difference, and bitterness difference (Wu et al. 2024). With the increase in consumer demand for high‐quality coffee, some coffee processing technology, such as anaerobic germination, fermentation starter optimization, primary processing innovation, and other methods, have been used to improve coffee quality (Wang et al. 2024; Borém et al. 2024; Várady et al. 2022; Aswathi and Murthy 2024). Using specific microorganisms in coffee fermentation is an effective method for controlling coffee flavor quality. For example, coffee using sequential inoculation of *Lactiplantibacillus plantarum* and *Saccharomyces cerevisiae* could produce a strong fruit perception and fermented flavor and form greater volatiles (Rabelo et al. 2024). Coffee inoculated with *Saccharomyces cerevisiae* and *Bacillus amyloliquefaciens* had higher sensory scores (Ferreira et al. 2024). In addition, fermentation with different microorganisms can lead to different sensory characteristics. Yeast (such as *Pichia fermentans* , *P. kudriavzevii*, *Torulaspora delbrueckii*, *Hanseniaspora uvarum, Candida railenensis*, *C. xylopsoci, Wickerhamomyces anomalus*, etc.) and bacteria (such as *Leuconostoc mesenteroides* , *Lactiplantibacillus plantarum*, etc.) starters are common microorganisms in coffee fermentation (Elhalis et al. 2021; Rocha et al. 2023; Cassimiro et al. 2023).

To further control and stabilize the coffee flavor of *C. arabica* , enhanced fermentation with *Pichia membranifaciens* was used in this study. To further understand and obtain information about the enhanced fermentation with *P. membranifaciens*, the changes in microbial communities were analyzed, and nonvolatile compounds (nVCs), volatile compounds (VCs), and coffee sensory were evaluated.

## 2 Materials and Methods

### 2.1 Plant Material and Reagents

The raw materials were mature *Coffea arabica* cherries from Pu‐er City, Yunnan, China collected in January 2024. Methyl alcohol of high‐performance liquid chromatography (HPLC) grade, acetonitrile, and propyl alcohol were purchased from Fisher Co. Ltd. (Shanghai, China). HPLC‐grade *n*‐hexane was sourced from Merck KgaA (Darmstadt, Germany).

### 2.2 Sample Preparation

First, the coffee skin and pulp were removed by hand‐picking and de‐pulping. Subsequently, two fermentation ways were used to remove the thin mucilaginous layer surrounding the coffee seeds. One was spontaneous fermentation under a natural environment, this group was marked as PB. The other was enhanced fermentation with *P. membranifaciens* (5.3 × 10^6^ CFU/mL) based on the preliminary experiments, this group was marked as PE. During the fermentation, each of the three coffee samples in two ways was obtained for throughput sequencing analysis and UPLC–MS/MS analysis at 0 h (PB1, PE1), 24 h (PB2, PE2), and 48 h (PB3, PE3), respectively (Mahingsapun et al. 2022; Elhalis et al. 2021; Pereira et al. 2014). Then, the coffee seeds were washed and dried to obtain green coffee beans. Finally, green coffee beans underwent medium roasting to obtain roasted coffee beans used for HS‐SPME‐GC‐MS and sensory analysis.

### 2.3 High‐Throughput Sequencing Analysis

High‐throughput sequencing analysis of coffee samples during fermentation processing was carried out following DNA extraction and PCR amplification by Majorbio Bio‐Pharm Technology Co. Ltd (Shanghai, China). For bacteria, the hypervariable region V5–V7 of the 16S rRNA gene was amplified with forward primer 799F and reverse primer 1193R. Meanwhile, the ITS1 region used ITS1F and ITS2R primers for fungi (Shen, Wang, et al. 2023, 2024). Moreover, the raw sequencing reads of bacterial 16S rRNA and fungal ITS1 were deposited into the NCBI Sequence Read Archive (SRA) database (Accession Number: PRJNA1088924 and 1089065).

### 2.4 UPLC–MS/MS Analysis

Nonvolatile compounds (n‐VCs) were analyzed using UPLC–MS/MS. 50 mg coffee powder samples were extracted using 80% methanol solution. Then, the extracts were centrifuged at 13,000× *g* for 15 min at 4°C to obtain the supernatant for UPLC–MS/MS analysis by a UHPLC‐Q‐Exactive system from Thermo Fisher Scientific (Bremen, Germany). The chromatographic separation was performed using an HSS T3 C18 column (2.1 × 100 mm, 1.8 μm; Waters Corporation, Milford, MA, USA). The mobile phase consisted of a mixture of (A) 0.1% formic acid in water:acetonitrile (95:5, v/v) and (B) 0.1% formic acid in acetonitrile:isopropanol:water (47.5:47.5:5, v/v). The gradient elution was as follows: 0%–5% B for 0–0.1 min, 5%–25% B for 0.1–2 min, 25%–100% B for 2–9 min, 100% B for 9–13 min, and 100%–0% B for 13–13.1 min, then 0% B for 13.1–16 min to equilibrating the systems. Optimal conditions of the mass spectrum were heater temperature, 400°C; sheath gas flow rate, 40 arb; aux gas flow rate, 10 arb; ion‐spray voltage floating (ISVF), −2800 V in negative mode and 3500 V in positive mode; and normalized collision energy, 20–40–60 V for MS/MS. The detection range covered a mass range of 70–1050 *m*/*z*. Quality control (QC) samples were prepared by combining equal volumes of all samples. The LC–MS was preprocessed using Progenesis QI software (Waters Corporation, USA). Simultaneously, n‐VCs were searched and identified by the HMDB Metlin and Majorbio Database. The response intensity of the sample mass spectrum peaks was normalized using the sum normalization method, and variables with a relative standard deviation (RSD) > 30% of QC samples were removed, followed by log10 calculations (Shen, Wang, et al. 2023).

### 2.5 HS‐SPME‐GC–MS Analysis

HS‐SPME combined with GC–MS (8890‐7000D, Agilent Technologies Inc., Santa Clara, CA, USA) was used to analyze volatile compounds (VCs). 500 mg of roasted coffee bean samples with 20 μL of 10 μg/mL 3‐hexanone as an internal standard were extracted using HS‐SPME. The mixture was equilibrated at 60°C for 5 min. Then, the SPME (120 μm DVB/CWR/PDMS) fiber was preheated at 250°C for 5 min in the Fiber Conditioning Station and was inserted into the headspace vial to extract for 15 min. After extraction, the extracts were analyzed using GC–MS with a DB‐5MS capillary column (30 m × 0.25 mm × 0.25 μm, Agilent J&W Scientific). Helium (≥ 99.999% purity) was used as the carrier gas and maintained at a constant flow rate of 1.2 mL⋅min^−1^. The inlet temperature was set at 250°C in splitless mode. The column temperature was maintained at 40°C for 3.5 min, then programmed to increase at a rate of 10°C⋅min^−1^ to 100°C, followed at a rate of 7°C⋅min^−1^ to 180°C, and finally at a rate of 25°C⋅min^−1^ to 280°C, then maintained at 280°C for 5 min. The following conditions for MS are electron impact (EI) ionization mode at 70 eV. The quadrupole mass detector, ion source, and transfer line temperatures were set at 150°C, 230°C, and 280°C, respectively. The MS was selected ion monitoring (SIM) mode to identify and quantify analysis.

### 2.6 Sensory Analysis

The sensory analysis of all medium‐roasting coffee samples followed the SCA cupping protocol (the Specialty Coffee Association, 2018), which is the only one to define specialty coffee (Ferreira et al. 2023). Ten attributes (fragrance, flavor, aftertaste, acidity, body, balance, uniformity, sweetness, cleanliness, and overall impression) were categorized and scored based on quality by nine certified professionals (five males and four females, aged 18–35 years) with expertise in cupping analysis from Anke Coffee Limited Company (Kunming, China). In brief, uniformity, sweetness, and cleanliness were used to reflect the absence of defects with 10 points. Other attributes were scored based on their quality on a scale of 6–10 points in intervals of 0.25 points. For the SCA, the definition of beverage quality comes from specialty coffees characterized by not having any defect in the beverage, achieving at least 80 points in the rating scale for specialty coffees, and presenting differentiated quality and a high potential for aroma and taste expression (Ferreira et al. 2023; Rocha et al. 2023). Simultaneously, the characteristics of fragrance, aroma, aftertaste, body were detailed descriptions (Shen, Zi, et al. 2023). Meanwhile, the attributes evaluated the flavor characteristics of coffee samples, including flower, sweetness, roasted, nutty, and fruity, ranging from 1 (extremely disliked) to 10 (extremely liked) points (DePaula et al. 2023).

### 2.7 Statistical Analysis

All results from three replicates were presented as the mean value ± standard deviation (SD). Variable importance in projection (VIP) analysis ranked the overall contribution of each variable to the orthogonal partial least squares discriminant analysis (OPLS‐DA) model. Those variables with VIP > 1.0, *p* < 0.05, and fold change (FC) > 1.5 or < 0.65 were classified as differentially changed nonvolatile compounds (DCn‐VCs), whereas fold change (FC) > 2.0 or < 0.5 were classified as differentially changed volatile compounds (DCVCs).

## 3 Results

### 3.1 The Results of High‐Throughput Sequencing Analysis

**FIGURE 1:**
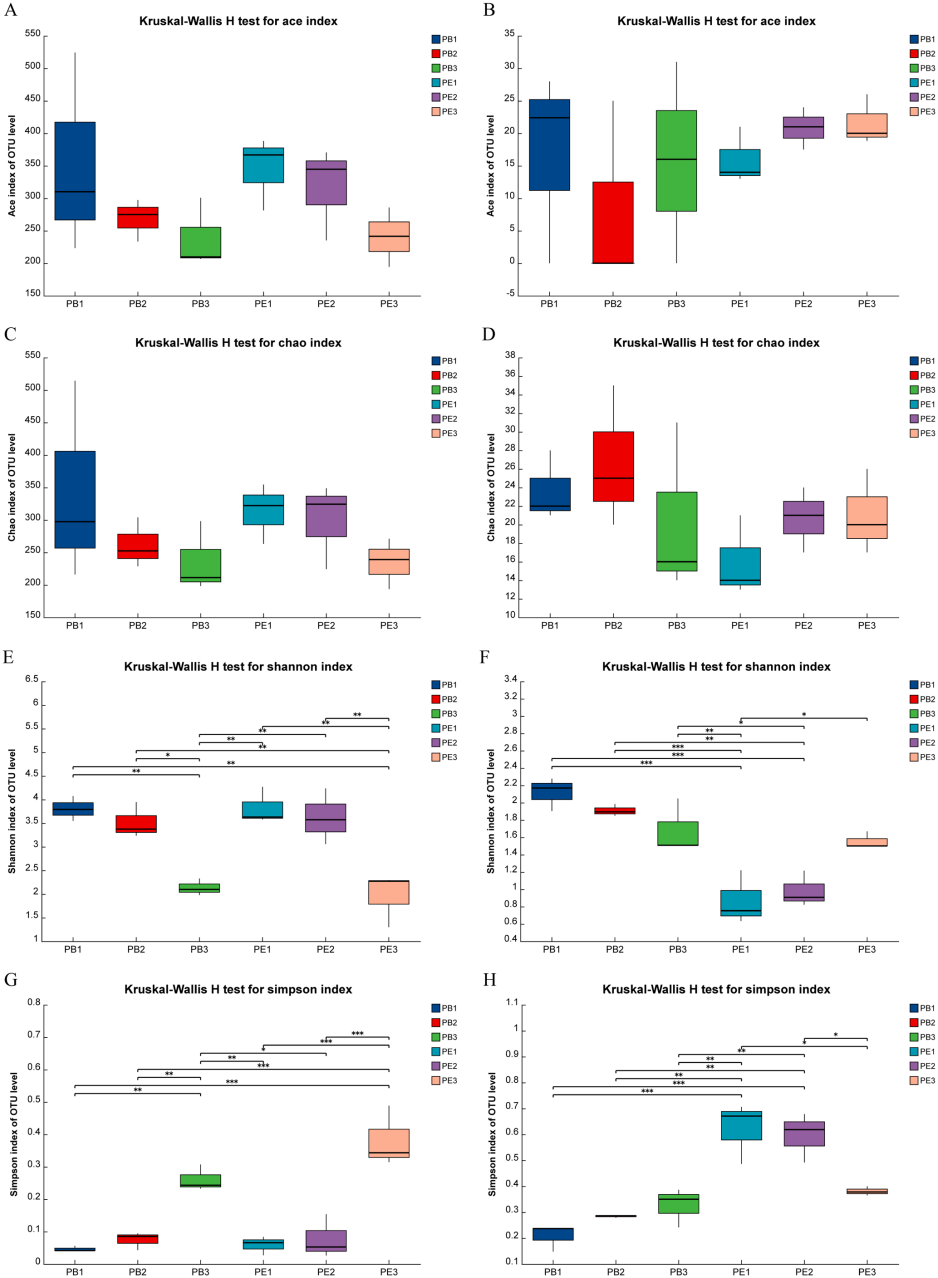
The microbial alpha diversity during coffee fermentation. (A) Ace index in bacteria, (B) Ace index in fungi, (C) Chao index in bacteria, (D) Chao index in fungi, (E) Shannon index in bacteria, (F) Shannon index in fungi, (G) Simpson in bacteria, (H) Simpson in fungi, respectively. * indicated a significant difference with *p* < 0.05, ** indicated an extremely significant with *p* < 0.01, *** indicated an very extremely significant with *p* < 0.001.

1,076,137 bacterial and 1,320,890 fungal sequences were clustered into 1044 bacterial operational taxonomic units (OTUs) and 102 fungi OTUs, respectively. The coverage index for bacteria and fungi in all coffee samples ranged from 99.89% to 100%, which means the sequencing results provided a comprehensive and accurate reflection of the microbial diversity (Wang et al. 2023). The results of the Alpha diversity analysis are shown in Figure 1, which describes and compares microbial biodiversity. Chao and Shannon indices reflect microorganism richness and evenness, respectively (Shen, Yuan, et al. 2024; Huang et al. 2022; Wang et al. 2023). Based on the Chao index, the richness and evenness decreased with the fermentation process for bacteria. For fungi, PB2 in the PB group and PE3 in the PE group showed the maximum richness. Meanwhile, with the fermentation process, the evenness decreased in the PB group while increasing in the PE group.

**FIGURE 2:**
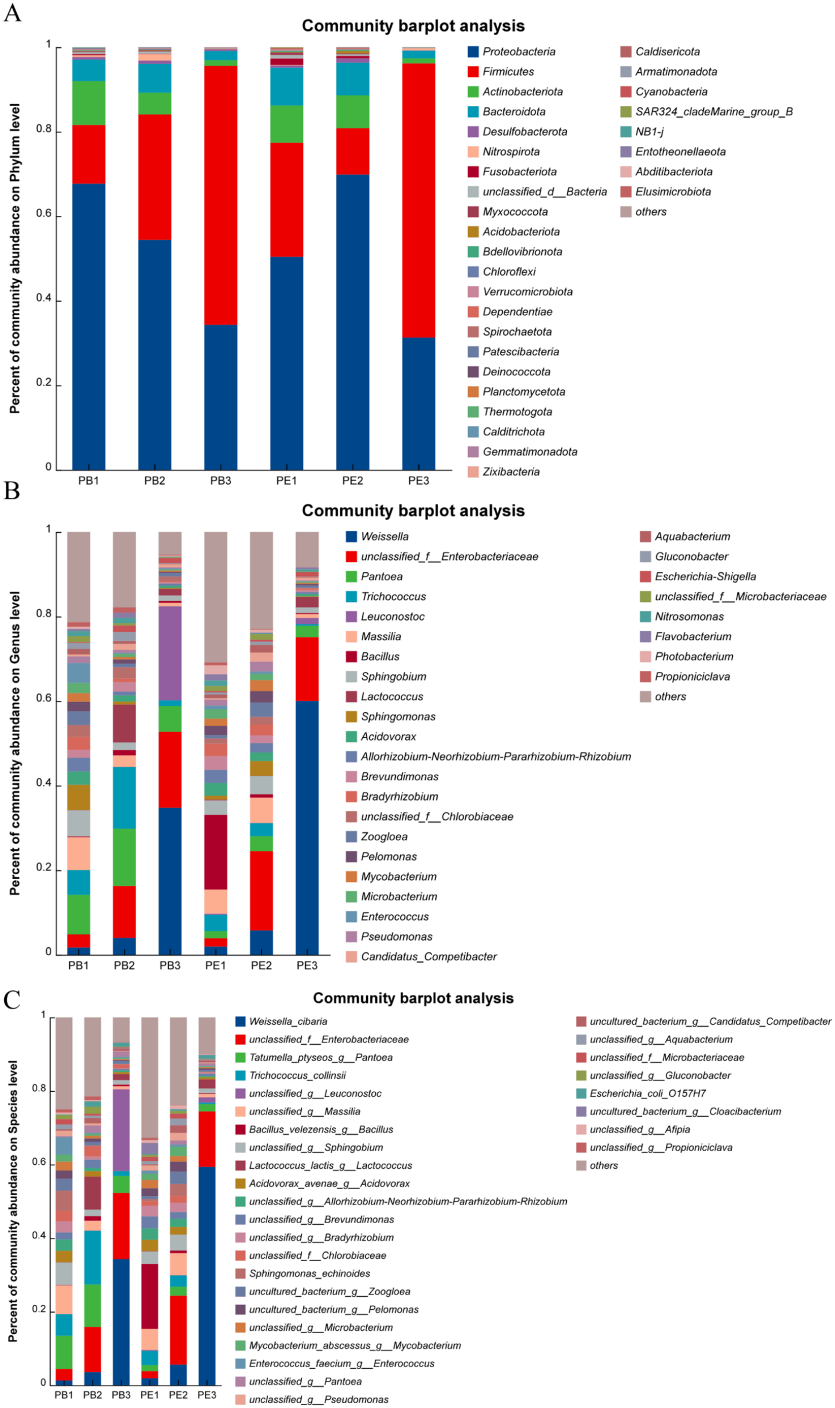
The microbial community of bacteria during coffee fermentation. (A) The percentage of community abundance at the phylum level, (B) The percentage of community abundance at the genus level, (C) the percentage of community abundance at the species level.

For bacteria, 31 phyla during the fermentation of *C. arabica* were identified and shown in Figure 2A, including *Proteobacteria*, *Firmicutes*, *Actinobacteriota*, *Bacteroidota*, *Desulfobacteriota*, *Nitrospirota*, *Fusobacteriota*, *Myxococcota*, *Acidobacteriota*, *Bdellovibrionota*, *Chloroflexi*, etc. Among them, *Proteobacteria* and *Firmicutes* were the predominant phyla in coffee fermentation, followed by *Actinobacteriota*, *Bacteroidota*. In the PB group, the dominant phyla *Proteobacteria* (comprising 67.70% of the community abundance at the phyla level in PB1) decreased to 34.31% at the end of fermentation. In contrast, *Firmicutes* was increased from 13.89% to 61.25%. In the PE group, *Proteobacteria* first increased from 50.40% in PE1 to 69.86% in PE2, then decreased to 31.28% at the end of coffee fermentation. *Firmicutes* showed an opposite change, which decreased from 26.99% (PE1) to 10.95% (PE2), then reached the maximum value of 64.87% (PE3). 31 genus also were identified, including *Weissella*, *Pantoea*, *Trichococcus*, *Leuconostoc*, *Massilia*, *Bacillus*, *Sphingobium*, *Lactococcus*, *Sphingomonas*, *Acidovorax*, *Brevundimonas*, *Bradyrhizobium*, etc., which is shown in Figure 2B. According to the percent of community abundance, *Weissella* increased in the fermentation process with the increasing fermentation duration, *Massil*. Furthermore, the percentage of community abundance at the species level has also been shown in Figure 2C. *Weissella cibaria* , *Trichococcus colinsii*, *Bailus_veleensis_g_bacillus*, *Tatumella_ptyseos_g_pantoea*, and other species were identified. Among them, *Weissella cibaria* increased and reached the maximum values at the end of fermentation.

**FIGURE 3:**
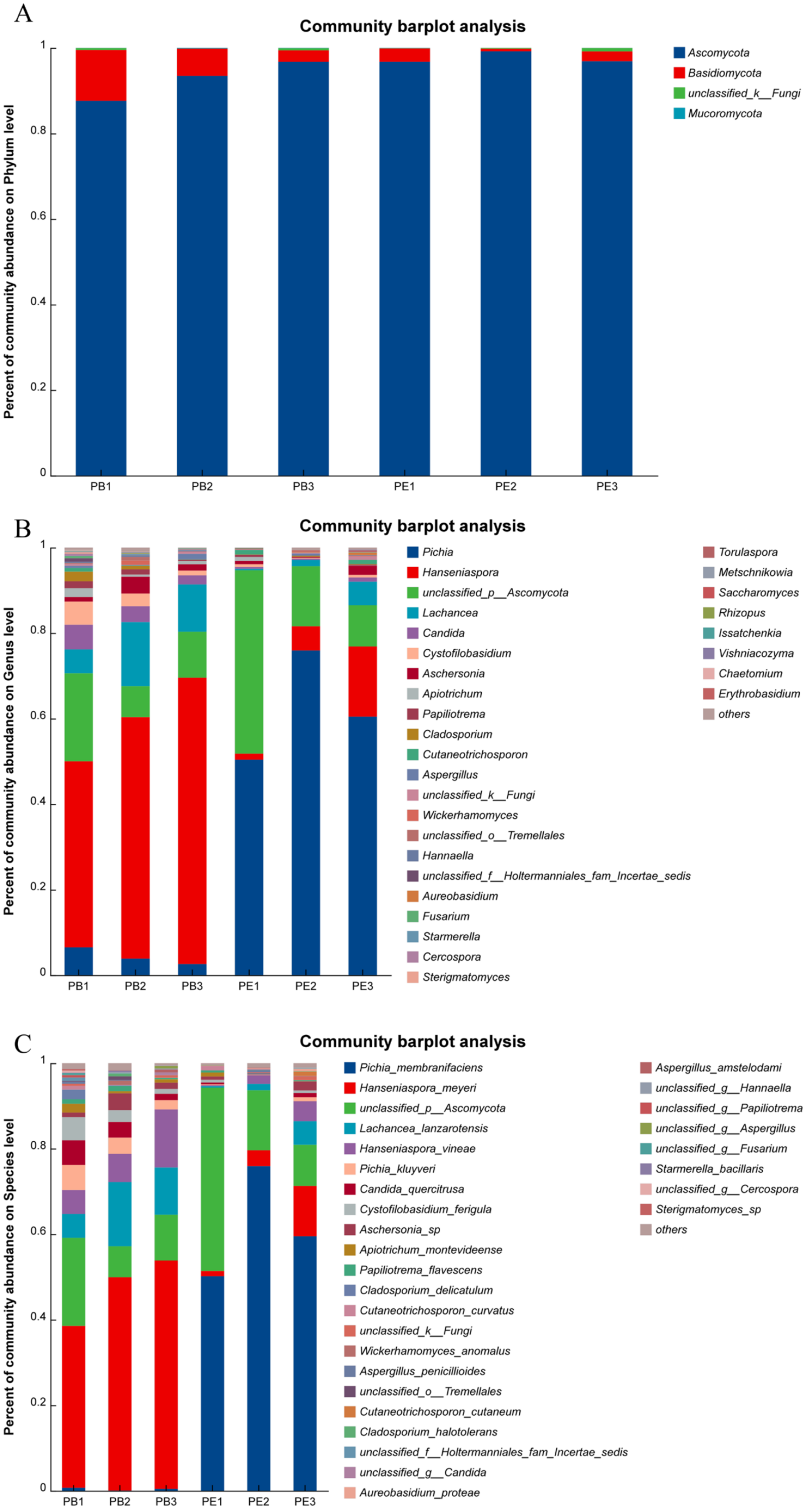
The microbial community of fungi during coffee fermentation. (A) The percentage of community abundance at the phylum level, (B) The percentage of community abundance at the genus level, (C) The percentage of community abundance at the species level.

For fungi, four phyla during the fermentation of *C. arabica* were identified and shown in Figure 3A, including *Ascomycota*, *Basidiomycota*, *Mucoromycota*, and *unclassified_k_ fungi*. *Ascomycota* was the dominant phyla in every coffee sample increasing from 87.61% to 96.74% in PB and from 96.76% to 99.20% in PE. *Basidiomycota* decreased from 11.89% to 2.67% in PB and 3.12% to 0.60% in PE. Thirty‐one genu also were identified including *Pichia*, *Hanseniaspora*, *Lachancea*, *Candida*, *Cystofilobasidium*, *Aschersonia*, *Apiotrichum*, *Papiliotrema*, *Cladosporium*, etc., which was shown in Figure 3B. *Pichia* was the predominant genu in PE, which occupied 50.39% of the community abundance at the phyla level in PE1 and reached the maximum value of 75.92% in PE2. Compared with PE, *Hanseniaspora* was predominant from 43.88% to 66.98% in PB. Furthermore, the percent of the community of abundance on the species level is shown in Figure 3C. In PB, the predominant species in PB was *Hanseniaspora meyeri*, which increased from 37.86% (PB1) to 53.40% (PB3). *Unclassified_p_ascomycota* (7.24%–20.59%), *Lachancea lanzarotensis* (5.57%–15.00%), *Hanseniaspora vineae* (5.63%–13.58%), *Pichia kluyveri* (2.17%–5.84%), *Candida quercitrusa* (1.47%–5.76%), and *Cystofilobasididum ferigula* (1.13%–5.43%) were identified. However, *Pichia membranifaciens* was the predominant species in PE, which occupied 50.18% in PE1 and reached the maximum value of 75.90% in PE2, then decreased to 59.52% in PE3. Moreover, the maximum value of *H. meyeri* was 11.76% in PE3. Then *Unclassified_p_ascomycota ranged from* 9.63% to 42.88%.

### 3.2 The Results of UHPLC–MS/MS Analysis on Nonvolatile Compounds (n‐VCs)

**FIGURE 4:**
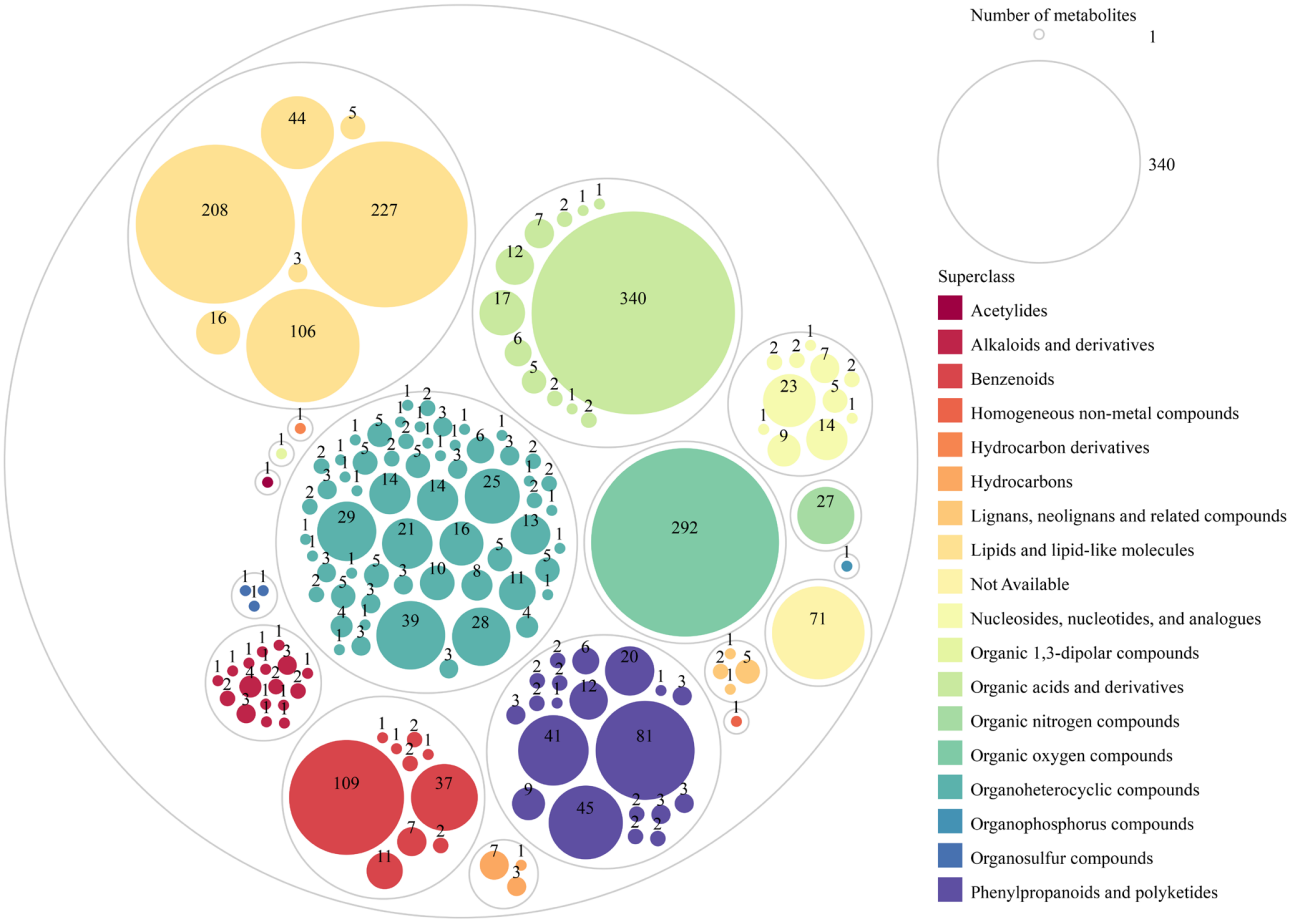
Super‐classes of nonvolatile compounds during Arabica coffee enhanced fermentation with *Pichia membranifaciens*. The different colors represented different superclasses of chemical compounds, and the number was the number of the class in every superclass.

In total, 2275 nonvolatile compounds (n‐VCs) belonging to 18 super‐classes were detected in PB and PE groups during the processing of *C. arabica* with enhanced fermentation with *P. membranifaciens*, as shown in Figure 4. These 18 superclasses included lipids and lipid‐like molecules (609 n‐VCs); organic acids and derivatives (396 n‐VCs); organoheterocyclic compounds (343 n‐VCs); organic oxygen compounds (292 n‐VCs); phenylpropanoids and polyketides (242 n‐VCs); benzenoids (173 n‐VCs); nucleosides, nucleotides, and analogs (67 n‐VCs); alkaloids and derivatives (27 n‐VCs); organic nitrogen compounds (27 n‐VCs); hydrocarbons (11 n‐VCs); lignans, neolignans, and related compounds (9 n‐VCs); organosulfur compounds (3 n‐VCs); acetylides (1 n‐VCs); homogeneous non‐metal compounds (1 nVCs); hydrocarbon derivatives (1 n‐VCs); organic 1,3‐dipolar compounds (1 n‐VCs); organosulfur compounds (1 n‐VCs); and not available (71 n‐VCs). They were further grouped into 153 classes, which mainly included carboxylic acids and derivatives (340 n‐VCs); organooxygen compounds (292 n‐VCs); fatty acyls (227 n‐VCs); prenol lipids (208 n‐VCs); benzene and substituted derivatives (109 n‐VCs); steroids and steroid derivatives (106 n‐VCs); flavonoids (81 n‐VCs); cinnamic acids and derivatives (45 n‐VCs); glycerophospholipids (44 n‐VCs); coumarins and derivatives (41 n‐VCs); indoles and derivatives (39 n‐VCs); phenols (37 n‐VCs); imidazopyrimidines (29 n‐VCs); pyridines and derivatives (28 n‐VCs); organonitrogen compounds (27 n‐VCs); benzopyrans (25 n‐VCs); purine nucleosides (23 n‐VCs), quinolines, and derivatives (21 n‐VCs); isoflavonoids (20 n‐VCs); and others.

**FIGURE 5:**
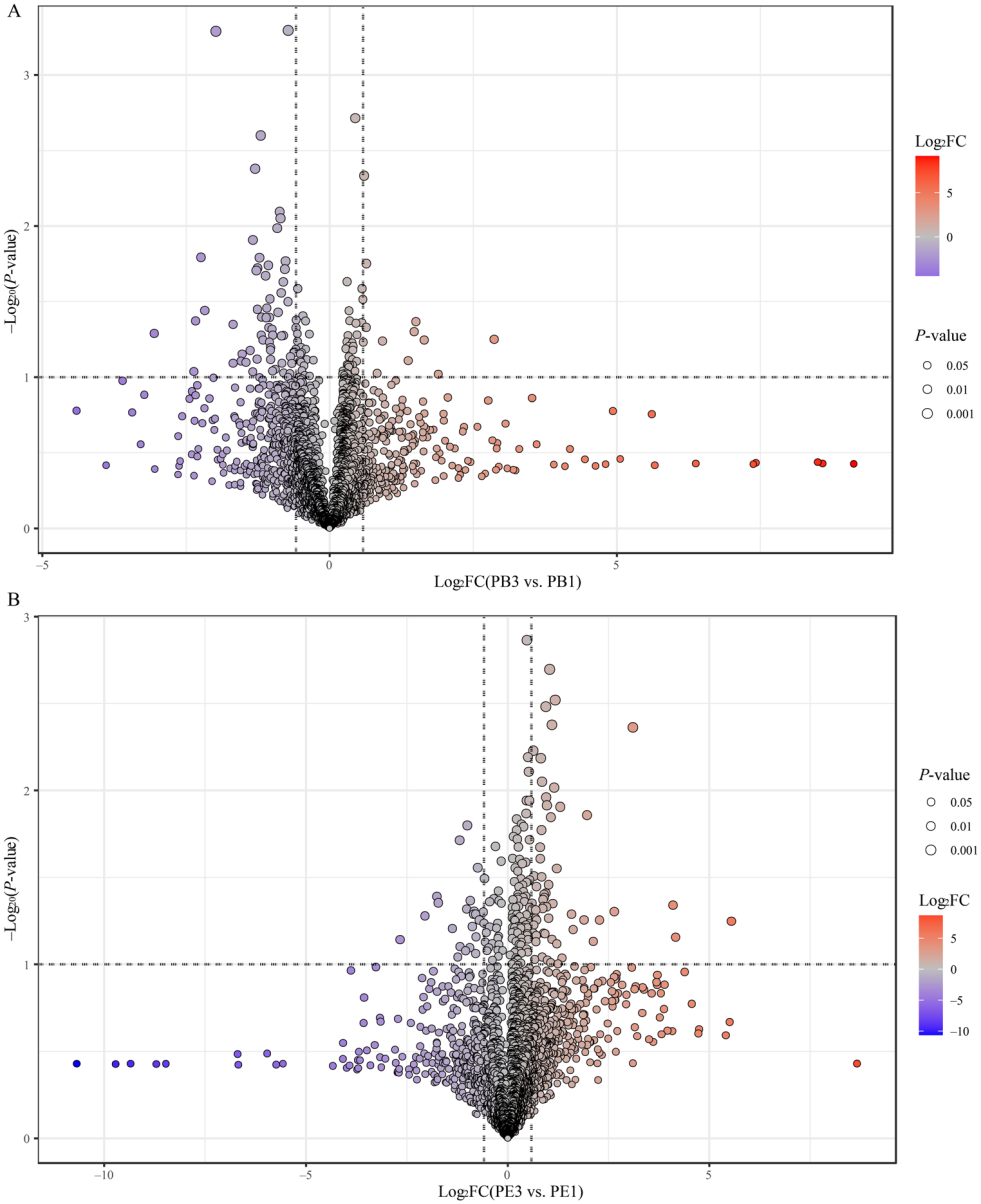
The differentially changed nonvolatile compounds (DCn‐VCs) between PB3 versus PB1 and PE3 versus PE1. A total of 72 DCn‐VCs were found between PB3 and PB1 (A), including 12 upregulated DCn‐VCs and 60 downregulated DCn‐VCs, and 81 DCn‐VCs between PE3 and PE1 (B), including 59 upregulated DCn‐VCs and 22 downregulated DCn‐VCs.

**FIGURE 6:**
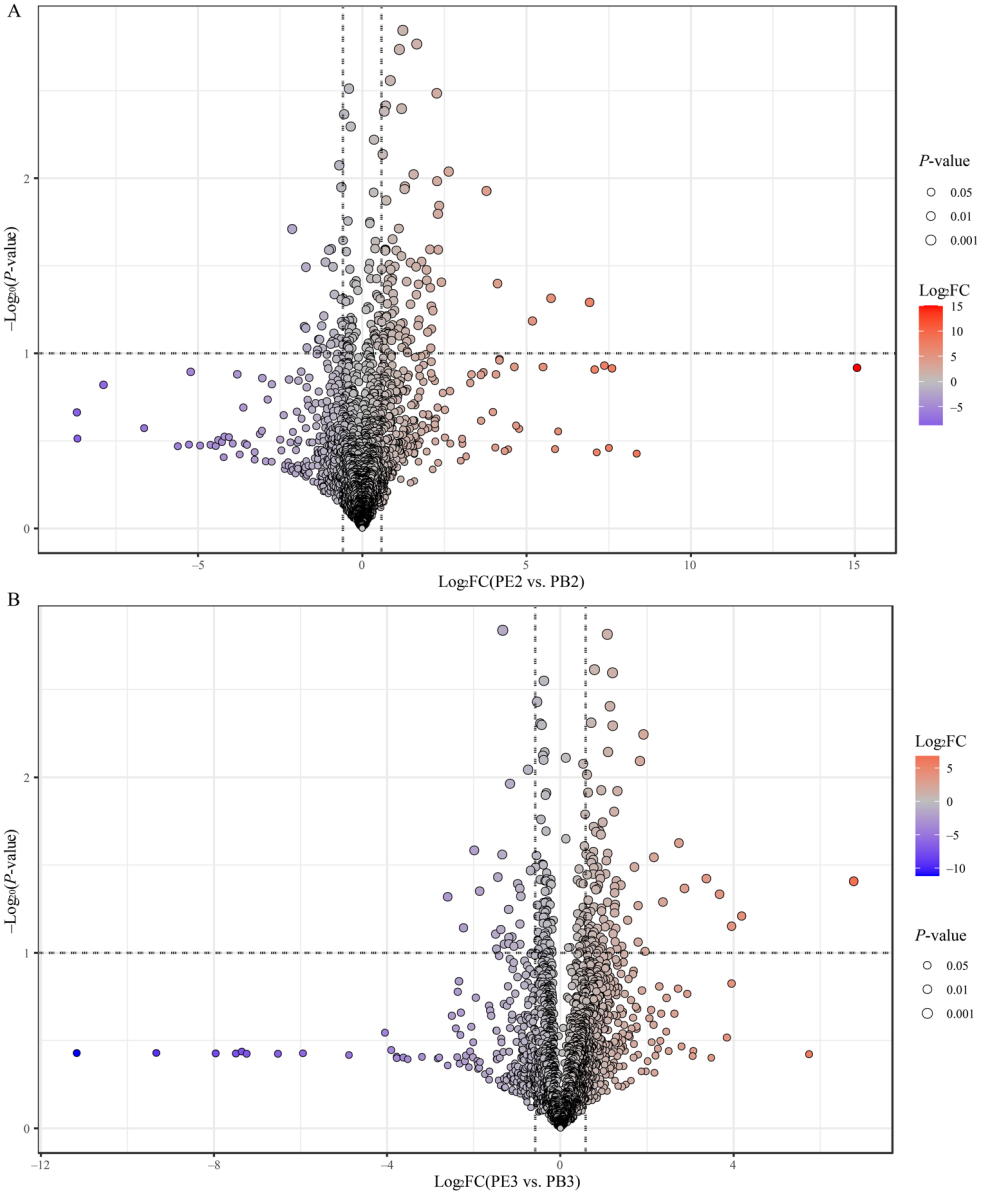
The differentially changed nonvolatile compounds (DCn‐VCs) between PE2 versus PB2 and PE3 versus PB3. A total of 122 DCn‐VCs were found between PE2 and PB2 (A), including 94 upregulated DCn‐VCs and 28 downregulated DCn‐VCs, and 122 DCn‐VCs between PE3 and PB3 (B), including 98 upregulated DCn‐VCs and 24 downregulated DCn‐VCs.

To gain further insights on the change during the processing of *C. arabica* with enhanced fermentation‐*P. membranifaciens*, the differentially changed nonvolatile compounds (DCn‐VCs) with variable importance in projection (VIP) > 1.0, *p* < 0.05, and FC > 1.5 or VIP > 1.0, *p* < 0.05, FC < 0.67, between different groups in PB and PE were assessed and identified, as shown in Figures 5 and 6.

First, 72 DCn‐VCs were detected in the PB3 versus PB1 comparison (Figure 5A). These included 12 upregulated DCn‐VCs and 60 downregulated DCn‐VCs. The upregulated DCn‐VCs included lipids and lipid‐like molecules (five DCn‐VCs: PGP(22:6(4Z,7Z,10Z,13Z,16Z,19Z)/18:2(9Z,12Z)), hexadeca‐7,10,13‐trienoic acid, trihydroxystearic acid, 5‐hexyl‐2‐furanoctanoic acid, and 2‐hydroxystearic acid), organic acids and derivatives (two DCn‐VCs: leucylproline and succinic acid), organoheterocyclic compounds (two DCn‐VCs: N‐methylserotonin, and cinnavalininate), organic oxygen compounds (one DCn‐VC: glucaric acid), benzenoids (one DCn‐VC: succinyladenosine), and nucleosides, nucleotides, and analogs (one DCn‐VC: succinyladenosine). Among them, three DCn‐VCs (N‐methylserotonin, and PGP(22:6(4Z,7Z,10Z,13Z,16Z,19Z)/18:2(9Z,12Z)), and hexadeca‐7,10,13‐trienoic acid) were the most up‐regulated with an FC over 3.0. Meanwhile, organoheterocyclic compounds (19 DCn‐VCs, e.g., 3‐alpha‐hydroxyoreadone, glucosyringic acid, 3‐methylene‐indolenine, indoleacrylic acid, etc.), organic acids and derivatives (nine DCn‐VCs, e.g., dihydrocaffeic acid 3‐sulfate, guanidinosuccinic acid, L‐arogenate, pirimiphos‐methyl, etc.), organic oxygen compounds (nine DCn‐VCs, e.g., tazolol, centpropazine, formyl‐5‐hydroxykynurenamine, damascenone, etc.), lipids and lipid‐like molecules (eight DCn‐VCs, e.g., glycerol 1,2‐dimethacrylate, pisumoside B, 5‐acetamidovalerate, acuminoside, etc.), phenylpropanoids and polyketides (five DCn‐VCs, e.g., 4‐acetylzearalenone, alpha‐methyl‐m‐tyrosine, leucopelargonidin, palustrine, etc.), benzenoids (four DCn‐VCs, e.g., 2‐methylbenzoic acid, tyramine, dimethyl 3,3′‐(6‐methoxy‐6‐oxohex‐1‐ene‐1,1‐diyl)bis(5‐cyano‐6‐methoxybenzoate), acetaminophen, and franguloside), nucleosides, nucleotides, and analogs (one DCn‐VC: (+−)‐carbovir), alkaloids and derivatives (one DCn‐VC: cephaeline), lignans, neolignans, and related compounds (one DCn‐VC: argenteane), and not available (three DCn‐VCs: PA(2:0/PGD2), PA(a‐13:0/22:6(5Z,7Z,10Z,13Z,16Z,19Z)‐OH(4)), and DG(20:5(7Z,9Z,11E,13E,17Z)‐3OH(5,6,15)/2:0/0:0)) were the downregulated DCn‐VCs. Notably, three compounds (e.g., salicin, palustrine, and N‐oleoyl asparagine) were the most downregulated DCn‐VCs, with an FC lower than 0.2.

Similarly, 81 DCn‐VCs were detected (Figure 5B) in the PE3 versus PE1 comparison. These DCn‐VCs included 59 upregulated DCn‐VCs and 22 downregulated DCn‐VCs. The upregulated DCn‐VCs included lipids and lipid‐like molecules (19 DCn‐VCs, e.g., ricinoleic acid, androsterone, isolithocholic acid, acteoside, etc.), organic acids and derivatives (14 DCn‐VCs, e.g., alanylproline, histidylarginine, lupinic acid, 4‐chloro‐l‐phenylalanine, etc.), organic oxygen compounds (seven DCn‐VC, e.g., nicotinamide riboside, 6′‐sialyllactosamine, glucaric acid, 2,4‐dihydroxy‐7,8‐dimethoxy‐2H‐1,4‐benzoxazin‐3(4H)‐one 2‐glucoside, etc.), organoheterocylic compounds (six DCn‐VCs, e.g., bisbynin, andrographolide, fistulosin, (+/−)‐tryptophan, etc.), nucleosides, nucleotides, and analogs (five DCn‐VCs, e.g., succinyladenosine, dephospho‐CoA, adenosine 5′‐monophosphate, 3′,5′‐cyclic GMP, etc.), alkaloids and derivatives (three DCn‐VCs: pilocarpine, ergosine, and lupinic acid), benzenoids (three DCn‐VCs: 4‐heptyloxyphenol, 4‐heptylphenol, and 4‐heptylphenol), phenylpropanoids and polyketides (one DCn‐VC: hapten), and not available (one DCn‐VC: DG(20:5(7Z,9Z,11E,13E,17Z)‐3OH(5,6,15)/2:0/0:0)). Among them, 10 DCn‐VCs (e.g., hydroxyprolyl‐Arginine, alanylproline, pilocarpine, d‐mannose 6‐phosphate, nicotinamide riboside, ethanol and folate, licorice glycoside A, 6′‐sialyllactosamine, bisbynin, beta‐alanyl‐l‐lysine) were the most upregulated with an FC over 3.0. Meanwhile, organic acids and derivatives (five DCn‐VCs, e.g., 2‐isopropyl‐3‐oxosuccinate, l‐aspartic acid, (S)‐isowillardiine, (−)‐2‐difluoromethylornithine, etc.), organic oxygen compounds (five DCn‐VCs, e.g., rutinose, 5‐*p*‐coumaroylquinic acid, licoagroside A, pyro‐l‐glutaminyl‐l‐glutamine, etc.), organoheterocyclic compounds (five DCn‐VCs, e.g., 6‐deoxypenciclovir, ricinine, citreoviridin, 8‐deoxylactucin, etc.), lipids and lipid‐like molecules (four DCn‐VCs: PE(16:0/0:0), (Z)‐5‐((2R,3S,4S,6R)‐4,6‐dihydroxy‐2‐((S,E)‐3‐hydroxyoct‐1‐enyl)tetrahydro‐2H‐pyran‐3‐yl)pent‐3‐enoic acid, etiocholanolone glucuronide, momordin B), phenylpropanoids and polyketides (one DCn‐VC: 3‐(4‐hydroxyphenyl)lactate), benzenoids (one DCn‐VC: phenylpyruvic acid), and not available (one DCn‐VC: PA(2:0/PGD2)) and were the downregulated DCn‐VCs. Notably, taraxacoside was the most downregulated DCn‐VC, with an FC lower than 0.2.

The DCn‐VCs also were analyzed in PB and PE. First, 122 DCn‐VCs were detected (Figure 6A) in the PE2 versus PB2 comparison. These included 94 upregulated DCn‐VCs and 28 downregulated DCn‐VCs. The upregulated DCn‐VCs included lipids and lipid‐like molecules (31 DCn‐VCs, e.g., mometasone, cinncassiol C1 19‐glucoside, 2‐ethyl‐2‐hydroxybutyric acid, azelaic acid, etc.), organoheterocyclic compounds (13 DCn‐VCs, e.g., indolelactic Acid, 1‐methylguanine, pranidipine, (+/−)‐pelletierine, etc.), organic acids and derivatives (12 DCn‐VCs, e.g., hydroxyprolyl‐arginine, astin I, pretyrosine, N‐malonyltryptophan, etc.), phenylpropanoids and polyketides (10 DCn‐VCs, e.g., 4‐hydroxytamoxifen‐N‐glucuronide, 3‐phenyllactic acid, mahaleboside, demethylsuberosin, etc.), organic oxygen compounds (nine DCn‐VCs, e.g., 2,4‐dihydroxy‐7,8‐dimethoxy‐2H‐1,4‐benzoxazin‐3(4H)‐one 2‐glucoside, semilepidinoside A, N‐acetyllactosamine, gluconolactone, etc.), benzenoids (six DCn‐VCs, e.g., gingerol, laflunimus, enol‐phenylpyruvate, 5‐hydroxy‐2‐[2‐methyl‐3‐(trifluoromethyl)anilino]pyridine‐3‐carboxylic acid, etc.), alkaloids and derivatives (three DCn‐VCs: pilocarpine, ethylmorphine, and rescinnamine), nucleosides, nucleotides, and analogs (two DCn‐VCs: 1‐(3‐Fluoro‐4‐hydroxy‐5‐mercaptomethyltetrahydrofuran‐2‐yl)‐5‐methylpyrimidine‐2,4(1H,3H)‐dione and O2′,o3′,o5′‐Tri‐acetyl‐n6‐(3‐hydroxyphenyl)adenosine), and not available (eight DCn‐VOCs: DG(i‐14:0/PGJ2/0:0), Indole carboxylic acid sulfate, PE(DiMe(11,3)/LTE4), PI(6 keto‐PGF1alpha/20:2(11Z,14Z)), etc.). Among them, 8 DCn‐VCs (e.g., gingerol, hydroxyprolyl‐arginine, 13(S)‐HpOTrE, pilocarpine, mometasone, laflunimus, 12S‐HHT, and (2′E,4′Z,7′Z,8E)‐Colnelenic acid) were the most upregulated with an FC over 5.0. Meanwhile, organic acids and derivatives (10 DCn‐VCs, e.g., histidinyl‐leucine, N‐jasmonoylisoleucine, melagatran, 5‐aminovaleric acid, etc.), lipids and lipid‐like molecules (eight DCn‐VCs, e.g., etiocholanolone glucuronide, ginkgolide C, pantoyllactone glucoside, aminodextran, etc.), organoheterocyclic compounds (three DCn‐VCs: dihydrobiopterin, nalidixic acid, and bucolome), benzenoids (three DCn‐VCs: 2‐[1‐[2‐[(4‐carbamimidoylbenzoyl)amino]‐3‐(4‐hydroxyphenyl)propanoyl] piperidin‐4‐yl]oxyacetic acid, norketamine, and 4‐hydroxyphenylacetaldehyde), organic oxygen compounds (two DCn‐VCs: validamycin A, and 1,5‐anhydrosorbitol), and phenylpropanoids and polyketides (two DCn‐VCs: (1′x,2S)‐2‐(1,2‐Dihydroxy‐1‐methylethyl)‐2,3‐dihydro‐7H‐furo[3,2‐g][1]benzopyran‐7‐one 2′‐glucoside and PL) were the downregulated DCn‐VCs. Notably, 3‐hydroxypropyl methacrylate and arginyltryptophan were the most downregulated DCn‐VCs, with an FC lower than 0.3.

In the PE3 versus PB3 comparison, 122 DCn‐VCs were detected (Figure 6B). These included 98 upregulated DCn‐VCs and 24 downregulated DCn‐VCs. The upregulated DCn‐VCs included lipids and lipid‐like molecules (33 DCn‐VCs, e.g., isolithocholic acid, 5beta‐cholanic acid, 20‐carboxy‐leukotriene B4, stachyoside A, etc.), organic acids and derivatives (20 DCn‐VCs, e.g., alanylproline, hydroxyprolyl‐arginine, glabrin C, N‐eicosapentaenoyl serine, etc.), organic oxygen compounds (14 DCn‐VCs, e.g., nicotinamide riboside, d‐arabitol, neamine, cichorioside D, etc.), organoheterocylic compounds (nine DCn‐VCs, e.g., plantamajoside, pradigastat, niacinamide, cowanin, etc.), phenylpropanoids and polyketides (eight DCn‐VCs, e.g., malvidin 3‐alpha‐l‐galactoside, yakuchinone‐A, patamostat, 5‐(6‐hydroxy‐3,7‐dimethyl‐2,7‐octadienyloxy)‐7‐methoxycoumarin, etc.), benzenoids (four DCn‐VCs: pyricarbate, franguloside, 4‐heptyloxyphenol, and tyramine), alkaloids and derivatives (three DCn‐VCs: pilocarpine, ethylmorphine, and berberine), hydrocarbons (one DCn‐VC: aplotaxene), nucleosides, nucleotides, and analogs (one DCn‐VC: CMP), organosulfur compounds (one DCn‐VC: 1,6‐hexanedithiol), lignans, neolignans and related compounds (one DCn‐VC: 8‐hydroxypinoresinol 4‐glucoside), and not available (three DCn‐VCs: PG(PGJ2/a‐13:0), DG(20:4(8Z,11Z,14Z,17Z)‐2OH(5S,6R)/0:0/2:0), and DG(20:5(7Z,9Z,11E,13E,17Z)‐3OH(5,6,15)/0:0/2:0)). Among them, 8 DCn‐VCs (pilocarpine, hydroxyprolyl‐arginine, alanylproline, N‐oleoyl asparagine, nicotinamide riboside, ethanol and folate, N‐eicosapentaenoyl serine, and cyclo(glycylleucylvalylleucylprolylseryl)) were the most upregulated with an FC over 5.0. Meanwhile, lipids and lipid‐like molecules (nine DCn‐VCs, e.g., 2‐(malonylamino)benzoic acid, heliangin, arenobufagin, 3b,8b‐dihydroxy‐6b‐(3‐chloro‐2‐hydroxy‐2‐methylbutanoyloxy)‐7(11)‐eremophilen‐12,8‐olide, etc.), organic acids and derivatives (five DCn‐VCs, e.g., aspartic acid, alanylhydroxyproline, AB‐chminaca, montirelin, etc.), benzenoids (two DCn‐VCs: dodecylbenzenesulfonic acid, and 2‐(Malonylamino)benzoic acid), Organic nitrogen compounds (two DCn‐VCs: (2R,3R)‐2‐aminooctadecane‐1,3‐diol, and choline phosphate), organic oxygen compounds (two DCn‐VCs: arenobufagin, and emulphor), nucleosides, nucleotides, and analogs (one DCn‐VC: O2′,o3′,o5′‐tri‐acetyl‐n6‐(3‐hydroxyphenyl)adenosine), organoheterocyclic compounds (one DCn‐VC: N‐methylserotonin), and not availabe (two DCn‐VCs: MG(22:6(4Z,7Z,11E,13Z,15E,19Z)‐2OH(10S,17)/0:0/0:0), and DG(20:4(6E,8Z,11Z,13E)‐2OH(5S,15S)/2:0/0:0)) were the down‐regulated DCn‐VCs. Notably, MG(22:6(4Z,7Z,11E,13Z,15E,19Z)‐2OH(10S,17)/0:0/0:0), montirelin, N‐methylserotonin, and mimosine were the most downregulated DCn‐VCs, with an FC lower than 0.3.

### 3.3 The Results of HS‐SPME‐GC–MS Analysis on Volatile Compounds (VCs)

Volatile compounds (VCs) are crucially important for determining coffee quality. A total of 917 VCs were detected in PB and PE, as shown in Table S1. They were classified as 16 classes, including terpenoids (194 VCs), heterocyclic compounds (163 VCs), esters (149 VCs), ketones (86 VCs), alcohols (67 VCs), aldehyde (57 VCs), aromatics (57 VCs), hydrocarbons (55 VCs), phenols (26 VCs), acids (21 VCs), sulfur compounds (17 VCs), amine (10 VCs), nitrogen compounds (5 VCs), ethers (4 VCs), halogenated hydrocarbon (1 VC), and others (5 VCs). Among them, 475 VCs showed sweet, fruity, honeydew, rose, creamy, and other aroma characteristics. These odor volatile compounds (OVCs) included esters (101 OVCs), terpenoids (101 OVCs), heterocyclic compounds (80 OVCs), aldehyde (42 OVCs), ketones (41 OVCs), alcohols (36 OVCs), aromatics (20 OVCs), phenols (14 OVCs), sulfur compounds (14 OVCs), acids (11 OVCs), hydrocarbons (8 OVCs), ethers (3 OVCs), amine (2 OVCs), nitrogen compounds (1 OVC), and others (1 OVC), as shown in Table S2. In addition, 184 VCs including heterocyclic compounds (42 VCs), aldehyde (28 VCs), terpenoids (27 VCs), esters (26 VCs), ketones (16 VCs), phenols (12 VCs), aromatics (12 VCs), alcohols (11 VCs), sulfur compounds (5 VCs), acids (3 VCs), hydrocarbons (1 VC), and amine (1 VC) directly contributed to coffee flavor with the relative odor activity value (rOAV) over 1.0, as shown in Table 1. Among them, 3‐cyclohexene‐1‐methanethiol, .alpha.,.alpha.,4‐trimethyl‐; pyrazine, 2‐methoxy‐3‐(1‐methylethyl)‐; pyrazine, 2‐methoxy‐3‐(1‐methylpropyl)‐; pyrazine, 2‐ethyl‐3,5‐dimethyl‐; 3‐octen‐2‐one; 5‐methyl‐(E)‐2‐hepten‐4‐one; ethanone, 1‐(2‐aminophenyl)‐; pentanoic acid, 2‐methyl‐, ethyl ester; 4‐heptenal, (Z)‐; 2‐thiophenemethanethiol were the top 10 coffee flavor compounds with the relative odor activity value (rOAV). Pyrazine, 2‐methoxy‐3‐(1‐methylethyl)‐; and pyrazine, 2‐ethyl‐3,5‐dimethyl‐ mainly contributed to the chocolate, burnt, almond, roasted, nutty coffee flavor. Pentanoic acid, 2‐methyl‐, ethyl ester mainly contributed to fruit flavors such as melon and pineapple. 4‐heptenal, (Z)‐ contributed to an oily, fatty, dairy, milky, creamy flavor of coffee. Ethanone, 1‐(2‐aminophenyl)‐ contributed to grape and sweet coffee flavor. 5‐methyl‐(E)‐2‐hepten‐4‐one related to hazelnut and nutty flavor. These odor compounds formed complicated and attractive coffee flavor.

**TABLE 1:** Volatile compounds (VCs) of coffee samples directly contributed to coffee flavor with the relative odor activity value (rOAV) over 1.0.

No.	Compounds	Class	Odor	rOAV	
				PE	PB
1	(+)‐3‐Carene	Terpenoids	Sweet	8.24 ± 0.35	5.24 ± 3.42
2	(2E,4Z)‐2,4‐Decadienal	Aldehyde	Fried, fatty, geranium, green, waxy	5967.88 ± 1459.72	6202.58 ± 381.23
3	(6Z)‐Nonen‐1‐ol	Alcohol	Fresh, green, melon, waxy, honeydew, cantaloupe, cucumber, clean	115.59 ± 15.77	51.85 ± 59.67
4	(E)‐2‐Decenal	Aldehyde	Waxy, fatty, earthy, green, cilantro, mushroom, aldehydic, fried, chicken, fatty, tallow	5.28 ± 0.70	4.93 ± 0.67
5	(E)‐2‐Heptenal	Aldehyde	Pungent, green, vegetable, fresh, fatty	4.91 ± 1.32	4.84 ± 0.61
6	(E,E)‐2,4‐Undecadienal	Aldehyde	Oily, caramel, spicy, citrus, buttery, baked	5761.33 ± 231.85	4983.13 ± 103.22
7	(Z)‐2‐Heptenal	Aldehyde	—	4.91 ± 1.32	4.84 ± 0.61
8	.alpha.‐Ionone	Terpenoids	Sweet, woody, floral, violet, orris, tropical, fruity	20.85 ± 2.15	15.63 ± 3.02
9	.alpha. –Irone	Terpenoids	Orris, floral, berry, violet, woody, powdery	18.53 ± 3.07	13.99 ± 0.83
10	.alpha.‐Phellandrene 1	Terpenoids	Citrus, herbal, terpene, green, woody, peppery	9.06 ± 0.38	5.76 ± 3.76
11	.alpha.‐Terpineol	Terpenoids	Pine, iris, teil	4.14 ± 0.19	3.64 ± 0.29
12	.beta.‐Ocimene	Terpenoids	Apple, pear, fruity	3.87 ± 2.00	3.86 ± 1.90
13	.beta.‐Phellandrene	Terpenoids	Terpenic, herbal	1.42 ± 0.95	1.39 ± 0.97
14	1,3,6‐Octatriene, 3,7‐dimethyl‐, (Z)—	Terpenoids	Warm, floral, herbal, flowery, sweet	3.87 ± 1.99	3.86 ± 1.90
15	1,3‐Cyclohexadiene‐1‐carboxaldehyde, 2,6,6‐trimethyl—	Terpenoids	Fresh, herbal, phenol, metallic, rosemary, tobacco, spicy	114.98 ± 14.45	111.63 ± 11.10
16	1,4‐Dithiane	Amine	Sulfury, solvent, garlic, onion, pyridine	3.93 ± 0.44	3.06 ± 0.36
17	1‐(4‐methylphenyl)‐Ethanone	Ketone	Green, pea, bell pepper, galbanum	50.83 ± 15.53	38.07 ± 13.03
18	1‐Cyclohexene‐1‐carboxAldehyde, 4‐(1‐methylethenyl)—	Aldehyde	Fresh, green, herbal, grassy, sweet, minty, cumin	6.94 ± 0.98	6.58 ± 0.60
19	1‐Cyclohexene‐1‐carboxAldehyde, 4‐(1‐methylethenyl)‐, (S)—	Aldehyde	Fresh, green, oily, grassy, fatty, minty, cherry	1.8 ± 0.43	1.38 ± 0.35
20	1‐Ethylpropyl acetate	Ester	—	28.94 ± 0.58	26.87 ± 2.40
21	1‐Octen‐3‐one	Ketone	Mushroom	3606.43 ± 962.89	3022.62 ± 639.77
22	2(3H)‐Furanone, 5‐butyldihydro—	Ester	Sweet, coconut, waxy, creamy, tonka, dairy, fatty	3.15 ± 0.78	2.13 ± 0.41
23	2,3‐Dimethyl‐5‐ethylpyrazine	Heterocyclic compound	Burnt, popcorn, roasted, cocoa	24.75 ± 3.07	25.04 ± 3.58
24	2,4,6‐Octatriene, 2,6‐dimethyl—	Terpenoids	Sweet, floral, nut skin, peppery, herbal, tropical	15.99 ± 1.01	12.76 ± 2.50
25	2,4,6‐Octatriene, 2,6‐dimethyl‐, (E,Z)—	Terpenoids	—	15.99 ± 1.01	12.76 ± 2.50
26	2,4‐Octadienal, (E,E)—	Aldehyde	Green, fatty, pear, melon, peel	2.13 ± 0.52	2.03 ± 0.74
27	2,6,6‐Trimethyl‐2‐cyclohexene‐1,4‐dione	Ketone	Musty, woody, sweet, tea, tobacco, leafy	7.48 ± 0.32	6.85 ± 0.61
28	2,6‐Nonadienal, (E,E)—	Aldehyde	Fresh, citrus, green, cucumber, melon	233.86 ± 67.81	215.04 ± 22.05
29	2,6‐Nonadienal, (E,Z)—	Aldehyde	Cucumber, green	11,692.78 ± 3390.70	10,751.98 ± 1102.40
30	2‐Acetyl‐1,4,5,6‐tetrahydropyridine	Heterocyclic compound	Creamy, bread	747.24 ± 495.88	430.58 ± 464.96
31	2‐Acetyl‐3‐methylpyrazine	Heterocyclic compound	Nutty, flesh, roasted hazelnut, toasted grain, corn, chip, vegetable, nut skin, caramel	453.37 ± 66.58	447.22 ± 74.24
32	2‐Acetylthiazole	Heterocyclic compound	Nutty, popcorn, roasted, peanut, hazelnut	88.20 ± 2.17	80.13 ± 6.28
33	2‐Buten‐1‐one, 1‐(2,6,6‐trimethyl‐1,3‐cyclohexadien‐1‐yl)‐, (E)—	Terpenoids	Apple, rose, honey, tobacco, sweet	550.42 ± 7.10	510.68 ± 21.98
34	2‐Cyclohexen‐1‐ol, 2‐methyl‐5‐(1‐methylethenyl)‐, acetate	Ester	Green, minty, spearmint, nasturtium, herbal, rummy, grape, pear, spicy	11.24 ± 1.32	9.26 ± 1.48
35	2‐Cyclopenten‐1‐one, 3‐methyl‐2‐(2‐pentenyl)‐, (Z)—	Ketone	Woody, herbal, floral, spicy, jasmin, celery	677.91 ± 138.79	674.48 ± 38.18
36	2‐Ethoxy‐3‐methylpyrazine	Heterocyclic compound	Hazelnut, roasted, almond, pineapple, earthy	227.86 ± 29.38	208.14 ± 20.15
37	2‐Ethyl‐3‐methoxypyrazine	Heterocyclic compound	Raw, potato, earthy, bell pepper, nutty	99.07 ± 2.23	66.79 ± 43.85
38	2‐FurancarboxAldehyde, 5‐methyl—	Aldehyde	Spice, caramel, maple	257.96 ± 10.83	234.19 ± 8.50
39	2‐Furanmethanethiol, 5‐methyl—	Heterocyclic compound	Sulfury, roasted, coffee	6203.40 ± 1823.43	6755.31 ± 602.84
40	2‐Heptanol	Alcohol	Fruity, moldy, musty, mushroom	4.22 ± 0.26	2.32 ± 1.48
41	2‐Hexanol	Alcohol	Chemical, winey, fruity, fatty, terpenic, cauliflower	1.04 ± 0.12	1.02 ± 0.096
42	2‐Isobutylthiazole	Heterocyclic compound	Green, wasabi, privet, tomato, leafy, earthy, vegetable, metallic	33.05 ± 5.59	32.48 ± 0.37
43	2‐Methoxy‐4‐vinylphenol	Aromatics	Spicy, raisin	43.71 ± 10.37	50.65 ± 6.98
44	2‐Methyl‐1,3‐dithiacyclopentane	Heterocyclic compound	Sulfury, alliaceous, smoky, savory, vegetable	423.54 ± 20.35	394.52 ± 23.13
45	2‐Nonen‐1‐ol, (E)—	Alcohol	Waxy, green, violet, melon	2.71 ± 0.24	2.48 ± 0.12
46	2‐Nonen‐4‐one	Ketone	—	29.55 ± 2.43	27.40 ± 3.52
47	2‐Octanone	Ketone	Earthy, weedy, natural, woody, herbal	9.95 ± 0.55	8.82 ± 1.18
48	2‐Octen‐1‐ol, (E)—	Alcohol	Green, citrus, vegetable, fatty	54.82 ± 1.49	51.82 ± 4.48
49	2‐Thiophenemethanethiol	Heterocyclic compound	Roasted, coffee, fishy	37,707.34 ± 3735.43	29,760.19 ± 3852.92
50	2‐Undecanone	Ketone	Waxy, fruity, creamy, fatty, orris, floral	13.45 ± 1.04	12.39 ± 1.12
51	2‐methoxy‐Phenol	Phenol	Nutty	2250.69 ± 37.20	1996.37 ± 197.31
52	2H‐Pyran, 3,6‐dihydro‐4‐methyl‐2‐(2‐methyl‐1‐propenyl)—	Terpenoids	Green, weedy, cortex, herbal, diphenyl, narcissus, celery	2.05 ± 0.26	1.88 ± 0.061
53	3,5‐Octadien‐2‐one, (E,E)—	Ketone	Fruity, green, grassy	1620.88 ± 342.92	1262.55 ± 61.77
54	3,6‐Nonadien‐1‐ol, (E,Z)—	Alcohol	Fatty, green, cucumber, green pepper, fruity, watermelon	21.18 ± 9.24	16.57 ± 1.50
55	3‐Cyclohexene‐1‐methanethiol, .alpha., .alpha., 4‐trimethyl—	Sulfur compounds	Sulfury, aromatic, grapefruit, naphthyl, resinous, woody	19,181,929.80 ± 6935709.12	14,835,104.66 ± 6,088,008.97
56	3‐Hexen‐1‐ol, acetate, (Z)—	Ester	Fresh, green, sweet, fruity, banana, apple, grassy	4.53 ± 0.28	4.19 ± 0.26
57	3‐Hexenal, (Z)—	Aldehyde	Green, fatty, grassy, weedy, fruity, apple	13.08 ± 0.81	11.52 ± 1.00
58	3‐Mercaptohexanol	Alcohol	Sulfury, fruity, tropical	14,019.68 ± 1437.63	11,675.45 ± 1552.31
59	3‐Mercaptohexyl acetate	Ester	Sulfury, grapefruit, fruity	5289.68 ± 178.94	4362.60 ± 683.69
60	3‐Methoxy‐2,5‐dimethylpyrazine	Heterocyclic compound	Earthy	9907.33 ± 222.56	6678.97 ± 4385.32
61	3‐Octanol	Alcohol	Earthy, mushroom, herbal, melon, citrus, woody, spicy, minty	1.82 ± 0.0098	1.63 ± 0.12
62	3‐Octanone	Ketone	Fresh, herbal, lavender, sweet, mushroom	459.35 ± 25.33	407.27 ± 54.29
63	3‐Octen‐2‐one	Ketone	Earthy, spicy, herbal, sweet, mushroom, hay, blueberry	207,042.13 ± 158,315.04	304,184.56 ± 15,831.12
64	4‐Decenal, (E)—	Aldehyde	Fresh, aldehydic, citrus, orange, mandarin, tangerine, green, fatty	2.42 ± 0.19	2.07 ± 0.22
65	4‐Decenoic acid, methyl ester, Z—	Ester	Fruity, pear, mango, fishy, peach skin, green	236.54 ± 24.33	206.81 ± 3.44
66	4‐Heptenal	Aldehyde	—	305.82 ± 10.61	284.88 ± 8.39
67	4‐Heptenal, (Z)—	Aldehyde	Oily, fatty, green, dairy, milky, creamy	43,757.77 ± 2425.70	41,912.33 ± 2762.13
68	4‐Methylthiazole	Heterocyclic compound	Nutty, green, vegetable, tomato	20.46 ± 1.90	18.94 ± 1.95
69	4‐Nonenal, (E)—	Aldehyde	Fruity	48.99 ± 31.38	58.03 ± 5.00
70	4‐Phenyl‐2‐butanol	Alcohol	Floral, peony, foliage, sweet, mimosa, heliotrope	126.58 ± 6.19	109.78 ± 1.93
71	4‐Undecanone	Ketone	Fruity	1.37 ± 0.079	1.34 ± 0.11
72	5,9‐Undecadien‐2‐one, 6,10‐dimethyl‐, (E)—	Ketone	Fresh, green, fruity, waxy, rose, woody, magnolia, tropical	1.63 ± 0.45	1.33 ± 0.32
73	5‐Methyl‐(E)‐2‐hepten‐4‐one	Ketone	Hazelnut, nutty	194,488.58 ± 7574.51	181,282.65 ± 7448.45
74	5‐Methyl‐2‐thiophenecarboxaldehyde	Heterocyclic compound	Sweet, almond, cherry, furfural, woody, acetophenone	422.17 ± 57.19	311.52 ± 131.21
75	5H‐5‐Methyl‐6,7‐dihydrocyclopentapyrazine	Heterocyclic compound	Earthy, baked, potato, peanut, roasted	36.75 ± 3.15	37.48 ± 2.04
76	6‐Nonenal, (E)—	Aldehyde	—	7303.85 ± 1205.70	5274.90 ± 1173.76
77	6‐Nonenal, (Z)—	Aldehyde	Green, cucumber, melon, cantaloupe, honeydew, waxy, vegetable, orris, violet, leafy	765.49 ± 533.20	1166.90 ± 95.19
78	Acetic acid, 2‐ethylhexyl ester	Ester	Earthy, herbal, humus, undergrowth	3.19 ± 0.36	2.99 ± 0.072
79	Acetic acid, cyclohexyl ester	Ester	Fruity, sweet, musty, ethereal	332.59 ± 0.95	277.50 ± 22.09
80	Acetic acid, phenyl ester	Ester	Phenol, medicinal, animalic, resinous, castoreum, woody, smoky, burnt	66.62 ± 2.58	59.55 ± 7.22
81	Acetophenone	Ketone	Sweet, pungent, hawthorn, mimosa, almond, acacia	2.95 ± 0.067	2.54 ± 0.19
82	Anethole	Aromatics	Sweet, exotic, flowery, stewed	14.23 ± 0.64	12.81 ± 0.39
83	BenzAldehyde	Aldehyde	Sweet, bitter, almond, cherry	3.56 ± 0.23	3.09 ± 0.23
84	BenzAldehyde, 2,5‐dimethyl—	Aldehyde	—	2.02 ± 0.23	1.55 ± 0.15
85	BenzAldehyde, 3‐hydroxy—	Aldehyde	—	9.72 ± 3.31	9.99 ± 1.73
86	BenzAldehyde, 4‐methoxy—	Aldehyde	Sweet, powdery, mimosa, floral, hawthorn, balsamic	383.85 ± 41.55	399.69 ± 38.52
87	Benzene, (2‐nitroethyl)—	Aromatics	flowery, spice	15.29 ± 0.81	13.08 ± 0.79
88	Benzene, (isothiocyanatomethyl)—	Sulfur compounds	Mild, watercress, dusty, medicinal, horseradish, oily	192.71 ± 4.11	172.30 ± 15.42
89	Benzene, 1,2,4,5‐tetramethyl—	Aromatics	Rancid, sweet	15.05 ± 2.33	12.97 ± 1.75
90	Benzene, 1‐ethyl‐3‐methyl—	Aromatics	—	12.88 ± 0.97	11.41 ± 1.21
91	BenzeneacetAldehyde	Aldehyde	Floral, honey, rose, cherry	859.49 ± 60.07	794.71 ± 44.00
92	Benzeneacetaldehyde, .alpha.‐ethylidene—	Aldehyde	Sweet, narcissus, cortex, beany, honey, cocoa, nutty, radish	1.01 ± 0.059	1.07 ± 0.081
93	Benzeneacetic acid	Acid	Sweet, honey, floral, honeysuckle, sour, waxy, civet	1.25 ± 0.80	1.58 ± 0.041
94	Benzeneacetic acid, ethyl ester	Ester	Minty	1.27 ± 1.00	0.33 ± 0.34
95	Benzenepropanoic acid, ethyl ester	Ester	Caramel, fruity	1.61 ± 0.19	1.36 ± 0.057
96	Benzofuran	Heterocyclic compound	Aromatic	5.09 ± 0.69	4.35 ± 0.28
97	Benzoic acid, 2‐(methylamino)‐, methyl ester	Ester	Fruity, musty, sweet, neroli, powdery, phenol, wine	3.41 ± 0.41	2.97 ± 0.45
98	Benzothiazole	Heterocyclic compound	Meaty, vegetable, brown, cooked, beefy, coffee	3.20 ± 0.96	3.34 ± 0.30
99	Benzyl Alcohol	Alcohol	Floral, rose, phenol, balsamic	2.30 ± 0.11	1.89 ± 0.25
100	Bicyclo[3.1.1]hept‐2‐ene‐2‐methanol, 6,6‐dimethyl—	Terpenoids	Woody, minty	27.37 ± 2.37	20.71 ± 5.97
101	Biphenyl	Aromatics	Pungent, rose, green, geranium	4.24 ± 0.64	3.61 ± 0.40
102	Bornyl acetate	Terpenoids	Woody, pine, herbal, cedary, spice	7.70 ± 0.30	6.84 ± 0.47
103	Butanoic acid, 2‐methyl‐, 2‐methylpropyl ester	Ester	Sweet, fruity	1.62 ± 0.13	1.46 ± 0.10
104	Butanoic acid, 2‐methyl‐, propyl ester	Ester	Winey, fruity, apple, pineapple	56.89 ± 6.39	39.62 ± 1.49
105	Butanoic acid, butyl ester	Ester	Fruity, banana, pineapple, green, cherry, tropical fruit, ripe fruit, juicy fruity	4.18 ± 0.15	3.87 ± 0.029
106	Camphor	Terpenoids	Camphor	76.55 ± 6.72	67.87 ± 4.14
107	Coumarin	Heterocyclic compound	Sweet, hay, tonka, new‐mown hay	2.21 ± 0.26	1.75 ± 0.13
108	Cyclohexanone, 5‐methyl‐2‐(1‐methylethyl)—	Terpenoids	Minty	57.42 ± 3.67	48.14 ± 3.46
109	d‐Limonene	Terpenoids	Citrus	11.54 ± 1.74	10.80 ± 1.84
110	Decanal	Aldehyde	Sweet, aldehydic, waxy, orange peel, citrus, floral	319.10 ± 32.22	336.99 ± 13.93
111	Decanoic acid, methyl ester	Ester	Oily, wine, fruity, floral	5.48 ± 1.58	4.74 ± 0.33
112	Dicyclopentadiene	Hydrocarbons	—	1590.21 ± 70.65	1433.68 ± 62.44
113	Diethyl diSulfur compounds	Sulfur compounds	Gassy, ripe onion, greasy, garlic	3352.86 ± 143.06	3123.28 ± 328.69
114	Dimethyl triSulfur compounds	Sulfur compounds	Sulfury, cooked onion, savory, meaty	31,483.89 ± 2347.80	31,803.79 ± 4304.19
115	Ethanone, 1‐(2‐aminophenyl)—	Ketone	Grape, sweet	98,181.29 ± 6933.50	95,578.02 ± 1908.08
116	Ethanone, 1‐(2‐furanyl)—	Heterocyclic compound	Nutty, sweet, roasted	2.31 ± 0.15	2.09 ± 0.024
117	Ethanone, 1‐(2‐pyridinyl)—	Heterocyclic compound	Popcorn, heavy, corn, chip, fatty, tobacco	5.77 ± 0.18	4.69 ± 0.22
118	Ethyl 2‐hexenoate, trans—	Ester	Green, fruity, tropical, juicy, papaya, quince, winey, rummy, orange, vegetable	1.65 ± 0.67	0.60 ± 0.73
119	Eugenol	Phenol	Floral, clove	367.69 ± 8.11	318.57 ± 23.73
120	Fenchol	Terpenoids	Camphor, borneol, pine, woody, dry, sweet, lemon	38.62 ± 11.39	27.65 ± 1.28
121	Geranyl acetate	Terpenoids	Lemon	5.26 ± 0.23	4.90 ± 0.25
122	Geranyl formate	Ester	Fresh, rose, neroli, tea, rose, green	1.68 ± 0.083	1.46 ± 0.069
123	Germacrene D	Terpenoids	Woody, spice	19.26 ± 3.15	16.57 ± 1.01
124	Hexanal	Aldehyde	Aldehyde, grassy, green, leafy, vinegar	28.71 ± 1.70	34.86 ± 3.90
125	Hexanoic acid, 2‐methylbutyl ester	Ester	Ethereal	1.04 ± 0.22	1.09 ± 0.30
126	Hexanoic acid, ethyl ester	Ester	Apple, pear, fruity	138.84 ± 95.89	82.21 ± 93.03
127	Indane	Aromatics	—	2.74 ± 0.21	2.26 ± 0.31
128	Indole	Heterocyclic compound	Animalic, floral, moth, mothball, fecal, naphthelene	68.47 ± 7.47	63.93 ± 4.07
129	Indole, 3‐methyl—	Heterocyclic compound	Animalic, fecal, indole, civet	683.46 ± 79.34	532.33 ± 67.31
130	Isoborneol	Terpenoids	Balsamic, camphor, herbal, woody	52.59 ± 2.45	47.65 ± 2.80
131	Isobutyl isovalerate	Ester	Sweet, fruity, apple, raspberry, green, banana	2.05 ± 0.17	1.84 ± 0.13
132	Isophorone	Ketone	Cool, woody, sweet, green, camphor, fruity, musty, cedarwood, tobacco, leathery	35.19 ± 2.11	31.66 ± 3.66
133	l‐.alpha.‐Terpineol	Terpenoids	Lilac, floral, terpenic	3.76 ± 0.17	3.30 ± 0.26
134	Linalool	Terpenoids	Floral, green	12.84 ± 0.26	11.03 ± 1.07
135	Maltol	Heterocyclic compound	Sweet, caramel	21.56 ± 3.50	15.43 ± 1.01
136	Methyl ethyl diSulfur compounds	Sulfur compounds	Sulfury, truffle	408.28 ± 51.15	348.08 ± 15.50
137	Methyl methacrylate	Ester	Acrylate, aromatic, fruity	3.17 ± 0.26	3.02 ± 0.21
138	Methyl salicylate	Ester	Caramel, pepperminty	5.70 ± 1.87	5.19 ± 0.82
139	Naphthalene	Aromatics	Pungent, dry, tarry	6.72 ± 0.90	5.39 ± 0.53
140	Naphthalene, 1,2,3,5,6,8a‐hexahydro‐4,7‐dimethyl‐1‐(1‐methylethyl)‐, (1S‐cis)—	Terpenoids	Thyme, herbal, woody, dry	9.79 ± 1.75	7.24 ± 0.34
141	Naphthalene, 1,2‐dihydro‐1,1,6‐trimethyl—	Aromatics	Licorice	26.08 ± 2.64	21.59 ± 1.99
142	Nonanoic acid	Acid	Waxy, dirty, cheese, cultured, dairy	28.21 ± 3.98	24.13 ± 1.63
143	Octanal	Aldehyde	Lemon, citrus, green grass	421.83 ± 19.61	441.13 ± 28.56
144	Pentanoic acid, 2‐methyl‐, ethyl ester	Ester	Fresh fruit, green, melon, apple skin, pineapple, natural, waxy	97780.94 ± 3579.76	77610.62 ± 21550.98
145	Pentanoic acid, 4‐methyl‐, ethyl ester	Ester	Fruity	58.83 ± 10.18	57.18 ± 2.29
146	Phenol	Phenol	Phenol, medicinal	19.51 ± 1.15	16.65 ± 1.92
147	Phenol, 2,4‐dichloro—	Phenol	—	822.02 ± 27.71	719.36 ± 38.86
148	Phenol, 2‐ethyl—	Phenol	Phenol	3.37 ± 0.26	2.93 ± 0.19
149	Phenol, 2‐methoxy‐4‐propyl—	Aromatics	Clove, sharp, spicy, sweet, phenol, powdery, allspice	153.12 ± 44.20	99.06 ± 16.34
150	Phenol, 2‐methyl—	Phenol	Phenol	98.92 ± 8.59	82.25 ± 8.15
151	Phenol, 2‐nitro—	Phenol	—	119.97 ± 2.75	106.30 ± 9.48
152	Phenol, 3‐ethyl—	Phenol	Musty	168.09 ± 38.84	172.36 ± 14.03
153	Phenol, 4‐ethyl‐2‐methoxy—	Phenol	Clove, candy	384.72 ± 58.87	258.94 ± 10.53
154	Phenol, m‐tert‐butyl—	Phenol	—	1.70 ± 0.14	1.25 ± 0.32
155	Propanoic acid, 2‐methyl‐, 2‐methylbutyl ester	Ester	Fruity, ethereal, tropical, banana	12.80 ± 8.33	16.71 ± 2.69
156	Pyrazine, 2,3‐dimethyl—	Heterocyclic compound	Nutty, nut skin, cocoa, peanut, buttery, coffee, walnut, caramel, roasted	39.20 ± 2.34	37.56 ± 7.34
157	Pyrazine, 2,3‐dimethyl‐5‐(1‐methylpropyl)—	Heterocyclic compound	Marine, burnt, roasted	2.00 ± 0.064	2.13 ± 0.25
158	Pyrazine, 2,5‐dimethyl—	Heterocyclic compound	Cocoa, roasted, nutty, roasted, beefy, woody, grassy, medicinal	77.91 ± 4.56	69.45 ± 3.27
159	Pyrazine, 2,6‐dimethyl—	Heterocyclic compound	Ethereal, cocoa, nutty, roasted, roasted, meaty, beefy, brown, coffee, buttermilky	13.63 ± 0.80	12.15 ± 0.57
160	Pyrazine, 2‐ethyl‐3,5‐dimethyl—	Heterocyclic compound	Burnt, almond, roasted, nutty, coffee	327,975.28 ± 40,657.18	331,810.36 ± 47,438.08
161	Pyrazine, 2‐ethyl‐3‐methyl—	Heterocyclic compound	Nutty, peanut, musty, corn, raw, earthy, bread	304.75 ± 13.35	279.89 ± 14.29
162	Pyrazine, 2‐ethyl‐5‐methyl—	Heterocyclic compound	Coffee, beany, nutty, grassy, roasted	4571.23 ± 200.26	4198.37 ± 214.33
163	Pyrazine, 2‐methoxy‐3‐(1‐methylethyl)—	Heterocyclic compound	Beany, pea, earthy, chocolate, nutty	757,826.08 ± 29,958.56	842,814.66 ± 112,376.41
164	Pyrazine, 2‐methoxy‐3‐(1‐methylpropyl)—	Heterocyclic compound	Musty, green, pea, galbanum, bell pepper, pepper	566,233.28 ± 50,382.39	503,826.21 ± 60,577.92
165	Pyrazine, 2‐methoxy‐3‐methyl—	Heterocyclic compound	Roasted almond, hazelnut, peanut	68.94 ± 13.54	56.19 ± 20.77
166	Pyrazine, 2‐methoxy‐6‐methyl—	Heterocyclic compound	Roasted hazelnut, almond, peanut	12.17 ± 2.39	9.92 ± 3.66
167	Pyrazine, 2‐methyl‐3‐(methylthio)—	Heterocyclic compound	Roasted meat, nutty, almond, vegetable	5147.19 ± 343.49	4671.26 ± 293.16
168	Pyrazine, 2‐methyl‐6‐(methylthio)—	Heterocyclic compound	—	257.36 ± 17.17	233.56 ± 14.66
169	Pyrazine, 3,5‐diethyl‐2‐methyl—	Heterocyclic compound	Nutty, meaty, vegetable	39.50 ± 4.31	36.61 ± 2.01
170	Pyrazine, 3‐ethyl‐2,5‐dimethyl—	Heterocyclic compound	Potato, cocoa, roasted, nutty	1525.47 ± 189.10	1543.30 ± 220.64
171	Pyrazine, methyl—	Heterocyclic compound	Nutty, cocoa, roasted, chocolate, peanut, green	1204.60 ± 50.08	1090.15 ± 48.93
172	Pyrazine, trimethyl—	Heterocyclic compound	Nut skin, earthy, powdery, cocoa, baked, potato, roasted, peanut, hazelnut, musty	14.13 ± 1.29	11.60 ± 1.50
173	Pyridine, 2‐ethyl—	Heterocyclic compound	Green, grassy	14.64 ± 0.44	13.81 ± 0.24
174	Pyridine, 2‐pentyl—	Heterocyclic compound	Fatty, tallow, green, pepper, mushroom, herbal	897.84 ± 258.34	844.92 ± 149.86
175	Styrene	Aromatics	Penetrating, balsamic, gasoline	30.69 ± 5.77	25.60 ± 5.94
176	TRANS‐ANETHOLE	Aromatics	Sweet, anisic, licorice, mimosa	3.74 ± 0.17	3.37 ± 0.10
177	Thiazole, 2,4,5‐trimethyl—	Heterocyclic compound	Musty, nutty, vegetable, cocoa, hazelnut, chocolate, coffee	1.68 ± 0.54	1.67 ± 0.18
178	Thymol	Terpenoids	Herbal, thyme, phenol, medicinal, camphor	5.14 ± 0.21	4.60 ± 0.50
179	Vanillin	Aldehyde	Sweet, vanilla, creamy, chocolate	12.23 ± 1.88	8.97 ± 0.20
180	n‐Decanoic Acid	Acid	Fatty, rancid, soapy, unpleasant, rancid, sour, fatty, citrus	1.13 ± 0.42	0.82 ± 0.077
181	p‐Cresol	Phenol	Phenol, narcissus, animalic, mimosa	13,335.77 ± 153.58	12,518.12 ± 1704.67
182	trans, cis‐2,6‐Nonadien‐1‐ol	Alcohol	Green, cucumber, oily, violet, leafy	47.59 ± 1.32	43.05 ± 2.33
183	trans‐.beta.‐Ocimene	Terpenoids	Sweet, herbal	4.53 ± 2.49	2.69 ± 2.09
184	trans‐Isoeugenol	Phenol	Floral, clove	48.67 ± 8.18	46.60 ± 3.50

**FIGURE 7:**
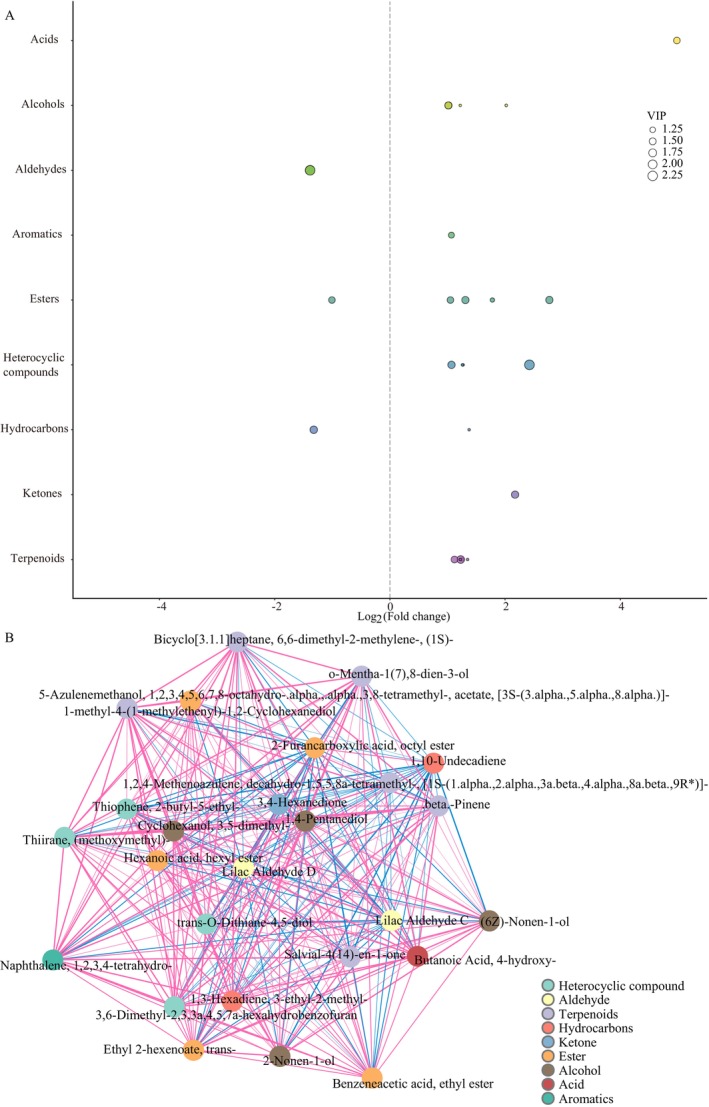
The differentially changed volatile compounds (DCVCs) between PE versus PB. A total of 26 DCVCs were found between PE and PB, including 22 upregulated DCVCs and 4 downregulated DCVCs (A). Interaction analysis of DCVCs (B), in which the red line represents positive correlation, the blue line represents negative correlation, and thicker solid lines mean stronger correlation.

To gain further insights on the change of enhanced fermentation with *P. membranifaciens*, the differentially changed volatile compounds (DCVCs) with variable importance in projection (VIP) > 1.0, *p* < 0.05, and FC > 2.0 or FC < 0.50, between different groups in PB and PE were assessed and identified, as shown in Figure 7A. A total of 26 DCVCs (six terpenoids, five esters, four alcohols, four heterocyclic compounds, two hydrocarbons, two aldehydes, one acid, one aromatic, and one ketone) were found, including 22 upregulated DCVCs and 4 downregulated DCVCs. The upregulated DCVCs included (6Z)‐nonen‐1‐ol; β‐pinene; 2‐nonen‐1‐ol; 1,2,4‐methenoazulene, decahydro‐1,5,5,8a‐tetramethyl‐, [1S‐(1.alpha.,2.alpha.,3a.beta.,4.alpha.,8a.beta.,9R*)]‐; 1,3‐hexadiene, 3‐ethyl‐2‐methyl‐; 1,4‐pentanediol; 1‐methyl‐4‐(1‐methylethenyl)‐1,2‐cyclohexanediol; 3,4‐hexanedione; 3,6‐dimethyl‐2,3,3a,4,5,7a‐hexahydrobenzofuran; 5‐azulenemethanol, 1,2,3,4,5,6,7,8‐octahydro‐.alpha.,.alpha.,3,8‐tetramethyl‐, acetate, [3S‐(3.alpha.,5.alpha.,8.alpha.)]‐; benzeneacetic acid, ethyl ester; bicyclo[3.1.1]heptane, 6,6‐dimethyl‐2‐methylene‐, (1S)‐; butanoic acid, 4‐hydroxy‐; cyclohexanol, 3,5‐dimethyl‐; ethyl 2‐hexenoate, trans‐; hexanoic acid, hexyl ester; naphthalene, 1,2,3,4‐tetrahydro‐; salvial‐4(14)‐en‐1‐one; thiirane, (methoxymethyl)‐; thiophene, 2‐butyl‐5‐ethyl‐; o‐mentha‐1(7),8‐dien‐3‐ol; and trans‐O‐dithiane‐4,5‐diol. Four downregulated DCVCs included 1,10‐undecadiene; 2‐furancarboxylic acid, octyl ester; lilac aldehyde C; and lilac aldehyde D. 16 DCVCs showed odor characteristics, for example, 3,4‐hexanedione; hexanoic acid, hexyl ester; lilac aldehyde C; lilac aldehyde D; thiophene, 2‐butyl‐5‐ethyl‐; etc. Moreover, 3 DCVCs ((6Z)‐nonen‐1‐ol; benzeneacetic acid, ethyl ester; and ethyl 2‐hexenoate, trans‐) directly contributed to coffee flavor.

A correlation analysis between 26 DCVCs was carried out to obtain more useful information about the interaction of DCVCs on enhanced fermentation with *P. membranifaciens*, as shown in Figure 7B. Based on the value of the correlation coefficient (*r*), 0.8–1.0 indicated an extremely strong correlation, and 0.6–0.8 indicated a strong correlation (Hu et al. 2020). 1,4‐pentanediol with benzeneacetic acid, ethyl ester; lilac aldehyde C with lilac aldehyde D; (6Z)‐nonen‐1‐ol with 2‐nonen‐1‐ol showed extremely strongly positive correlation with the value of r 1.0. Beta‐pinene was extremely strongly positively correlated with 1,3‐hexadiene, 3‐ethyl‐2‐methyl‐; 3,6‐dimethyl‐2,3,3a,4,5,7a‐hexahydrobenzofuran; bicyclo[3.1.1]heptane, 6,6‐dimethyl‐2‐methylene‐(1S)‐; cyclohexanol, 3,5‐dimethyl‐; thiirane, (methoxymethyl)‐; and o‐mentha‐1(7),8‐dien‐3‐ol (*r* = 1.0). 3,6‐dimethyl‐2,3,3a,4,5,7a‐hexahydrobenzofuran was extremely strongly positively correlated with bicyclo[3.1.1]heptane, 6,6‐dimethyl‐2‐methylene‐, (1S)‐; thiirane, (methoxymethyl)‐; and o‐mentha‐1(7),8‐dien‐3‐ol (*r* = 1.0). 1,3‐hexadiene, 3‐ethyl‐2‐methyl‐ was extremely strongly positively correlated with 3,6‐dimethyl‐2,3,3a,4,5,7a‐hexahydrobenzofuran; o‐mentha‐1(7),8‐dien‐3‐ol; thiirane, (methoxymethyl)‐; cyclohexanol, 3,5‐dimethyl‐; and bicyclo[3.1.1]heptane, 6,6‐dimethyl‐2‐methylene‐, (1S)‐ (*r* = 1.0). While 1‐methyl‐4‐(1‐methylethenyl)‐1,2‐cyclohexanediol was extremely strongly negatively correlated with 2‐furancarboxylic acid, octyl ester (*r* = −1.0).

### 3.4 The Result of Sensory Characteristics Analysis

**FIGURE 8:**
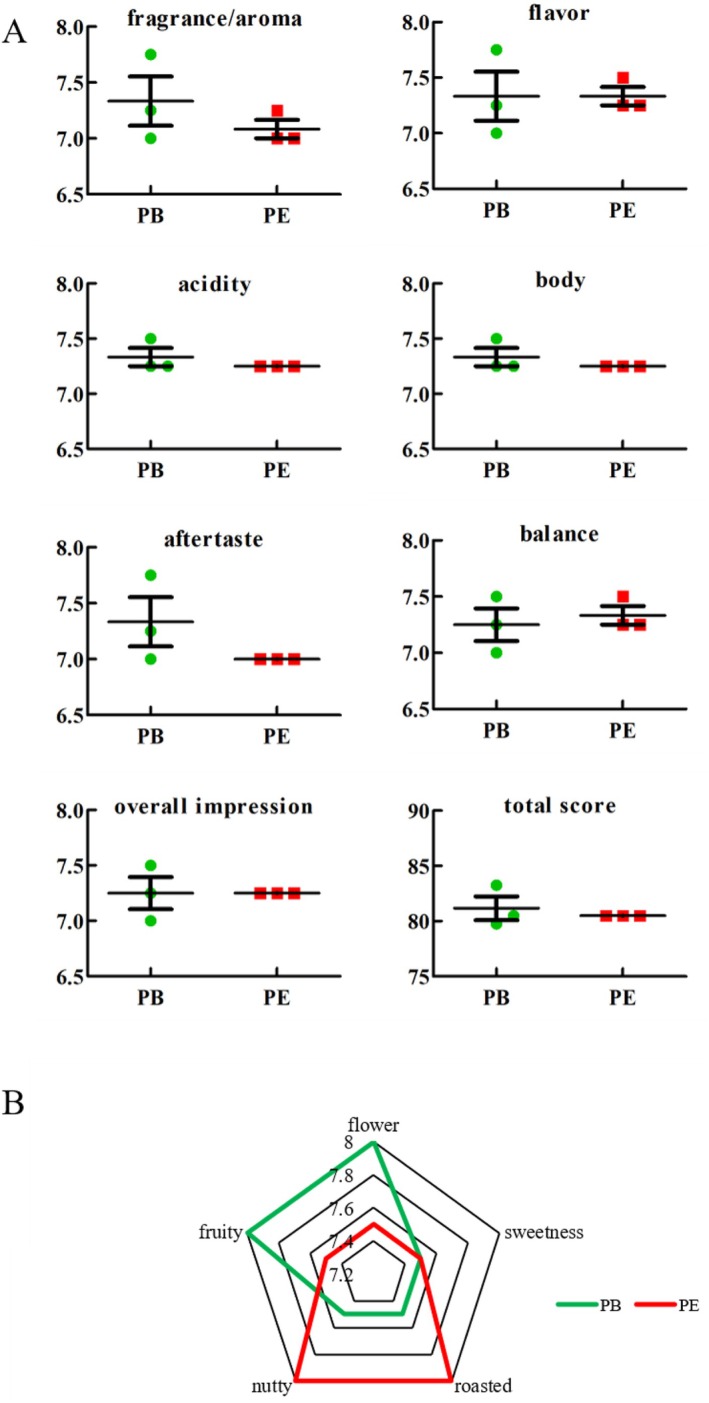
The sensory score of SCA cupping protocol (A) and flavor characteristics (B) of coffee samples.

According to the result of sensory analysis (Figure 8A), the total score of PE was 80.05 ± 0.00. When the total score is over 80, the coffee is classified as fine (Cassimiro et al. 2023). Therefore, PB and PE of *C. arabica* were fine. Although the total score of the PE group was lower than the PB group, the scores were more stable than those of PB. Meanwhile, the intensity of roasted and nutty aromas of PE was higher than that of PB (Figure 8B).

## 4 Discussion

Microbial fermentation of coffee may modulate or confer additional aroma notes. Fermentation is believed to affect coffee flavor. Usually, fermented coffee under suitable condition shows better quality attributes than unfermented coffee (Elhalis et al. 2023). Therefore, proper starter culture and fermentation condition optimization in coffee fermentation can impart targeted modulations on coffee flavor‐related constituents to significantly improved coffee flavor, and even produce novel, sensory quality (Wang et al. 2020). In recent years, enhanced fermentation has become a symbol of modern fermentation technology, which is an innovative and effective fermentation strategy for improving food flavor and security, shortening fermentation periods (Shen, Wang, Yuan, et al. 2025; Zheng et al. 2024; Cao et al. 2022; Li et al. 2022). Enhanced fermentation can produce antibacterial substances to inhibit the growth of pathogens, spoilage microorganisms, and other microorganisms during fermentation (Zheng et al. 2024; Li et al. 2022). Then, this inhibition function increased the stability of the fermentation process (Li et al. 2022). *Pichia membranifaciens* is a nonpathogenic and safe yeast that can directly inhibit or secrete extracellular metabolites to inhibit pathogens and not affect beneficial organisms. Simultaneously, *P. membranefaciens* does not produce obvious antibiotic substances (Zhang et al. 2025). Based on the effectively inhibition on a variety of pathogens, *P. membranifaciens* has been widely used in postharvest biological control of fruits and vegetables for preventing or slowing down perish due to microbiological diseases, disorders, transpiration, and senescence (Cao et al. 2008; Chan and Tian 2005). During coffee postharvest processing phase, fermentation condition is carried out in a developed environment which result in a dynamic change on microbial communities by their complex interaction (Shen, Wang, Zheng, et al. 2025; Shen, Yuan, et al. 2024; Shen, Zi, et al. 2023). Therefore, microbial communities often are influenced by environmental factors, such as the coffee region, temperature, altitude, pH, and so on (Shen, Wang, et al. 2024). Therefore, the control of fermentation conditions is a prerequisite for improving coffee quality, such as suitable fermentation duration, processing type, application of soaking, etc. (Zhang et al. 2019; Ferreira et al. 2023). At the level of fungal genera in PE, *Pichia* was the dominant genus, and *P. membranefaciens* was the dominant species. Compared with PB, *P. membranefaciens* significantly inhibited the growth of microorganisms, such as *Hanseniaspora*, *Lachancea*, *Candida*, *Cystofilobasidium*, *Aschetsonia*, *Apiotrichum*, and others in fermentation. At the same time, *P. membranefaciens* inhibited the growth of *Pantoea*, *Trichococcus*, and *Lactobacillus*, while promoting the growth of *Weissella* at the genus level. Overall, enhanced fermentation with *P. membranefaciens* contributed to the change in microbial communities to keep related stable of microbial communities in fermentation.

In addition, coffee fermentation degraded coffee mucilage, removed coffee layers, and produced metabolites to change coffee chemical compounds by microorganisms (Ferreira et al. 2023). Based on the change of microbial community structures by enhanced fermentation with *P. membranefaciens*, their metabolites also changed, which resulted in coffee flavor precursors changing, such as sugar, proteins, amino acids, and phenolic compounds. For example, *P. membranifaciens* could produce 4‐ethylphenol (Saez et al. 2011). OPLS‐DA is a powerful statistical modeling tool for distinguishing predictions and orthogonal components to explain the variation between and within groups. OPLS‐DA can eliminate data irrelevant to category information (orthogonal), and more easily exclude independent variables unrelated to classification and screen out characteristic variables of samples. In addition, OPLS‐DA serves as a supervised recognition method that can be used to obtain the best classification and establish the discriminant models (Kang et al. 2022; Boccard and Rutledge 2013). According to the results of UHPLC–MS/MS analysis combined with OPLS‐DA, 122 and 122 DCn‐VCs were found in PE2 versus PB2, and PE3 versus PB3, respectively. These compounds mainly included lipids and lipids‐like molecules, organic acids and derivatives, organoheterocyclic compounds, organic oxygen compounds, phenylpropaniioids and polyketides, etc. For example, 4‐feruloyl‐1,5‐quinolactone increased with FC 1.53, methyl 3,4‐dicaffeoylquinate increased with FC 1.92. Indolelactic acid (FC = 4.98), 3‐phenyllactic acid (FC = 2.30) also increased. Maillard reaction is important reaction for coffee aroma formation, in which amino acids and reducing sugars participate during roasting (Lee et al. 2015). Amino acids as key flavor precursors of coffee aroma formation, aspartic acid (FC = 0.52), and 5‐aminovaleric acid (FC = 0.52) decreased in enhanced fermentation with *P. membranifaciens*. In addition, azelaic acid increased with the value of FC 2.18, which exhibits the capacity of bacteriostatic, anti‐inflammatory (Yu et al. 2024).

Coffee flavor precursors form coffee aroma through Maillard reactions, Strecker degradation, caramelization, and fragmentation reactions (Lee et al. 2015). Aroma volatile chemicals in roasted coffee are the most important quality determinant compounds (Sunarharum et al. 2014). In coffee, more than 1000 volatile compounds have been identified, but only a small number of them contribute to the coffee flavor and aroma (Sunarharum et al. 2014). Furanones (e.g., furfural, furfuryl acetate, 5‐methylfurfural, 5‐hydroxymethylfurfural, etc.), phenolic compounds (e.g., vanillin, 4‐ethylguaiacol, 2‐methoxy‐4‐vinylphenol, guaiacol, 4‐vinylguaiacol, etc.), sulfur‐containing compounds (e.g., 3‐methyl‐2‐butene‐1‐thiol, 2‐furfurylthiol, 2‐methyl‐3‐furanthiol, etc.), and pyrazines (e.g., 2,5‐dimethylpyrazine, 2,6‐dimethylpyrazine, 2‐ethylpyrazine, 2,3‐dimethylpyrazine, etc.) importantly contribute to coffee flavor (Sunarharum et al. 2014). Although 917 VCs were detected in this study, 475 VCs showed aroma characteristics, and 184 VCs directly contributed to coffee flavor based on rOAV. However, 169 VCs from 184 odor compounds were directly aroma compounds that can contribute to coffee flavor. Compared with PB, 3 upregulated DCVCs (e.g., (6Z)‐nonen‐1‐ol; benzeneacetic acid, ethyl ester; and ethyl 2‐hexenoate, trans‐) were directly odor characteristic DCVCs. (6Z)‐nonen‐1‐ol contributed to odor characteristics of fruits and vegetables of coffee, including fresh, green, melon, waxy, honeydew, cantaloupe, cucumber, and clean. Benzeneacetic acid contributed to a minty coffee flavor. Ethyl 2‐trans‐hexenoate, trans‐ contributed to green, fruity, tropical, juicy, papaya, quince, winey, rummy, orange, and vegetable coffee flavor. Moreover, the correlation analysis showed, (6Z)‐nonen‐1‐ol with benzeneacetic acid, and ethyl 2‐hexenoate, trans, ethyl 2‐hexenoate, trans‐ with benzeneacetic acid, ethyl ester were positively correlated, which can improve the odor characteristics each other. Only (6Z)‐Nonen‐1‐ol showed a negative correlation with 1,10‐undecadiene, which is not an odor compound. Overall, enhanced fermentation with *P. membranefaciens* changed microbial communities by inhibiting other microorganisms' growth in fermentation. Finally, this function changed the coffee compounds to stabilize coffee sensory.

The quality of the coffee beverage is considered a consolidated criterion for reaching the markets, which is evaluated using SCA cupping protocol (Ribeiro et al. 2017; Rocha et al. 2023). The SCA cupping protocol not only defines standardized methodologies to assist buyers and producers in evaluating the sensory quality of coffee, especially for fair and more attractive trade, but also is the only one to define specialty coffee for the international market and coffee fermentation researchers (Ferreira et al. 2023). According to the results of the SCA cupping protocol, fermentation with *P. membranefaciens* not only produces fine coffee but also keeps stable coffee flavor to avoid the influence of fluctuating fermentation conditions under an open environment. To face the whole coffee market consumption, the coffee flavor characteristics accepted by a trained assessor are not enough. To better enter the coffee consumer market and be accepted by consumers, sensory tests by consumers are also important (CarolinaVieira‐Porto et al. 2024; DePaula et al. 2023). The flavor characteristics evaluated showed flower, sweetness, roasted, nuts, and fruity aroma of coffee fermentation with *P. membranefaciens* were liked by assessors, which means the coffee fermentation with *P. membranefaciens* will have a high acceptance possibility in the future.

Therefore, *P. membranefaciens* is a potential stater in coffee fermentation to utilize for control fermentation condition and keep coffee flavor.

## 5 Conclusion

The changes in microbial community structure, nonvolatile compounds (nVCs) during enhanced fermentation with *P. membranefaciens*, and volatile compounds (VCs) and coffee sensory of roasted coffee beans were evaluated in this study. Compared to *Hanseniaspora* and *Hanseniaspora meyeri* in PB, the predominant genus and species were *Pichia* and *P. membranifaciens* in PE, respectively. At the same time, a total of 122 and 122 DCn‐VCs were found between PE2 versus PB2 and PE3 versus PB3, respectively. Furthermore, 26 DCVCs were found between PE and PB. Among them, benzeneacetic acid, ethyl ester was positively correlated with (6Z)‐nonen‐1‐ol, ethyl 2‐hexenoate, trans‐. In summary, enhanced fermentation with *P. membranefaciens* stabilized the microbial community structure by inhibiting the growth of other microorganisms during the fermentation, which resulted in the changes of the chemical compounds during the fermentation and volatile compounds of coffee finally.

## Author Contributions

**Xiaojing Shen:** data curation (equal), methodology (equal), writing – original draft (equal). **Qi Wang:** data curation (equal), methodology (equal), writing – original draft (equal). **Jia Zheng:** methodology (equal), writing – review and editing (equal). **Xingyu Li:** resources (equal), software (equal). **Song Li:** resources (equal), software (equal). **Yanhua Yin:** resources (equal), software (equal). **Mengli Shang:** resources (equal), software (equal). **Kunyi Liu:** methodology (equal), project administration (equal), writing – review and editing (equal). **Wenjuan Yuan:** methodology (equal), writing – review and editing (equal). **Jilai Zhang:** methodology (equal), writing – review and editing (equal).

## Conflicts of Interest

The authors declare no conflicts of interest.

## Supporting information


**TABLE S1.** The relative content of volatile compounds in PE and PB.
**TABLE S2.** The odor of volatile compounds in coffee samples.

## Data Availability

The data that support the findings of this study are available from the corresponding author upon reasonable request.
