# Supplementary material for: Effect on Arabica Coffee Flavor Quality of Enhanced Fermentation With Pichia membranifaciens Through Change Microbial Communities and Chemical Compounds
Source: Food Sci Nutr. 2025 Jun 28;13(7):e70512. doi: 10.1002/fsn3.70512 (PMC12205213; doi:10.1002/fsn3.70512)
Supplement: Supplementary file 1 — TABLE S1. The relative content of volatile compounds in PE and PB. TABLE S2. The odor of volatile compounds in coffee samples. [file FSN3-13-e70512-s001.docx]

**Table S1.** The relative content of volatile compounds in PE and PB.

| No. | Compounds | Class I | PE/(mg/kg) | | PB/(mg/kg) | | |  |
| --- | --- | --- | --- | --- | --- | --- | --- | --- |
|  |  |  | Mean | SD | | Mean | SD | |
| 1 | (+)-3-Carene | Terpenoids | 0.36 | 0.02 | | 0.23 | 0.15 | |
| 2 | (+)-4-Carene | Terpenoids | 0.36 | 0.02 | | 0.23 | 0.15 | |
| 3 | (+)-Diethyl L-tartrate | Ester | 0.05 | 0.00 | | 0.04 | 0.00 | |
| 4 | (+)-cis-Verbenol, acetate | Ester | 0.05 | 0.00 | | 0.06 | 0.03 | |
| 5 | (+)-epi-Bicyclosesquiphellandrene | Terpenoids | 0.02 | 0.00 | | 0.02 | 0.00 | |
| 6 | (-)-.beta.-Bourbonene | Terpenoids | 0.16 | 0.02 | | 0.15 | 0.02 | |
| 7 | (-)-Myrtenol | Terpenoids | 1.07 | 0.25 | | 1.01 | 0.01 | |
| 8 | (1.alpha.,2.beta.,5.alpha.)-2-methyl-5-(1-methylethenyl)-Cyclohexanol | Terpenoids | 0.05 | 0.01 | | 0.05 | 0.00 | |
| 9 | (1.alpha.,4a.beta.,8a.alpha.)-1,2,3,4,4a,5,6,8a-octahydro-7-methyl-4-methylene-1-(1-methylethyl)-Naphthalene | Terpenoids | 0.05 | 0.01 | | 0.03 | 0.00 | |
| 10 | (1Alpha,3beta,4beta)-p-menthane-3,8-diol | Terpenoids | 0.04 | 0.00 | | 0.04 | 0.00 | |
| 11 | (1R)-(-)-Myrtenal | Aldehyde | 0.20 | 0.02 | | 0.18 | 0.02 | |
| 12 | (1R,1aR,2aS,5R,6R,6aS,7aS)-1,6,6a-trimethyldecahydro-1,2a-methanocyclopropa[b]naphthalen-5-ol | Terpenoids | 0.01 | 0.00 | | 0.01 | 0.00 | |
| 13 | (1R,2R,4S,6S,7S,8S)-8-Isopropyl-1-methyl-3-methylenetricyclo[4.4.0.02,7]decan-4-ol | Terpenoids | 0.02 | 0.00 | | 0.01 | 0.00 | |
| 14 | (1R,3aR,4aR,8aR)-1,4,4,6-Tetramethyl-1,2,3,3a,4,4a,7,8-octahydrocyclopenta[1,4]cyclobuta[1,2]benzene | Terpenoids | 0.09 | 0.01 | | 0.07 | 0.00 | |
| 15 | (1R,3aR,5aR,9aS)-1,4,4,7-Tetramethyl-1,2,3,3a,4,5a,8,9-octahydrocyclopenta[c]benzofuran | Terpenoids | 0.00 | 0.00 | | 0.00 | 0.00 | |
| 16 | (1R,3aS,4aS,8aS)-1,4,4,6-Tetramethyl-1,2,3,3a,4,4a,7,8-octahydrocyclopenta[1,4]cyclobuta[1,2]benzene | Terpenoids | 0.09 | 0.01 | | 0.07 | 0.00 | |
| 17 | (1R,3aS,5aS,8aR)-1,3a,4,5a-Tetramethyl-1,2,3,3a,5a,6,7,8-octahydrocyclopenta[c]pentalene | Terpenoids | 0.06 | 0.00 | | 0.05 | 0.00 | |
| 18 | (1R,3aS,8aS)-7-Isopropyl-1,4-dimethyl-1,2,3,3a,6,8a-hexahydroazulene | Terpenoids | 0.03 | 0.00 | | 0.03 | 0.00 | |
| 19 | (1R,4R,4aS,8aR)-4,7-Dimethyl-1-(prop-1-en-2-yl)-1,2,3,4,4a,5,6,8a-octahydronaphthalene | Terpenoids | 0.05 | 0.00 | | 0.04 | 0.00 | |
| 20 | (1R,5S,8R)-2,4,4,8-tetramethyltricyclo[3.3.3.01,5]undec-2-ene | Terpenoids | 0.00 | 0.00 | | 0.00 | 0.00 | |
| 21 | (1S,4S,4aR)-1-Isopropyl-4-methyl-7-methylene-1,2,3,4,4a,5,6,7-octahydronaphthalene | Terpenoids | 0.02 | 0.00 | | 0.02 | 0.01 | |
| 22 | (1S,4S,4aS)-1-Isopropyl-4,7-dimethyl-1,2,3,4,4a,5-hexahydronaphthalene | Terpenoids | 0.02 | 0.00 | | 0.02 | 0.01 | |
| 23 | (1S,4aR,7R)-1,4a-Dimethyl-7-(prop-1-en-2-yl)-1,2,3,4,4a,5,6,7-octahydronaphthalene | Terpenoids | 0.08 | 0.01 | | 0.07 | 0.01 | |
| 24 | (1S,5S)-2-Methyl-5-((R)-6-methylhept-5-en-2-yl)bicyclo[3.1.0]hex-2-ene | Hydrocarbons | 0.09 | 0.01 | | 0.07 | 0.00 | |
| 25 | (1S,5S,6R)-6-Methyl-2-methylene-6-(4-methylpent-3-en-1-yl)bicyclo[3.1.1]heptane | Terpenoids | 0.02 | 0.01 | | 0.01 | 0.00 | |
| 26 | (2,2,6-Trimethyl-bicyclo[4.1.0]hept-1-yl)-methanol | Alcohol | 0.05 | 0.00 | | 0.05 | 0.00 | |
| 27 | (2E)-2-(Acetylhydrazono)propanoic acid | Acid | 0.03 | 0.01 | | 0.03 | 0.01 | |
| 28 | (2E,4Z)-2,4-Decadienal | Aldehyde | 0.42 | 0.10 | | 0.43 | 0.03 | |
| 29 | (2S,4aR,8aR)-4a,8-Dimethyl-2-(prop-1-en-2-yl)-1,2,3,4,4a,5,6,8a-octahydronaphthalene | Terpenoids | 0.01 | 0.00 | | 0.01 | 0.00 | |
| 30 | (2S,6R,7S,8E)-(+)-2,7-Epoxy-4,8-megastigmadiene | Heterocyclic compound | 0.35 | 0.07 | | 0.29 | 0.04 | |
| 31 | (3R,3aR,7S,8aS)-3,6,8,8-Tetramethyl-4,7,8,8a-tetrahydro-1H-3a,7-methanoazulen-2(3H)-one | Terpenoids | 0.02 | 0.01 | | 0.02 | 0.00 | |
| 32 | (3R,6R)-2,2,6-Trimethyl-6-vinyltetrahydro-2H-pyran-3-ol | Heterocyclic compound | 0.14 | 0.01 | | 0.13 | 0.00 | |
| 33 | (3S,3aS,6R,8aS)-3,8,8-Trimethyl-7-methyleneoctahydro-1H-3a,6-methanoazulene | Terpenoids | 0.02 | 0.00 | | 0.02 | 0.00 | |
| 34 | (4R,4aR)-4,4a-Dimethyl-6-(prop-1-en-2-yl)-1,2,3,4,4a,7-hexahydronaphthalene | Terpenoids | 0.03 | 0.02 | | 0.04 | 0.00 | |
| 35 | (4R,4aS,6S)-4,4a-Dimethyl-6-(prop-1-en-2-yl)-1,2,3,4,4a,5,6,7-octahydronaphthalene | Terpenoids | 0.03 | 0.01 | | 0.02 | 0.01 | |
| 36 | (4aR-trans)-decahydro-4a-methyl-1-methylene-7-(1-methylethylidene)-Naphthalene | Terpenoids | 0.01 | 0.00 | | 0.01 | 0.00 | |
| 37 | (4aS-cis)-2,4a,5,6,7,8,9,9a-octahydro-3,5,5-trimethyl-9-methylene-1H-Benzocycloheptene | Terpenoids | 0.22 | 0.01 | | 0.20 | 0.02 | |
| 38 | (5-bromopentyl)-Benzene | Aromatics | 0.04 | 0.01 | | 0.03 | 0.00 | |
| 39 | (6,6-dimethyl-2-bicyclo[3.1.1]hept-2-enyl)methyl 2-methylpropanoate | Ester | 0.02 | 0.00 | | 0.02 | 0.01 | |
| 40 | (6Z)-Nonen-1-ol | Alcohol | 0.12 | 0.02 | | 0.05 | 0.06 | |
| 41 | (8R,8aS)-8,8a-Dimethyl-2-(propan-2-ylidene)-1,2,3,7,8,8a-hexahydronaphthalene | Terpenoids | 0.01 | 0.00 | | 0.01 | 0.00 | |
| 42 | (E)-2,6-Dimethylocta-2,5,7-trien-4-one | Ketone | 0.05 | 0.01 | | 0.03 | 0.00 | |
| 43 | (E)-2,6-Dimethylocta-5,7-dien-2-ol | Alcohol | 0.04 | 0.04 | | 0.06 | 0.04 | |
| 44 | (E)-2-Decenal | Aldehyde | 0.03 | 0.00 | | 0.02 | 0.00 | |
| 45 | (E)-2-Heptenal | Aldehyde | 0.06 | 0.02 | | 0.06 | 0.01 | |
| 46 | (E)-2-Methylbut-2-en-1-yl methacrylate | Ester | 0.63 | 0.01 | | 0.55 | 0.04 | |
| 47 | (E)-4,8-Dimethylnona-1,3,7-triene | Terpenoids | 0.02 | 0.00 | | 0.02 | 0.00 | |
| 48 | (E)-sec-Butyl propenyl diSulfur compounds | Sulfur compounds | 0.09 | 0.03 | | 0.09 | 0.00 | |
| 49 | (E,E)-2,4-Undecadienal | Aldehyde | 5.76 | 0.23 | | 4.98 | 0.10 | |
| 50 | (R)-(+)-1-(p-Tolyl)ethylAmine | Amine | 0.44 | 0.14 | | 0.34 | 0.11 | |
| 51 | (S)-(-)-(4-Isopropenyl-1-cyclohexenyl)methanol | Terpenoids | 0.02 | 0.00 | | 0.02 | 0.00 | |
| 52 | (Z)-2,2-Dimethyl-3-(3-methylpenta-2,4-dien-1-yl)oxirane | Terpenoids | 0.29 | 0.07 | | 0.20 | 0.05 | |
| 53 | (Z)-2,6-Dimethylocta-2,5,7-trien-4-one | Ketone | 0.14 | 0.02 | | 0.09 | 0.06 | |
| 54 | (Z)-2-Heptenal | Aldehyde | 0.06 | 0.02 | | 0.06 | 0.01 | |
| 55 | .alpha.-Cubebene | Terpenoids | 0.83 | 0.06 | | 0.72 | 0.05 | |
| 56 | .alpha.-Dehydro-ar-himachalene | Terpenoids | 0.05 | 0.00 | | 0.04 | 0.00 | |
| 57 | .alpha.-Ionone | Terpenoids | 0.08 | 0.01 | | 0.06 | 0.01 | |
| 58 | .alpha.-Irone | Terpenoids | 0.04 | 0.01 | | 0.03 | 0.00 | |
| 59 | .alpha.-Maaliene | Terpenoids | 0.01 | 0.00 | | 0.01 | 0.00 | |
| 60 | .alpha.-Methylstyrene | Aromatics | 0.01 | 0.00 | | 0.01 | 0.00 | |
| 61 | .alpha.-Muurolene | Terpenoids | 0.04 | 0.00 | | 0.03 | 0.00 | |
| 62 | .alpha.-Phellandrene 1 | Terpenoids | 0.36 | 0.02 | | 0.23 | 0.15 | |
| 63 | .alpha.-Terpineol | Terpenoids | 1.24 | 0.06 | | 1.09 | 0.09 | |
| 64 | .beta.-Acorenol | Terpenoids | 0.02 | 0.00 | | 0.02 | 0.00 | |
| 65 | .beta.-Longipinene | Terpenoids | 0.12 | 0.02 | | 0.11 | 0.02 | |
| 66 | .beta.-Ocimene | Terpenoids | 0.13 | 0.07 | | 0.13 | 0.06 | |
| 67 | .beta.-Phellandrene | Terpenoids | 0.05 | 0.03 | | 0.05 | 0.04 | |
| 68 | .beta.-Phenylethyl butyrate | Ester | 0.07 | 0.00 | | 0.06 | 0.01 | |
| 69 | .beta.-Phenylethylmethylethylcarbinol | Alcohol | 0.15 | 0.01 | | 0.12 | 0.02 | |
| 70 | .beta.-Pinene | Terpenoids | 0.00 | 0.00 | | 0.00 | 0.00 | |
| 71 | .beta.-Santalol | Terpenoids | 0.00 | 0.00 | | 0.00 | 0.00 | |
| 72 | .delta.-Dodecalactone | Ester | 0.00 | 0.00 | | 0.00 | 0.00 | |
| 73 | .delta.-Nonalactone | Ester | 0.02 | 0.00 | | 0.01 | 0.00 | |
| 74 | .delta.-Terpineol, acetate | Terpenoids | 2.95 | 0.22 | | 2.63 | 0.09 | |
| 75 | .gamma.-Elemene | Terpenoids | 0.07 | 0.01 | | 0.06 | 0.00 | |
| 76 | 1(2H)-Naphthalenone, octahydro-4-hydroxy-, trans- | Ketone | 0.09 | 0.01 | | 0.05 | 0.03 | |
| 77 | 1,1'-Biphenyl, 3-methyl- | Aromatics | 0.01 | 0.00 | | 0.01 | 0.00 | |
| 78 | 1,10-Undecadiene | Hydrocarbons | 0.09 | 0.00 | | 0.19 | 0.10 | |
| 79 | 1,2,4,5-Tetrazin-3-Amine | Heterocyclic compound | 0.76 | 0.04 | | 0.71 | 0.04 | |
| 80 | 1,2,4,5-Tetrazine-3,6-diAmine | Heterocyclic compound | 0.01 | 0.01 | | 0.01 | 0.00 | |
| 81 | 1,2,4-Methenoazulene, decahydro-1,5,5,8a-tetramethyl-, [1S-(1.alpha.,2.alpha.,3a.beta.,4.alpha.,8a.beta.,9R*)]- | Terpenoids | 0.00 | 0.00 | | 0.00 | 0.00 | |
| 82 | 1,2,4-Trimethoxybenzene | Aromatics | 0.15 | 0.01 | | 0.11 | 0.03 | |
| 83 | 1,2-Benzenedimethanol | Alcohol | 0.15 | 0.04 | | 0.12 | 0.01 | |
| 84 | 1,2-Benzenediol, 3-methyl- | Alcohol | 0.04 | 0.00 | | 0.04 | 0.00 | |
| 85 | 1,2-Benzenediol, 4-methyl- | Alcohol | 0.36 | 0.03 | | 0.29 | 0.01 | |
| 86 | 1,2-Cyclohexanedione | Ketone | 0.20 | 0.02 | | 0.22 | 0.03 | |
| 87 | 1,2-Ethanediol, monobenzoate | Ester | 0.02 | 0.01 | | 0.01 | 0.00 | |
| 88 | 1,2-HydrazinedicarboxAldehyde | Aldehyde | 0.09 | 0.00 | | 0.09 | 0.00 | |
| 89 | 1,2-Propanedione, 1-phenyl- | Ketone | 0.06 | 0.00 | | 0.06 | 0.01 | |
| 90 | 1,3,6-Octatriene, 3,7-dimethyl-, (Z)- | Terpenoids | 0.13 | 0.07 | | 0.13 | 0.06 | |
| 91 | 1,3,7-Octatriene, 3,7-dimethyl- | Terpenoids | 0.15 | 0.08 | | 0.09 | 0.07 | |
| 92 | 1,3-Benzodioxol-5-ol | Heterocyclic compound | 0.47 | 0.01 | | 0.43 | 0.01 | |
| 93 | 1,3-Cyclohexadiene, 5-(1,5-dimethyl-4-hexenyl)-2-methyl-, [S-(R*,S*)]- | Terpenoids | 0.02 | 0.01 | | 0.02 | 0.00 | |
| 94 | 1,3-Cyclohexadiene-1-carboxaldehyde, 2,6,6-trimethyl- | Terpenoids | 0.34 | 0.04 | | 0.33 | 0.03 | |
| 95 | 1,3-Cyclohexadiene-1-carboxylic acid, 2,6,6-trimethyl-, ethyl ester | Ester | 0.02 | 0.00 | | 0.02 | 0.00 | |
| 96 | 1,3-Cyclopentanedione, 2-acetyl-4-methyl- | Ketone | 0.16 | 0.02 | | 0.14 | 0.01 | |
| 97 | 1,3-Dioxolan-2-one | Ketone | 0.01 | 0.01 | | 0.01 | 0.01 | |
| 98 | 1,3-Dioxolane, 4-methyl-2-(2-methylpropyl)- | Heterocyclic compound | 0.07 | 0.02 | | 0.05 | 0.01 | |
| 99 | 1,3-Dioxolane-2,2-diethanol | Alcohol | 0.02 | 0.00 | | 0.01 | 0.00 | |
| 100 | 1,3-Hexadiene, 3-ethyl-2-methyl- | Hydrocarbons | 0.00 | 0.00 | | 0.00 | 0.00 | |
| 101 | 1,4-Cyclohexadiene-1-methanol, 4-(1-methylethyl)- | Alcohol | 2.05 | 0.36 | | 2.04 | 0.03 | |
| 102 | 1,4-Dithiane | Amine | 0.20 | 0.02 | | 0.15 | 0.02 | |
| 103 | 1,4-Methano-1H-indene, octahydro-4-methyl-8-methylene-7-(1-methylethyl)-, [1S-(1.alpha.,3a.beta.,4.alpha.,7.alpha.,7a.beta.)]- | Terpenoids | 0.74 | 0.10 | | 0.65 | 0.01 | |
| 104 | 1,4-Methanoazulen-3-ol, decahydro-1,5,5,8a-tetramethyl-, [1S-(1.alpha.,3.beta.,3a.beta.,4.alpha.,8a.beta.)]- | Terpenoids | 0.01 | 0.00 | | 0.01 | 0.00 | |
| 105 | 1,4-Methanoazulen-9-ol, decahydro-1,5,5,8a-tetramethyl-, [1R-(1.alpha.,3a.beta.,4.alpha.,8a.beta.,9S*)]- | Terpenoids | 0.01 | 0.00 | | 0.01 | 0.00 | |
| 106 | 1,4-Methanobenzocyclodecene, 1,2,3,4,4a,5,8,9,12,12a-decahydro- | Terpenoids | 0.01 | 0.00 | | 0.01 | 0.00 | |
| 107 | 1,4-Pentanediol | Alcohol | 0.05 | 0.06 | | 0.01 | 0.01 | |
| 108 | 1,5-Cyclohexadiene-1-methanol, 4-(1-methylethyl)- | Terpenoids | 0.19 | 0.02 | | 0.14 | 0.04 | |
| 109 | 1,5-Cyclooctadiene, 3,4-dimethyl- | Hydrocarbons | 0.94 | 0.08 | | 0.77 | 0.14 | |
| 110 | 1,5-Cyclooctadiene, 3-(1-methyl-2-propenyl)- | Hydrocarbons | 0.22 | 0.03 | | 0.20 | 0.01 | |
| 111 | 1,5-Dimethyl-1-vinyl-4-hexenyl butyrate | Ester | 0.05 | 0.00 | | 0.04 | 0.00 | |
| 112 | 1,5-Heptadien-4-one, 3,3,6-trimethyl- | Terpenoids | 0.66 | 0.12 | | 0.72 | 0.06 | |
| 113 | 1,5-Heptadiene, 2-methyl-, (Z)- | Hydrocarbons | 0.13 | 0.05 | | 0.15 | 0.03 | |
| 114 | 1,7-Nonadiene, 4,8-dimethyl- | Hydrocarbons | 0.28 | 0.19 | | 0.16 | 0.17 | |
| 115 | 1,7-Octadiene, 2,7-dimethyl-3,6-bis(methylene)- | Hydrocarbons | 0.10 | 0.06 | | 0.05 | 0.05 | |
| 116 | 1,7-Octadiene, 2-methyl-6-methylene- | Terpenoids | 0.42 | 0.01 | | 0.41 | 0.04 | |
| 117 | 1,8-Ethylenenaphthalene | Aromatics | 0.01 | 0.00 | | 0.01 | 0.00 | |
| 118 | 1-(2-Hydroxyethyl)-1,2,4-triazole | Heterocyclic compound | 0.15 | 0.04 | | 0.12 | 0.03 | |
| 119 | 1-(3-Methylbutyl)-2,3,5,6-tetramethylbenzene | Aromatics | 0.04 | 0.01 | | 0.03 | 0.00 | |
| 120 | 1-(4-methylphenyl)-Ethanone | Ketone | 1.07 | 0.33 | | 0.80 | 0.27 | |
| 121 | 1-(5-Dimethylethyl)pyrazin-2-yl-ethan-1-one | Heterocyclic compound | 0.06 | 0.00 | | 0.06 | 0.01 | |
| 122 | 1-(Furan-2-yl)-2-methylpentan-1-one | Heterocyclic compound | 0.53 | 0.03 | | 0.48 | 0.01 | |
| 123 | 1-Butanol, 3-methyl-, formate | Ester | 0.11 | 0.07 | | 0.18 | 0.02 | |
| 124 | 1-Butanone, 2-hydroxy-1-phenyl- | Ketone | 0.21 | 0.05 | | 0.17 | 0.01 | |
| 125 | 1-Cyclohexene-1-carboxAldehyde, 4-(1-methylethenyl)- | Aldehyde | 0.21 | 0.03 | | 0.20 | 0.02 | |
| 126 | 1-Cyclohexene-1-carboxAldehyde, 4-(1-methylethenyl)-, (S)- | Aldehyde | 0.05 | 0.01 | | 0.04 | 0.01 | |
| 127 | 1-Cyclohexene-1-carboxAldehyde, 5,5-dimethyl-3-oxo- | Aldehyde | 0.04 | 0.00 | | 0.04 | 0.00 | |
| 128 | 1-Cyclohexene-1-carboxaldehyde, 4-(1-methylethyl)- | Terpenoids | 0.91 | 0.10 | | 0.66 | 0.09 | |
| 129 | 1-Cyclohexene-1-carboxylic acid, 4-(1-methylethenyl)- | Terpenoids | 0.07 | 0.01 | | 0.06 | 0.02 | |
| 130 | 1-Cyclopentene-1-methanol, 2-methyl-5-(1-methylethyl)- | Alcohol | 1.01 | 0.63 | | 0.94 | 0.61 | |
| 131 | 1-Decen-3-one | Ketone | 0.03 | 0.01 | | 0.02 | 0.00 | |
| 132 | 1-Dodecanol | Alcohol | 0.03 | 0.01 | | 0.02 | 0.00 | |
| 133 | 1-Dodecene | Hydrocarbons | 0.01 | 0.00 | | 0.01 | 0.00 | |
| 134 | 1-Ethylpropyl acetate | Ester | 0.26 | 0.01 | | 0.24 | 0.02 | |
| 135 | 1-Hepten-6-one, 2-methyl- | Ketone | 1.84 | 0.06 | | 1.77 | 0.11 | |
| 136 | 1-Isopropyl-3-tert-butylbenzene | Aromatics | 0.05 | 0.00 | | 0.05 | 0.01 | |
| 137 | 1-Isopropyl-4,7-dimethyl-1,2,3,4,5,6-hexahydronaphthalene | Terpenoids | 0.01 | 0.00 | | 0.01 | 0.00 | |
| 138 | 1-Naphthalenemethanol | Alcohol | 0.00 | 0.00 | | 0.00 | 0.00 | |
| 139 | 1-OctanAmine,N-methyl- | Amine | 0.64 | 0.12 | | 0.58 | 0.07 | |
| 140 | 1-Octen-3-one | Ketone | 0.02 | 0.00 | | 0.02 | 0.00 | |
| 141 | 1-Oxaspiro[4.5]dec-6-ene, 2,6,10,10-tetramethyl- | Terpenoids | 0.22 | 0.03 | | 0.21 | 0.01 | |
| 142 | 1-Pentanone, 1-(1H-imidazol-4-yl)- | Heterocyclic compound | 0.10 | 0.01 | | 0.09 | 0.00 | |
| 143 | 1-Pentanone, 1-(4-methylphenyl)- | Ketone | 0.62 | 0.02 | | 0.56 | 0.06 | |
| 144 | 1-Pentanone, 1-(p-anisyl)-3-methyl- | Ketone | 0.04 | 0.02 | | 0.02 | 0.00 | |
| 145 | 1-PhenylcyclohexylAmine | Amine | 0.02 | 0.00 | | 0.02 | 0.00 | |
| 146 | 1-Propanone, 1-(2-furanyl)- | Heterocyclic compound | 2.29 | 0.18 | | 2.06 | 0.09 | |
| 147 | 1-Propanone, 1-(4-methoxyphenyl)- | Ketone | 0.02 | 0.00 | | 0.02 | 0.00 | |
| 148 | 1-Propanone, 1-cyclopropyl- | Ketone | 0.22 | 0.01 | | 0.20 | 0.02 | |
| 149 | 1-Tridecene | Hydrocarbons | 0.19 | 0.06 | | 0.17 | 0.02 | |
| 150 | 1-Tridecyne | Hydrocarbons | 1.97 | 1.25 | | 2.50 | 0.35 | |
| 151 | 1-Undecanol | Alcohol | 0.05 | 0.01 | | 0.03 | 0.00 | |
| 152 | 1-Undecyn-4-ol | Alcohol | 0.21 | 0.04 | | 0.15 | 0.01 | |
| 153 | 1-methyl-4-(1-methylethenyl)-1,2-Cyclohexanediol | Terpenoids | 0.03 | 0.03 | | 0.01 | 0.00 | |
| 154 | 12-Oxatetracyclo[4.3.1.1(2,5).1(4,10)]dodecane, 11-isopropylidene- | Hydrocarbons | 0.36 | 0.02 | | 0.33 | 0.01 | |
| 155 | 1H-1,2,4-Triazole, 3-chloro- | Heterocyclic compound | 0.02 | 0.00 | | 0.02 | 0.00 | |
| 156 | 1H-3a,7-Methanoazulene, 2,3,4,7,8,8a-hexahydro-3,6,8,8-tetramethyl-, [3R-(3.alpha.,3a.beta.,7.beta.,8a.alpha.)]- | Terpenoids | 0.01 | 0.00 | | 0.01 | 0.00 | |
| 157 | 1H-Benzocycloheptene, 2,4a,5,6,7,8-hexahydro-3,5,5,9-tetramethyl-, (R)- | Terpenoids | 0.02 | 0.01 | | 0.02 | 0.00 | |
| 158 | 1H-Cyclopenta[1,3]cyclopropa[1,2]benzene, octahydro-7-methyl-3-methylene-4-(1-methylethyl)-, [3aS-(3a.alpha.,3b.beta.,4.beta.,7.alpha.,7aS*)]- | Terpenoids | 0.01 | 0.01 | | 0.01 | 0.00 | |
| 159 | 1H-Cycloprop[e]azulen-7-ol, decahydro-1,1,7-trimethyl-4-methylene-, [1ar-(1a.alpha.,4a.alpha.,7.beta.,7a.beta.,7b.alpha.)]- | Terpenoids | 0.02 | 0.00 | | 0.01 | 0.00 | |
| 160 | 1H-Imidazole, 1-acetyl- | Heterocyclic compound | 3.07 | 0.64 | | 2.90 | 0.44 | |
| 161 | 1H-Imidazole, 1-methyl- | Heterocyclic compound | 0.00 | 0.00 | | 0.00 | 0.01 | |
| 162 | 1H-Imidazole, 2-propyl- | Heterocyclic compound | 0.14 | 0.01 | | 0.13 | 0.00 | |
| 163 | 1H-Imidazole-4-methanol | Heterocyclic compound | 0.25 | 0.00 | | 0.22 | 0.03 | |
| 164 | 1H-Inden-1-one, 2,3-dihydro- | Ketone | 0.01 | 0.01 | | 0.02 | 0.00 | |
| 165 | 1H-Inden-5-ol, 2,3-dihydro- | Phenol | 0.04 | 0.00 | | 0.04 | 0.00 | |
| 166 | 1H-Indene, 1,1,3-trimethyl- | Aromatics | 0.01 | 0.00 | | 0.01 | 0.00 | |
| 167 | 1H-Indene, 1-ethylidene- | Hydrocarbons | 0.01 | 0.00 | | 0.01 | 0.00 | |
| 168 | 1H-Indene, 2,3-dihydro-4-propyl- | Aromatics | 0.02 | 0.00 | | 0.01 | 0.00 | |
| 169 | 1H-Pyrazole, 1,3,5-trimethyl- | Heterocyclic compound | 0.60 | 0.38 | | 0.58 | 0.39 | |
| 170 | 1H-Pyrazole, 4,5-dihydro-5,5-dimethyl- | Heterocyclic compound | 0.06 | 0.00 | | 0.05 | 0.01 | |
| 171 | 1H-Pyrazole-1-carboximidamide, 3,5-dimethyl- | Heterocyclic compound | 1.00 | 0.29 | | 1.05 | 0.06 | |
| 172 | 1H-Pyrrole-2,5-dione, 3-ethyl-4-methyl- | Heterocyclic compound | 0.18 | 0.02 | | 0.12 | 0.00 | |
| 173 | 1H-Pyrrole-2-carbonitrile | Heterocyclic compound | 1.35 | 0.09 | | 1.23 | 0.05 | |
| 174 | 1H-Pyrrole-2-carboxAldehyde | Heterocyclic compound | 5.27 | 0.36 | | 4.72 | 0.17 | |
| 175 | 1H-Pyrrole-3-carbonitrile | Heterocyclic compound | 1.35 | 0.09 | | 1.23 | 0.05 | |
| 176 | 1H-Pyrrolo[2,3-b]pyridine, 2-(1-methylethyl)- | Heterocyclic compound | 0.85 | 0.05 | | 0.71 | 0.01 | |
| 177 | 1H-Tetrazol-5-amine | Heterocyclic compound | 0.02 | 0.01 | | 0.02 | 0.01 | |
| 178 | 1R,4R,7R,11R-1,3,4,7-Tetramethyltricyclo[5.3.1.0(4,11)]undec-2-ene | Terpenoids | 3.04 | 0.52 | | 2.41 | 0.14 | |
| 179 | 2'-Ethoxyacetophenone | Ketone | 0.78 | 0.14 | | 0.61 | 0.02 | |
| 180 | 2(1H)-Naphthalenone, 3,4,4a,5,6,7-hexahydro-1,1,4a-trimethyl- | Ketone | 0.04 | 0.01 | | 0.04 | 0.00 | |
| 181 | 2(1H)-Naphthalenone, 3,4,5,6,7,8-hexahydro- | Ketone | 38.16 | 5.70 | | 29.46 | 2.75 | |
| 182 | 2(1H)-Pyridinone | Heterocyclic compound | 0.37 | 0.03 | | 0.30 | 0.01 | |
| 183 | 2(1H)-Pyridinone, 3-acetyl-4-hydroxy-6-methyl- | Heterocyclic compound | 0.49 | 0.20 | | 0.36 | 0.05 | |
| 184 | 2(3H)-Benzofuranone, 3-methyl- | Heterocyclic compound | 0.04 | 0.02 | | 0.04 | 0.02 | |
| 185 | 2(3H)-Furanone, 5-(acetyloxy)dihydro-5-methyl- | Heterocyclic compound | 0.12 | 0.01 | | 0.09 | 0.01 | |
| 186 | 2(3H)-Furanone, 5-acetyldihydro- | Heterocyclic compound | 0.08 | 0.02 | | 0.07 | 0.01 | |
| 187 | 2(3H)-Furanone, 5-butyldihydro- | Ester | 0.06 | 0.01 | | 0.04 | 0.01 | |
| 188 | 2(3H)-Furanone, dihydro-5-propyl- | Ester | 0.07 | 0.03 | | 0.05 | 0.01 | |
| 189 | 2,2'-Ethylidenebis(5-methylfuran) | Heterocyclic compound | 0.15 | 0.03 | | 0.13 | 0.01 | |
| 190 | 2,2,6-Trimethyl-4H-1,3-dioxin-4-one | Heterocyclic compound | 0.06 | 0.01 | | 0.04 | 0.02 | |
| 191 | 2,3,5,9-tetramethyltricyclo[6.3.0.01,5]undec-3-ene | Terpenoids | 0.08 | 0.01 | | 0.09 | 0.00 | |
| 192 | 2,3,5-Trimethyl-6-ethylpyrazine | Heterocyclic compound | 1.97 | 0.22 | | 1.83 | 0.10 | |
| 193 | 2,3-Dehydro-1,8-cineole | Terpenoids | 0.24 | 0.02 | | 0.22 | 0.01 | |
| 194 | 2,3-Dimethyl-5-ethylpyrazine | Heterocyclic compound | 13.12 | 1.63 | | 13.27 | 1.90 | |
| 195 | 2,3-Pentanedione | Ketone | 0.02 | 0.01 | | 0.02 | 0.00 | |
| 196 | 2,4,6-Octatriene, 2,6-dimethyl- | Terpenoids | 0.54 | 0.03 | | 0.43 | 0.08 | |
| 197 | 2,4,6-Octatriene, 2,6-dimethyl-, (E,E)- | Terpenoids | 0.05 | 0.01 | | 0.05 | 0.02 | |
| 198 | 2,4,6-Octatriene, 2,6-dimethyl-, (E,Z)- | Terpenoids | 0.54 | 0.03 | | 0.43 | 0.08 | |
| 199 | 2,4-Cycloheptadien-1-one, 2,6,6-trimethyl- | Terpenoids | 0.34 | 0.04 | | 0.33 | 0.03 | |
| 200 | 2,4-Decadien-1-ol | Alcohol | 0.06 | 0.00 | | 0.03 | 0.03 | |
| 201 | 2,4-Decadienoic acid, ethyl ester, (E,Z)- | Ester | 0.02 | 0.01 | | 0.01 | 0.00 | |
| 202 | 2,4-Di-tert-butylphenol | Phenol | 0.01 | 0.00 | | 0.01 | 0.00 | |
| 203 | 2,4-Dioxabicyclo[3.2.0]hept-6-en-3-one, 1,5-dichloro-6,7-dimethyl- | Ketone | 0.01 | 0.00 | | 0.01 | 0.00 | |
| 204 | 2,4-Heptadien-1-ol, (E,E)- | Alcohol | 0.03 | 0.00 | | 0.02 | 0.01 | |
| 205 | 2,4-Hexadienoic acid, ethyl ester, (2E,4E)- | Ester | 0.16 | 0.00 | | 0.14 | 0.01 | |
| 206 | 2,4-Imidazolidinedione, 1-methyl- | Heterocyclic compound | 0.06 | 0.01 | | 0.05 | 0.01 | |
| 207 | 2,4-Octadienal, (E,E)- | Aldehyde | 0.30 | 0.07 | | 0.28 | 0.10 | |
| 208 | 2,4-Quinolinediol | Heterocyclic compound | 0.03 | 0.00 | | 0.03 | 0.00 | |
| 209 | 2,5-FurandicarboxAldehyde | Aldehyde | 0.11 | 0.02 | | 0.10 | 0.01 | |
| 210 | 2,5-Furandione, 3-methyl-4-propyl- | Heterocyclic compound | 0.02 | 0.00 | | 0.01 | 0.00 | |
| 211 | 2,5-Furandione, dihydro-3-methyl- | Heterocyclic compound | 0.17 | 0.19 | | 0.09 | 0.04 | |
| 212 | 2,5-ThiophenedicarboxAldehyde | Heterocyclic compound | 0.11 | 0.02 | | 0.09 | 0.02 | |
| 213 | 2,6,10-Trimethyltridecane | Hydrocarbons | 0.14 | 0.03 | | 0.11 | 0.02 | |
| 214 | 2,6,6-Trimethyl-2-cyclohexene-1,4-dione | Ketone | 0.19 | 0.01 | | 0.17 | 0.02 | |
| 215 | 2,6,6-Trimethylbicyclo[3.2.0]hept-2-en-7-one | Ketone | 2.58 | 0.49 | | 2.58 | 0.27 | |
| 216 | 2,6-Dimethyl-1-nonen-3-yn-5-ol | Alcohol | 1.31 | 0.15 | | 1.23 | 0.16 | |
| 217 | 2,6-Dimethyl-2-trans-6-octadiene | Terpenoids | 0.87 | 0.02 | | 0.81 | 0.08 | |
| 218 | 2,6-Dimethyldecane | Hydrocarbons | 0.29 | 0.02 | | 0.23 | 0.07 | |
| 219 | 2,6-Dodecadien-1-al | Aldehyde | 0.02 | 0.00 | | 0.02 | 0.00 | |
| 220 | 2,6-Nonadien-1-ol | Alcohol | 1.44 | 0.45 | | 1.22 | 0.12 | |
| 221 | 2,6-Nonadienal, (E,E)- | Aldehyde | 0.12 | 0.03 | | 0.11 | 0.01 | |
| 222 | 2,6-Nonadienal, (E,Z)- | Aldehyde | 0.12 | 0.03 | | 0.11 | 0.01 | |
| 223 | 2,6-Octadien-1-ol, 3,7-dimethyl-, acetate, (Z)- | Ester | 0.60 | 0.39 | | 0.75 | 0.03 | |
| 224 | 2,6-Octadienal, 3,7-dimethyl-, (E)- | Terpenoids | 0.02 | 0.00 | | 0.02 | 0.00 | |
| 225 | 2,6-Octadienenitrile, 3,7-dimethyl-, (Z)- | Nitrogen compounds | 0.01 | 0.01 | | 0.01 | 0.01 | |
| 226 | 2,6-Octadienoic acid, 3,7-dimethyl-, methyl ester | Ester | 0.09 | 0.02 | | 0.10 | 0.00 | |
| 227 | 2,6-Piperidinedione, 3-ethyl- | Heterocyclic compound | 0.33 | 0.03 | | 0.30 | 0.01 | |
| 228 | 2,7-Nonadien-5-one, 4,6-dimethyl- | Ketone | 0.14 | 0.02 | | 0.13 | 0.01 | |
| 229 | 2,7-Octadiene-1,6-diol, 2,6-dimethyl-, (E)- | Terpenoids | 0.04 | 0.00 | | 0.03 | 0.00 | |
| 230 | 2-((3,3-Dimethyloxiran-2-yl)methyl)-3-methylfuran | Heterocyclic compound | 0.03 | 0.00 | | 0.02 | 0.00 | |
| 231 | 2-Acetyl-1,4,5,6-tetrahydropyridine | Heterocyclic compound | 0.75 | 0.50 | | 0.43 | 0.46 | |
| 232 | 2-Acetyl-1-pyrroline | Heterocyclic compound | 0.04 | 0.03 | | 0.02 | 0.00 | |
| 233 | 2-Acetyl-3-ethylpyrazine | Heterocyclic compound | 0.12 | 0.02 | | 0.11 | 0.01 | |
| 234 | 2-Acetyl-3-methylbenzo[b]thiophene | Heterocyclic compound | 0.05 | 0.01 | | 0.04 | 0.01 | |
| 235 | 2-Acetyl-3-methylpyrazine | Heterocyclic compound | 9.07 | 1.33 | | 8.94 | 1.48 | |
| 236 | 2-Acetyl-4,4-dimethyl-cyclopent-2-enone | Ketone | 0.58 | 0.04 | | 0.59 | 0.04 | |
| 237 | 2-Acetyl-5-methylfuran | Heterocyclic compound | 2.58 | 0.12 | | 2.25 | 0.17 | |
| 238 | 2-Acetylpyrrolidine | Heterocyclic compound | 0.02 | 0.01 | | 0.02 | 0.01 | |
| 239 | 2-Acetylthiazole | Heterocyclic compound | 0.35 | 0.01 | | 0.32 | 0.03 | |
| 240 | 2-Azabicyclo[3.2.1]octan-3-one | Ketone | 0.04 | 0.00 | | 0.05 | 0.00 | |
| 241 | 2-Butanone, 4-(4-hydroxyphenyl)- | Ketone | 0.01 | 0.00 | | 0.01 | 0.00 | |
| 242 | 2-Buten-1-one, 1-(2,6,6-trimethyl-1,3-cyclohexadien-1-yl)-, (E)- | Terpenoids | 0.83 | 0.01 | | 0.77 | 0.03 | |
| 243 | 2-Buten-1-one, 1-(2,6,6-trimethyl-1-cyclohexen-1-yl)- | Ketone | 0.15 | 0.01 | | 0.13 | 0.01 | |
| 244 | 2-Butenal, 2-methyl-4-(2,6,6-trimethyl-1-cyclohexen-1-yl)- | Aldehyde | 0.01 | 0.00 | | 0.00 | 0.00 | |
| 245 | 2-Butenoic acid, 2-methyl-, ethyl ester | Ester | 0.05 | 0.07 | | 0.01 | 0.00 | |
| 246 | 2-Butenoic acid, 3-hexenyl ester, (E,Z)- | Ester | 0.12 | 0.04 | | 0.14 | 0.04 | |
| 247 | 2-Butenoic acid, ethyl ester, (E)- | Ester | 0.84 | 0.02 | | 0.81 | 0.05 | |
| 248 | 2-Butenoic acid, ethyl ester, (Z)- | Ester | 0.84 | 0.02 | | 0.81 | 0.05 | |
| 249 | 2-Butenoic acid, hexyl ester | Ester | 0.08 | 0.01 | | 0.07 | 0.00 | |
| 250 | 2-Cyclohexen-1-ol, 1-methyl-4-(1-methylethyl)-, cis- | Alcohol | 0.45 | 0.12 | | 0.58 | 0.19 | |
| 251 | 2-Cyclohexen-1-ol, 1-methyl-4-(1-methylethyl)-, trans- | Terpenoids | 0.16 | 0.02 | | 0.15 | 0.01 | |
| 252 | 2-Cyclohexen-1-ol, 2-methyl-5-(1-methylethenyl)-, acetate | Ester | 0.02 | 0.00 | | 0.01 | 0.00 | |
| 253 | 2-Cyclohexen-1-ol, 2-methyl-5-(1-methylethenyl)-, acetate, (1R-cis)- | Ester | 0.08 | 0.01 | | 0.08 | 0.02 | |
| 254 | 2-Cyclohexen-1-one | Ketone | 0.22 | 0.04 | | 0.21 | 0.01 | |
| 255 | 2-Cyclohexen-1-one, 2-hydroxy-3-methyl-6-(1-methylethyl)- | Terpenoids | 1.98 | 0.11 | | 1.71 | 0.30 | |
| 256 | 2-Cyclohexen-1-one, 3-methyl- | Ketone | 0.27 | 0.04 | | 0.26 | 0.01 | |
| 257 | 2-Cyclohexen-1-one, 3-methyl-6-(1-methylethenyl)-, (S)- | Terpenoids | 0.04 | 0.00 | | 0.03 | 0.00 | |
| 258 | 2-Cyclohexen-1-one, 3-methyl-6-(1-methylethyl)- | Terpenoids | 0.50 | 0.03 | | 0.45 | 0.01 | |
| 259 | 2-Cyclohexen-1-one, 4-hydroxy-3-methyl-6-(1-methylethyl)-, trans- | Ketone | 0.01 | 0.01 | | 0.01 | 0.00 | |
| 260 | 2-Cyclohexene-1-carboxaldehyde, 2,6,6-trimethyl- | Aldehyde | 0.06 | 0.00 | | 0.05 | 0.01 | |
| 261 | 2-Cyclopenten-1-one, 2-pentyl- | Ketone | 0.13 | 0.00 | | 0.10 | 0.02 | |
| 262 | 2-Cyclopenten-1-one, 3-methyl-2-(2-pentenyl)-, (Z)- | Ketone | 0.18 | 0.04 | | 0.18 | 0.01 | |
| 263 | 2-Cyclopentylethanol | Alcohol | 20.07 | 1.13 | | 18.07 | 1.64 | |
| 264 | 2-Ethoxy-3-methylpyrazine | Heterocyclic compound | 0.18 | 0.02 | | 0.17 | 0.02 | |
| 265 | 2-Ethyl-3-methoxypyrazine | Heterocyclic compound | 0.99 | 0.02 | | 0.67 | 0.44 | |
| 266 | 2-Ethylpiperidine | Heterocyclic compound | 3.44 | 2.23 | | 2.19 | 2.46 | |
| 267 | 2-FurancarboxAldehyde, 5-methyl- | Aldehyde | 128.98 | 5.41 | | 117.09 | 4.25 | |
| 268 | 2-Furancarboxylic acid, octyl ester | Ester | 0.00 | 0.00 | | 0.00 | 0.00 | |
| 269 | 2-Furanmethanethiol, 5-methyl- | Heterocyclic compound | 0.31 | 0.09 | | 0.34 | 0.03 | |
| 270 | 2-Furanmethanol, 5-ethenyltetrahydro-.alpha.,5-dimethyl- | Heterocyclic compound | 0.01 | 0.00 | | 0.00 | 0.00 | |
| 271 | 2-Heptanol | Alcohol | 0.42 | 0.03 | | 0.23 | 0.15 | |
| 272 | 2-Heptanone, 4,6-dimethyl- | Ketone | 0.02 | 0.01 | | 0.02 | 0.00 | |
| 273 | 2-Heptenal, 2-methyl- | Aldehyde | 0.33 | 0.06 | | 0.33 | 0.06 | |
| 274 | 2-Hexanol | Alcohol | 1.57 | 0.18 | | 1.54 | 0.14 | |
| 275 | 2-Isobutylthiazole | Heterocyclic compound | 0.12 | 0.02 | | 0.11 | 0.00 | |
| 276 | 2-Methoxy-4-vinylphenol | Aromatics | 0.13 | 0.03 | | 0.15 | 0.02 | |
| 277 | 2-Methyl-1,3-dithiacyclopentane | Heterocyclic compound | 8.47 | 0.41 | | 7.89 | 0.46 | |
| 278 | 2-Methyl-3(2-furyl)acrolein | Heterocyclic compound | 0.19 | 0.08 | | 0.19 | 0.04 | |
| 279 | 2-Methyl-3-furanthiol | Heterocyclic compound | 0.33 | 0.04 | | 0.33 | 0.02 | |
| 280 | 2-Methyl-6-(p-tolyl)hept-2-en-4-ol | Terpenoids | 0.03 | 0.00 | | 0.03 | 0.00 | |
| 281 | 2-Methyl-6-methyleneocta-2,7-dien-4-one | Terpenoids | 0.42 | 0.05 | | 0.39 | 0.07 | |
| 282 | 2-Methylbut-2-en-1-yl acetate | Alcohol | 0.01 | 0.00 | | 0.01 | 0.00 | |
| 283 | 2-Nonanol | Alcohol | 0.01 | 0.00 | | 0.01 | 0.01 | |
| 284 | 2-Nonanone | Ketone | 0.13 | 0.01 | | 0.11 | 0.01 | |
| 285 | 2-Nonen-1-ol | Alcohol | 0.93 | 0.20 | | 0.42 | 0.51 | |
| 286 | 2-Nonen-1-ol, (E)- | Alcohol | 0.57 | 0.05 | | 0.52 | 0.02 | |
| 287 | 2-Nonen-4-one | Ketone | 0.59 | 0.05 | | 0.55 | 0.07 | |
| 288 | 2-Octanol | Alcohol | 0.03 | 0.00 | | 0.03 | 0.00 | |
| 289 | 2-Octanone | Ketone | 0.60 | 0.03 | | 0.53 | 0.07 | |
| 290 | 2-Octen-1-ol, (E)- | Alcohol | 1.10 | 0.03 | | 1.04 | 0.09 | |
| 291 | 2-Octenal, 2-butyl- | Alcohol | 0.04 | 0.02 | | 0.05 | 0.00 | |
| 292 | 2-Octyl methylphosphonofluoridate | Ester | 0.08 | 0.00 | | 0.08 | 0.01 | |
| 293 | 2-Oxabicyclo[2.2.2]octan-6-ol, 1,3,3-trimethyl-, acetate | Ester | 0.22 | 0.14 | | 0.16 | 0.13 | |
| 294 | 2-Phenoxyethyl isobutyrate | Ester | 0.01 | 0.00 | | 0.01 | 0.00 | |
| 295 | 2-Piperidinimine | Heterocyclic compound | 0.16 | 0.05 | | 0.20 | 0.01 | |
| 296 | 2-Propanone, 1-(5-methyl-3H-1,2-dithiol-3-ylidene)- | Sulfur compounds | 0.02 | 0.00 | | 0.02 | 0.00 | |
| 297 | 2-Propen-1-ol, 3-phenyl- | Alcohol | 0.16 | 0.00 | | 0.15 | 0.00 | |
| 298 | 2-Propen-1-ol, 3-phenyl-, (E)- | Alcohol | 0.16 | 0.00 | | 0.15 | 0.00 | |
| 299 | 2-Propenal, 2-methyl-3-phenyl- | Aldehyde | 0.10 | 0.02 | | 0.09 | 0.00 | |
| 300 | 2-Propenoic Acid, 3-phenyl- | Acid | 0.10 | 0.02 | | 0.05 | 0.00 | |
| 301 | 2-Propenoic acid, 2-methyl-, (tetrahydro-2-furanyl)methyl ester | Ester | 0.12 | 0.01 | | 0.11 | 0.01 | |
| 302 | 2-Propenoic acid, 2-methyl-, 3,3,5-trimethylcyclohexyl ester | Ester | 0.14 | 0.02 | | 0.11 | 0.01 | |
| 303 | 2-Propenoic acid, 3-phenyl-, methyl ester | Ester | 0.01 | 0.01 | | 0.01 | 0.00 | |
| 304 | 2-Propenoic acid, 3-phenyl-, methyl ester, (E)- | Ester | 0.23 | 0.03 | | 0.21 | 0.01 | |
| 305 | 2-Propenoic acid, pentyl ester | Ester | 0.04 | 0.01 | | 0.05 | 0.00 | |
| 306 | 2-Pyridinecarbonitrile | Heterocyclic compound | 0.05 | 0.01 | | 0.04 | 0.00 | |
| 307 | 2-PyrrolidinemethanAmine, N-methyl-, (S)- | Heterocyclic compound | 0.56 | 0.06 | | 0.49 | 0.02 | |
| 308 | 2-Pyrrolidinone, 1-methyl- | Heterocyclic compound | 0.13 | 0.03 | | 0.09 | 0.06 | |
| 309 | 2-Tetradecene, (E)- | Hydrocarbons | 0.02 | 0.00 | | 0.02 | 0.00 | |
| 310 | 2-Thiophenemethanethiol | Heterocyclic compound | 1.51 | 0.15 | | 1.19 | 0.15 | |
| 311 | 2-Tridecanone | Ketone | 0.01 | 0.00 | | 0.01 | 0.00 | |
| 312 | 2-Undecanone | Ketone | 0.08 | 0.01 | | 0.08 | 0.01 | |
| 313 | 2-Undecanone, 6,10-dimethyl- | Ketone | 0.01 | 0.00 | | 0.01 | 0.00 | |
| 314 | 2-hydroxy-BenzAldehyde | Aldehyde | 0.10 | 0.05 | | 0.07 | 0.00 | |
| 315 | 2-methoxy-Phenol | Phenol | 3.60 | 0.06 | | 3.19 | 0.32 | |
| 316 | 2-methyl-5-(1-methylethenyl)-Cyclohexanol | Terpenoids | 0.14 | 0.03 | | 0.16 | 0.04 | |
| 317 | 2-methyl-5-propan-2-ylcyclohexa-2,5-diene-1,4-dione | Ketone | 0.04 | 0.01 | | 0.03 | 0.00 | |
| 318 | 2-methylprop-2-enoyl 2-methylprop-2-enoate | Others | 0.20 | 0.00 | | 0.18 | 0.01 | |
| 319 | 2H-1-Benzopyran-2-one, 3-methyl- | Heterocyclic compound | 0.01 | 0.00 | | 0.01 | 0.00 | |
| 320 | 2H-Indol-2-one, 1,3-dihydro- | Heterocyclic compound | 0.02 | 0.00 | | 0.02 | 0.00 | |
| 321 | 2H-Pyran, 3,6-dihydro-4-methyl-2-(2-methyl-1-propenyl)- | Terpenoids | 0.16 | 0.02 | | 0.15 | 0.00 | |
| 322 | 2H-Pyran-2-one, 6-hexyltetrahydro- | Ester | 0.00 | 0.00 | | 0.00 | 0.00 | |
| 323 | 2H-Pyran-2-one, 6-pentyl- | Heterocyclic compound | 0.02 | 0.02 | | 0.04 | 0.01 | |
| 324 | 2H-Pyran-2-one, tetrahydro- | Heterocyclic compound | 3.20 | 0.09 | | 2.69 | 0.35 | |
| 325 | 2H-Pyran-2-one, tetrahydro-4,6,6-trimethyl- | Heterocyclic compound | 0.74 | 0.02 | | 0.38 | 0.23 | |
| 326 | 2H-Pyran-2-one, tetrahydro-6-pentyl- | Ester | 0.03 | 0.00 | | 0.02 | 0.00 | |
| 327 | 2H-Pyran-2-one, tetrahydro-6-propyl- | Heterocyclic compound | 0.04 | 0.00 | | 0.04 | 0.00 | |
| 328 | 2H-Pyran-3-ol, tetrahydro-2,2,6-trimethyl-6-(4-methyl-3-cyclohexen-1-yl)-, [3S-[3.alpha.,6.alpha.(R*)]]- | Terpenoids | 0.00 | 0.00 | | 0.00 | 0.00 | |
| 329 | 3(2H)-Furanone, 2-hexyl-5-methyl- | Heterocyclic compound | 0.01 | 0.00 | | 0.01 | 0.00 | |
| 330 | 3(2H)-Furanone, 4-methoxy-2,5-dimethyl- | Heterocyclic compound | 0.01 | 0.00 | | 0.01 | 0.00 | |
| 331 | 3(2H)-Pyridazinone | Heterocyclic compound | 1.20 | 0.17 | | 1.07 | 0.07 | |
| 332 | 3(2H)-Thiophenone, dihydro-2-methyl- | Heterocyclic compound | 0.65 | 0.03 | | 0.65 | 0.08 | |
| 333 | 3,4-Dimethoxytoluene | Aromatics | 0.10 | 0.02 | | 0.08 | 0.01 | |
| 334 | 3,4-Hexanedione | Ketone | 1.69 | 1.24 | | 0.33 | 0.01 | |
| 335 | 3,5-Dimethyldodecane | Hydrocarbons | 0.05 | 0.01 | | 0.04 | 0.01 | |
| 336 | 3,5-Octadien-2-one | Ketone | 0.81 | 0.17 | | 0.63 | 0.03 | |
| 337 | 3,5-Octadien-2-one, (E,E)- | Ketone | 0.81 | 0.17 | | 0.63 | 0.03 | |
| 338 | 3,6-Dimethyl-2,3,3a,4,5,7a-hexahydrobenzofuran | Heterocyclic compound | 0.00 | 0.00 | | 0.00 | 0.00 | |
| 339 | 3,6-Dimethyl-4H-furo[3,2-c]pyran-4-one | Heterocyclic compound | 0.23 | 0.03 | | 0.18 | 0.02 | |
| 340 | 3,6-Nonadien-1-ol, (E,Z)- | Alcohol | 0.06 | 0.03 | | 0.05 | 0.00 | |
| 341 | 3-(4-Hydroxyphenyl)propanal | Aldehyde | 0.07 | 0.02 | | 0.05 | 0.01 | |
| 342 | 3-(Benzo(b)thien-6-yl)propionic Acid | Heterocyclic compound | 0.02 | 0.00 | | 0.02 | 0.00 | |
| 343 | 3-Acetyl-2,5-dimethyl furan | Heterocyclic compound | 0.20 | 0.00 | | 0.19 | 0.01 | |
| 344 | 3-Buten-2-ol, 4-(2,6,6-trimethyl-2-cyclohexen-1-yl)-, (3E)- | Alcohol | 0.02 | 0.00 | | 0.02 | 0.01 | |
| 345 | 3-Buten-2-one, 4-(2,2,6-trimethyl-7-oxabicyclo[4.1.0]hept-1-yl)- | Terpenoids | 0.01 | 0.01 | | 0.01 | 0.00 | |
| 346 | 3-Butylthiophene | Heterocyclic compound | 1.40 | 0.05 | | 1.34 | 0.07 | |
| 347 | 3-Carene | Terpenoids | 0.36 | 0.02 | | 0.23 | 0.15 | |
| 348 | 3-Cyclohexen-1-ol, 1-methyl-4-(1-methylethyl)- | Terpenoids | 0.11 | 0.07 | | 0.13 | 0.01 | |
| 349 | 3-Cyclohexen-1-ol, 4-methyl-1-(1-methylethyl)-, (R)- | Terpenoids | 0.04 | 0.03 | | 0.02 | 0.00 | |
| 350 | 3-Cyclohexene-1-acetAldehyde, .alpha.,4-dimethyl- | Aldehyde | 0.34 | 0.23 | | 0.28 | 0.17 | |
| 351 | 3-Cyclohexene-1-ethanol, .beta.,4-dimethyl- | Alcohol | 0.07 | 0.01 | | 0.06 | 0.01 | |
| 352 | 3-Cyclohexene-1-methanethiol, .alpha.,.alpha.,4-trimethyl- | Sulfur compounds | 0.38 | 0.14 | | 0.30 | 0.12 | |
| 353 | 3-Cyclohexene-1-methanol, .alpha.,.alpha.,4-trimethyl-, propanoate | Terpenoids | 0.03 | 0.01 | | 0.02 | 0.00 | |
| 354 | 3-Cyclopentyl-1-propanol | Alcohol | 0.11 | 0.00 | | 0.10 | 0.01 | |
| 355 | 3-FurAldehyde | Heterocyclic compound | 100.16 | 16.92 | | 86.50 | 3.55 | |
| 356 | 3-Heptanone, 2,6-dimethyl- | Ketone | 0.16 | 0.01 | | 0.14 | 0.02 | |
| 357 | 3-Hexen-1-ol, acetate, (E)- | Ester | 0.14 | 0.01 | | 0.13 | 0.01 | |
| 358 | 3-Hexen-1-ol, acetate, (Z)- | Ester | 0.14 | 0.01 | | 0.13 | 0.01 | |
| 359 | 3-Hexen-1-ol, formate, (Z)- | Ester | 0.40 | 0.01 | | 0.31 | 0.05 | |
| 360 | 3-Hexen-1-ol, propanoate, (Z)- | Ester | 0.07 | 0.01 | | 0.06 | 0.01 | |
| 361 | 3-Hexenal, (Z)- | Aldehyde | 0.05 | 0.00 | | 0.05 | 0.00 | |
| 362 | 3-Hexene, 1-(1-ethoxyethoxy)-, (Z)- | Aldehyde | 0.02 | 0.00 | | 0.02 | 0.00 | |
| 363 | 3-Isopropylidene-5-methyl-hex-4-en-2-one | Ketone | 0.66 | 0.05 | | 0.59 | 0.04 | |
| 364 | 3-Mercaptohexanol | Alcohol | 0.84 | 0.09 | | 0.70 | 0.09 | |
| 365 | 3-Mercaptohexyl acetate | Ester | 0.11 | 0.00 | | 0.09 | 0.01 | |
| 366 | 3-Methoxy-2,5-dimethylpyrazine | Heterocyclic compound | 0.99 | 0.02 | | 0.67 | 0.44 | |
| 367 | 3-Methoxy-5-methylphenol | Phenol | 0.04 | 0.00 | | 0.03 | 0.01 | |
| 368 | 3-Methoxyphenyl acetone | Ketone | 0.04 | 0.00 | | 0.03 | 0.00 | |
| 369 | 3-Methyl-4,5-diamino-1,2,4(4H)-triazole | Heterocyclic compound | 0.04 | 0.00 | | 0.03 | 0.00 | |
| 370 | 3-Methylbenzothiophene | Heterocyclic compound | 0.16 | 0.01 | | 0.14 | 0.00 | |
| 371 | 3-Methylheptyl acetate | Ester | 0.27 | 0.02 | | 0.25 | 0.04 | |
| 372 | 3-Methylpenta-1,3-diene-5-ol, (E)- | Alcohol | 4.98 | 0.26 | | 5.30 | 0.71 | |
| 373 | 3-Nonen-1-ol, (E)- | Alcohol | 0.19 | 0.03 | | 0.15 | 0.01 | |
| 374 | 3-Octanol | Alcohol | 0.14 | 0.00 | | 0.13 | 0.01 | |
| 375 | 3-Octanone | Ketone | 0.60 | 0.03 | | 0.53 | 0.07 | |
| 376 | 3-Octen-2-one | Ketone | 6.21 | 4.75 | | 9.13 | 0.47 | |
| 377 | 3-Octen-2-one, (E)- | Ketone | 0.17 | 0.11 | | 0.09 | 0.09 | |
| 378 | 3-Octen-2-one, 7-methyl- | Ketone | 0.09 | 0.01 | | 0.08 | 0.02 | |
| 379 | 3-Oxatricyclo[4.1.1.0(2,4)]octane, 2,7,7-trimethyl- | Terpenoids | 0.21 | 0.07 | | 0.16 | 0.03 | |
| 380 | 3-Penten-2-one, 4-(acetyloxy)-, (Z)- | Ketone | 0.05 | 0.00 | | 0.04 | 0.00 | |
| 381 | 3-Penten-2-one, 4-methyl- | Ketone | 0.05 | 0.01 | | 0.04 | 0.01 | |
| 382 | 3-Phenyl-1-propanol, acetate | Ester | 0.03 | 0.00 | | 0.02 | 0.01 | |
| 383 | 3-Phenylpropanol | Alcohol | 0.01 | 0.00 | | 0.01 | 0.00 | |
| 384 | 3-n-Propyl-2-pyrazolin-5-one | Heterocyclic compound | 0.06 | 0.01 | | 0.06 | 0.00 | |
| 385 | 3H-3a,7-Methanoazulene, 2,4,5,6,7,8-hexahydro-1,4,9,9-tetramethyl-, [3aR-(3a.alpha.,4.beta.,7.alpha.)]- | Terpenoids | 0.01 | 0.00 | | 0.01 | 0.00 | |
| 386 | 4-(Aminomethyl)pyridine | Heterocyclic compound | 1.71 | 0.11 | | 1.45 | 0.19 | |
| 387 | 4-Aminobenzoic acid | Acid | 0.18 | 0.01 | | 0.15 | 0.01 | |
| 388 | 4-Decenal, (E)- | Aldehyde | 0.06 | 0.00 | | 0.05 | 0.01 | |
| 389 | 4-Decenoic acid, methyl ester, Z- | Ester | 0.71 | 0.07 | | 0.62 | 0.01 | |
| 390 | 4-Heptanone, oxime | Amine | 0.01 | 0.00 | | 0.01 | 0.00 | |
| 391 | 4-Heptenal | Aldehyde | 1.28 | 0.04 | | 1.20 | 0.04 | |
| 392 | 4-Heptenal, (Z)- | Aldehyde | 1.09 | 0.06 | | 1.05 | 0.07 | |
| 393 | 4-Hexen-1-ol, 5-methyl-2-(1-methylethenyl)-, (R)- | Terpenoids | 0.09 | 0.01 | | 0.08 | 0.02 | |
| 394 | 4-Hexen-3-one, 5-methyl- | Ketone | 0.07 | 0.00 | | 0.06 | 0.01 | |
| 395 | 4-Hydroxy-3-methoxybenzyl Alcohol | Alcohol | 0.01 | 0.00 | | 0.01 | 0.00 | |
| 396 | 4-Isopropyl-1,3-cyclohexanedione | Ketone | 0.07 | 0.01 | | 0.06 | 0.01 | |
| 397 | 4-Isopropylcyclohexa-1,3-dienecarbAldehyde | Aldehyde | 0.20 | 0.04 | | 0.16 | 0.02 | |
| 398 | 4-MethoxycinnamAldehyde | Aldehyde | 0.21 | 0.04 | | 0.17 | 0.06 | |
| 399 | 4-Methyl-5-nonanone | Ketone | 0.28 | 0.11 | | 0.28 | 0.06 | |
| 400 | 4-Methylpentyl 2-methylbutanoate | Ester | 0.47 | 0.04 | | 0.54 | 0.07 | |
| 401 | 4-Methylthiazole | Heterocyclic compound | 1.13 | 0.10 | | 1.04 | 0.11 | |
| 402 | 4-Nonenal, (E)- | Aldehyde | 0.11 | 0.07 | | 0.13 | 0.01 | |
| 403 | 4-Penten-1-ol, propanoate | Ester | 0.06 | 0.01 | | 0.06 | 0.01 | |
| 404 | 4-Phenyl-2-butanol | Alcohol | 0.54 | 0.03 | | 0.47 | 0.01 | |
| 405 | 4-Pyridinecarboxylic acid, ethyl ester | Heterocyclic compound | 0.14 | 0.01 | | 0.12 | 0.01 | |
| 406 | 4-Undecanone | Ketone | 0.06 | 0.00 | | 0.06 | 0.00 | |
| 407 | 4-tert-Butylcyclohexyl acetate | Ester | 0.03 | 0.01 | | 0.02 | 0.00 | |
| 408 | 4a(2H)-Naphthalenol, 1,3,4,5,6,8a-hexahydro-4,7-dimethyl-1-(1-methylethyl)-, (1S,4S,4aS,8aR)- | Terpenoids | 0.00 | 0.00 | | 0.00 | 0.00 | |
| 409 | 5,6,7,8-Tetrahydroquinoxaline | Heterocyclic compound | 0.06 | 0.01 | | 0.06 | 0.01 | |
| 410 | 5,7-Octadien-4-one, 2,6-dimethyl-, (E)- | Terpenoids | 0.10 | 0.01 | | 0.09 | 0.02 | |
| 411 | 5,7-Octadien-4-one, 2,6-dimethyl-, (Z)- | Terpenoids | 1.08 | 0.08 | | 0.96 | 0.02 | |
| 412 | 5,8-Decadien-2-one, 5,9-dimethyl-, (E)- | Ketone | 8.67 | 0.56 | | 7.42 | 0.13 | |
| 413 | 5,9-Undecadien-2-ol, 6,10-dimethyl- | Alcohol | 0.01 | 0.00 | | 0.01 | 0.00 | |
| 414 | 5,9-Undecadien-2-one, 6,10-dimethyl- | Terpenoids | 0.02 | 0.00 | | 0.01 | 0.00 | |
| 415 | 5,9-Undecadien-2-one, 6,10-dimethyl-, (E)- | Ketone | 0.02 | 0.00 | | 0.01 | 0.00 | |
| 416 | 5-Acetyl-2,4-dimethylthiazole | Heterocyclic compound | 0.09 | 0.00 | | 0.07 | 0.01 | |
| 417 | 5-Azulenemethanol, 1,2,3,4,5,6,7,8-octahydro-.alpha.,.alpha.,3,8-tetramethyl-, acetate, [3S-(3.alpha.,5.alpha.,8.alpha.)]- | Ester | 0.01 | 0.00 | | 0.00 | 0.00 | |
| 418 | 5-Ethylhydantoin | Heterocyclic compound | 0.05 | 0.00 | | 0.05 | 0.01 | |
| 419 | 5-Hepten-2-ol, 6-methyl- | Alcohol | 1.63 | 0.05 | | 1.54 | 0.12 | |
| 420 | 5-Isoxazolecarboxylic acid, 4,5-dihydro-5-methyl-, methyl ester, (R)- | Heterocyclic compound | 0.13 | 0.04 | | 0.07 | 0.04 | |
| 421 | 5-Methyl-(E)-2-hepten-4-one | Ketone | 9.72 | 0.38 | | 9.06 | 0.37 | |
| 422 | 5-Methyl-2-thiophenecarboxaldehyde | Heterocyclic compound | 0.42 | 0.06 | | 0.31 | 0.13 | |
| 423 | 5-Methyloxazolidine | Heterocyclic compound | 0.43 | 0.05 | | 0.36 | 0.01 | |
| 424 | 5-Pyrimidinamine, 2-chloro-4-ethoxy- | Heterocyclic compound | 0.10 | 0.01 | | 0.08 | 0.00 | |
| 425 | 5-propan-2-ylbicyclo[3.1.0]hex-2-ene-2-carbAldehyde | Aldehyde | 0.27 | 0.15 | | 0.17 | 0.01 | |
| 426 | 5H-5-Methyl-6,7-dihydrocyclopentapyrazine | Heterocyclic compound | 1.84 | 0.16 | | 1.87 | 0.10 | |
| 427 | 6,7-Dimethyl-1,2,3,5,8,8a-hexahydronaphthalene | Aromatics | 0.29 | 0.05 | | 0.21 | 0.02 | |
| 428 | 6-Ethyl-5,6-dihydro-2H-pyran-2-one | Heterocyclic compound | 0.12 | 0.04 | | 0.10 | 0.04 | |
| 429 | 6-Methyl-2-(4-methylcyclohex-3-en-1-yl)hepta-1,5-dien-4-ol | Terpenoids | 0.01 | 0.00 | | 0.00 | 0.00 | |
| 430 | 6-Methyl-6-(5-methylfuran-2-yl)heptan-2-one | Heterocyclic compound | 0.02 | 0.01 | | 0.02 | 0.00 | |
| 431 | 6-Nonen-1-ol, (E)- | Alcohol | 0.16 | 0.00 | | 0.15 | 0.00 | |
| 432 | 6-Nonenal, (E)- | Aldehyde | 0.16 | 0.03 | | 0.12 | 0.03 | |
| 433 | 6-Nonenal, (Z)- | Aldehyde | 0.11 | 0.07 | | 0.16 | 0.01 | |
| 434 | 6-Octen-1-ol, 3,7-dimethyl-, acetate | Ester | 0.03 | 0.02 | | 0.04 | 0.00 | |
| 435 | 6-Octen-1-ol, 3,7-dimethyl-, formate | Terpenoids | 1.21 | 0.09 | | 1.17 | 0.03 | |
| 436 | 6-Octen-1-ol, 7-methyl-3-methylene- | Terpenoids | 0.16 | 0.01 | | 0.14 | 0.00 | |
| 437 | 6-Octenal, 7-methyl-3-methylene- | Aldehyde | 0.05 | 0.01 | | 0.03 | 0.00 | |
| 438 | 7-Octen-4-ol, 2-methyl-6-methylene-, (S)- | Terpenoids | 0.06 | 0.02 | | 0.06 | 0.01 | |
| 439 | 8-Methyl-6-nonenoic Acid | Acid | 0.00 | 0.00 | | 0.00 | 0.00 | |
| 440 | AcetAldehyde, (3,3-dimethylcyclohexylidene)-, (Z)- | Aldehyde | 0.20 | 0.01 | | 0.18 | 0.01 | |
| 441 | Acetic Acid, [(1-thioxoethyl)thio]- | Acid | 0.03 | 0.00 | | 0.02 | 0.00 | |
| 442 | Acetic Acid, phenoxy- | Acid | 0.13 | 0.02 | | 0.13 | 0.01 | |
| 443 | Acetic acid, (propylthio)-, methyl ester | Ester | 0.04 | 0.00 | | 0.03 | 0.00 | |
| 444 | Acetic acid, 2-ethylhexyl ester | Ester | 0.15 | 0.02 | | 0.14 | 0.00 | |
| 445 | Acetic acid, 2-phenylethyl ester | Ester | 0.02 | 0.00 | | 0.02 | 0.01 | |
| 446 | Acetic acid, cyclohexyl ester | Ester | 0.53 | 0.02 | | 0.44 | 0.04 | |
| 447 | Acetic acid, nonyl ester | Ester | 0.07 | 0.05 | | 0.08 | 0.01 | |
| 448 | Acetic acid, phenyl ester | Ester | 13.32 | 0.52 | | 11.91 | 1.45 | |
| 449 | Acetoacetic acid isoamyl ester | Ester | 0.01 | 0.00 | | 0.00 | 0.00 | |
| 450 | Acetophenone | Ketone | 0.19 | 0.00 | | 0.17 | 0.01 | |
| 451 | Anabasine | Heterocyclic compound | 0.01 | 0.00 | | 0.01 | 0.00 | |
| 452 | Anethole | Aromatics | 0.21 | 0.01 | | 0.19 | 0.01 | |
| 453 | Apocynin | Ketone | 0.04 | 0.01 | | 0.02 | 0.00 | |
| 454 | Azacyclohexan-3-one, 1,5,6-trimethyl- | Heterocyclic compound | 0.24 | 0.02 | | 0.21 | 0.02 | |
| 455 | BenzAldehyde | Aldehyde | 1.25 | 0.08 | | 1.08 | 0.08 | |
| 456 | BenzAldehyde diethylacetal | Aldehyde | 0.17 | 0.21 | | 0.15 | 0.10 | |
| 457 | BenzAldehyde, 2,5-dimethyl- | Aldehyde | 0.40 | 0.05 | | 0.31 | 0.03 | |
| 458 | BenzAldehyde, 3,4-dimethoxy- | Aldehyde | 0.03 | 0.01 | | 0.02 | 0.00 | |
| 459 | BenzAldehyde, 3-hydroxy- | Aldehyde | 0.29 | 0.10 | | 0.30 | 0.05 | |
| 460 | BenzAldehyde, 4-hydroxy- | Aldehyde | 0.21 | 0.09 | | 0.25 | 0.02 | |
| 461 | BenzAldehyde, 4-methoxy- | Aldehyde | 0.08 | 0.01 | | 0.08 | 0.01 | |
| 462 | BenzAldehyde, 4-methyl- | Aldehyde | 0.07 | 0.07 | | 0.06 | 0.07 | |
| 463 | Benzene, (1-methoxypropyl)- | Aromatics | 0.51 | 0.08 | | 0.32 | 0.03 | |
| 464 | Benzene, (1-methylpropyl)- | Aromatics | 0.20 | 0.00 | | 0.19 | 0.00 | |
| 465 | Benzene, (1-nitropropyl)- | Aromatics | 1.03 | 0.02 | | 0.91 | 0.03 | |
| 466 | Benzene, (2,2-dimethoxyethyl)- | Aromatics | 0.01 | 0.01 | | 0.01 | 0.01 | |
| 467 | Benzene, (2-methoxyethyl)- | Aromatics | 0.07 | 0.04 | | 0.08 | 0.04 | |
| 468 | Benzene, (2-methyl-1-propenyl)- | Aromatics | 0.01 | 0.00 | | 0.01 | 0.00 | |
| 469 | Benzene, (2-nitroethyl)- | Aromatics | 0.03 | 0.00 | | 0.03 | 0.00 | |
| 470 | Benzene, (butoxymethyl)- | Ether | 0.34 | 0.04 | | 0.33 | 0.01 | |
| 471 | Benzene, (isothiocyanatomethyl)- | Sulfur compounds | 0.13 | 0.00 | | 0.12 | 0.01 | |
| 472 | Benzene, (methoxymethyl)- | Ether | 0.91 | 0.03 | | 0.83 | 0.02 | |
| 473 | Benzene, (methylthio)- | Sulfur compounds | 0.11 | 0.01 | | 0.09 | 0.00 | |
| 474 | Benzene, 1,2,3,5-tetramethyl- | Aromatics | 1.31 | 0.20 | | 1.13 | 0.15 | |
| 475 | Benzene, 1,2,3-trimethyl- | Aromatics | 0.05 | 0.01 | | 0.04 | 0.01 | |
| 476 | Benzene, 1,2,4,5-tetramethyl- | Aromatics | 1.31 | 0.20 | | 1.13 | 0.15 | |
| 477 | Benzene, 1,2-(methylenedioxy)-4-propenyl-, (E)- | Aromatics | 0.02 | 0.00 | | 0.02 | 0.00 | |
| 478 | Benzene, 1,2-diethyl-3,4,5,6-tetramethyl- | Aromatics | 0.02 | 0.00 | | 0.02 | 0.00 | |
| 479 | Benzene, 1,2-dinitro- | Aromatics | 0.15 | 0.01 | | 0.13 | 0.01 | |
| 480 | Benzene, 1,3,5-triethyl- | Aromatics | 0.28 | 0.01 | | 0.25 | 0.03 | |
| 481 | Benzene, 1,3-bis(1-methylethyl)- | Aromatics | 0.02 | 0.00 | | 0.02 | 0.00 | |
| 482 | Benzene, 1,3-dimethyl- | Aromatics | 0.11 | 0.02 | | 0.10 | 0.03 | |
| 483 | Benzene, 1-(1,5-dimethyl-4-hexenyl)-4-methyl- | Terpenoids | 0.02 | 0.00 | | 0.01 | 0.01 | |
| 484 | Benzene, 1-(1,5-dimethylhexyl)-4-methyl- | Terpenoids | 0.04 | 0.01 | | 0.03 | 0.01 | |
| 485 | Benzene, 1-ethenyl-3,5-dimethyl- | Aromatics | 0.19 | 0.00 | | 0.17 | 0.01 | |
| 486 | Benzene, 1-ethyl-3,5-dimethyl- | Aromatics | 0.51 | 0.14 | | 0.47 | 0.06 | |
| 487 | Benzene, 1-ethyl-3-methyl- | Aromatics | 1.13 | 0.09 | | 1.00 | 0.11 | |
| 488 | Benzene, 1-ethyl-4-methoxy- | Aromatics | 2.78 | 1.56 | | 1.85 | 0.44 | |
| 489 | Benzene, 1-methoxy-2-nitro- | Aromatics | 0.04 | 0.01 | | 0.04 | 0.00 | |
| 490 | Benzene, 1-methoxy-4-(1-methylpropyl)- | Ether | 0.26 | 0.02 | | 0.24 | 0.01 | |
| 491 | Benzene, 1-methoxy-4-(1-propenyl)-, (Z)- | Ether | 0.17 | 0.01 | | 0.15 | 0.02 | |
| 492 | Benzene, 1-methoxy-4-methyl-2-(1-methylethyl)- | Aromatics | 0.05 | 0.01 | | 0.04 | 0.00 | |
| 493 | Benzene, 1-methoxy-4-nitro- | Aromatics | 0.06 | 0.01 | | 0.06 | 0.01 | |
| 494 | Benzene, 1-methyl-3-(1-methylethenyl)- | Aromatics | 0.01 | 0.00 | | 0.01 | 0.00 | |
| 495 | Benzene, 1-methyl-4-(1,2,2-trimethylcyclopentyl)-, (R)- | Terpenoids | 0.01 | 0.00 | | 0.01 | 0.00 | |
| 496 | Benzene, 1-propynyl- | Aromatics | 0.01 | 0.00 | | 0.01 | 0.00 | |
| 497 | Benzene, 2,4-pentadiynyl- | Aromatics | 1.48 | 0.35 | | 1.76 | 0.12 | |
| 498 | Benzene, 2-methyl-1,4-bis(1-methylethyl)- | Aromatics | 0.05 | 0.01 | | 0.04 | 0.01 | |
| 499 | Benzene, 4-ethenyl-1,2-dimethyl- | Aromatics | 0.19 | 0.00 | | 0.17 | 0.01 | |
| 500 | Benzene, nitro- | Aromatics | 0.10 | 0.07 | | 0.08 | 0.05 | |
| 501 | BenzeneacetAldehyde | Aldehyde | 5.41 | 0.38 | | 5.01 | 0.28 | |
| 502 | BenzeneacetAldehyde, .alpha.-(2-methylpropylidene)- | Aldehyde | 0.87 | 0.07 | | 0.80 | 0.07 | |
| 503 | BenzeneacetAldehyde, .alpha.-ethylidene- | Aldehyde | 0.51 | 0.03 | | 0.54 | 0.04 | |
| 504 | Benzeneacetic acid | Acid | 0.17 | 0.11 | | 0.21 | 0.01 | |
| 505 | Benzeneacetic acid, 2-methylpropyl ester | Ester | 0.09 | 0.00 | | 0.08 | 0.01 | |
| 506 | Benzeneacetic acid, ethyl ester | Ester | 0.13 | 0.10 | | 0.03 | 0.03 | |
| 507 | BenzeneethanAmine, N,.alpha.,.alpha.-trimethyl- | Amine | 0.01 | 0.00 | | 0.01 | 0.00 | |
| 508 | Benzeneethanol, 4-hydroxy- | Alcohol | 0.03 | 0.00 | | 0.03 | 0.00 | |
| 509 | Benzenemethanol, .alpha.,.alpha.,4-trimethyl- | Terpenoids | 0.10 | 0.04 | | 0.11 | 0.03 | |
| 510 | Benzenemethanol, .alpha.,4-dimethyl- | Alcohol | 0.45 | 0.03 | | 0.23 | 0.12 | |
| 511 | Benzenemethanol, .alpha.-2-propenyl- | Alcohol | 0.21 | 0.02 | | 0.16 | 0.09 | |
| 512 | Benzenemethanol, .alpha.-methyl- | Alcohol | 0.15 | 0.12 | | 0.19 | 0.10 | |
| 513 | Benzenemethanol, 3-hydroxy- | Alcohol | 0.32 | 0.02 | | 0.27 | 0.04 | |
| 514 | Benzenemethanol, 4-methoxy- | Phenol | 1.11 | 0.08 | | 0.83 | 0.11 | |
| 515 | Benzenemethanol, 4-methyl- | Alcohol | 0.19 | 0.01 | | 0.17 | 0.02 | |
| 516 | Benzenepropanoic acid, ethyl ester | Ester | 0.20 | 0.02 | | 0.17 | 0.01 | |
| 517 | Benzenepropanol, 4-hydroxy-3-methoxy- | Alcohol | 0.01 | 0.00 | | 0.01 | 0.00 | |
| 518 | Benzofuran | Heterocyclic compound | 0.24 | 0.03 | | 0.21 | 0.01 | |
| 519 | Benzofuran, 4,5,6,7-tetrahydro-3,6-dimethyl- | Terpenoids | 1.66 | 0.16 | | 1.53 | 0.11 | |
| 520 | Benzoic acid | Acid | 0.18 | 0.12 | | 0.19 | 0.02 | |
| 521 | Benzoic acid, 1-methylethyl ester | Ester | 0.06 | 0.00 | | 0.04 | 0.01 | |
| 522 | Benzoic acid, 2-(dimethylamino)-, methyl ester | Ester | 0.02 | 0.00 | | 0.01 | 0.00 | |
| 523 | Benzoic acid, 2-(methylamino)-, methyl ester | Ester | 0.07 | 0.01 | | 0.06 | 0.01 | |
| 524 | Benzoic acid, 2-hydroxy-, ethyl ester | Ester | 0.06 | 0.01 | | 0.05 | 0.00 | |
| 525 | Benzoic acid, 2-methylpropyl ester | Ester | 0.43 | 0.02 | | 0.38 | 0.01 | |
| 526 | Benzoic acid, 2-propenyl ester | Ester | 0.01 | 0.01 | | 0.02 | 0.00 | |
| 527 | Benzoic acid, 4-amino-, methyl ester | Ester | 0.14 | 0.01 | | 0.12 | 0.00 | |
| 528 | Benzoic acid, ethyl ester | Ester | 0.40 | 0.05 | | 0.24 | 0.09 | |
| 529 | Benzoic acid, hexyl ester | Ester | 0.03 | 0.00 | | 0.02 | 0.00 | |
| 530 | Benzophenone | Ketone | 0.02 | 0.00 | | 0.02 | 0.00 | |
| 531 | Benzothiazole | Heterocyclic compound | 0.26 | 0.08 | | 0.27 | 0.02 | |
| 532 | Benzoxazole, 2-methyl- | Heterocyclic compound | 0.63 | 0.05 | | 0.62 | 0.09 | |
| 533 | Benzyl Alcohol | Alcohol | 0.23 | 0.01 | | 0.19 | 0.03 | |
| 534 | Benzyl angelate | Ester | 0.02 | 0.01 | | 0.02 | 0.00 | |
| 535 | Benzyl nitrile | Nitrogen compounds | 0.04 | 0.00 | | 0.04 | 0.00 | |
| 536 | Bicyclo(3.3.1)non-2-ene | Hydrocarbons | 1.43 | 0.04 | | 1.32 | 0.06 | |
| 537 | Bicyclo[2.2.1]hept-2-ene, 1,7,7-trimethyl- | Terpenoids | 0.15 | 0.01 | | 0.13 | 0.02 | |
| 538 | Bicyclo[2.2.1]heptan-2-ol, 1,7,7-trimethyl-, formate, endo- | Ester | 0.21 | 0.03 | | 0.17 | 0.01 | |
| 539 | Bicyclo[2.2.1]heptan-2-ol, 2,3,3-trimethyl- | Terpenoids | 0.45 | 0.03 | | 0.41 | 0.01 | |
| 540 | Bicyclo[2.2.1]heptane, 7,7-dimethyl-2-methylene- | Terpenoids | 0.20 | 0.01 | | 0.18 | 0.01 | |
| 541 | Bicyclo[2.2.1]heptane-2,5-dione, 1,7,7-trimethyl- | Terpenoids | 0.16 | 0.08 | | 0.16 | 0.08 | |
| 542 | Bicyclo[3.1.0]hex-3-en-2-one, 4-methyl-1-(1-methylethyl)- | Terpenoids | 0.97 | 0.10 | | 0.83 | 0.01 | |
| 543 | Bicyclo[3.1.0]hexan-3-ol, 4-methylene-1-(1-methylethyl)-, acetate | Terpenoids | 0.12 | 0.01 | | 0.12 | 0.01 | |
| 544 | Bicyclo[3.1.0]hexane, 4-methylene-1-(1-methylethyl)- | Terpenoids | 0.06 | 0.00 | | 0.06 | 0.00 | |
| 545 | Bicyclo[3.1.1]hept-2-en-6-ol, 2,7,7-trimethyl-, acetate, [1S-(1.alpha.,5.alpha.,6.beta.)]- | Ester | 0.02 | 0.00 | | 0.01 | 0.00 | |
| 546 | Bicyclo[3.1.1]hept-2-en-6-one, 2,7,7-trimethyl- | Terpenoids | 0.07 | 0.00 | | 0.06 | 0.01 | |
| 547 | Bicyclo[3.1.1]hept-2-ene-2-carboxaldehyde, 6,6-dimethyl- | Terpenoids | 0.13 | 0.00 | | 0.11 | 0.02 | |
| 548 | Bicyclo[3.1.1]hept-2-ene-2-carboxylic acid, 6,6-dimethyl- | Terpenoids | 0.31 | 0.03 | | 0.28 | 0.00 | |
| 549 | Bicyclo[3.1.1]hept-2-ene-2-methanol, 6,6-dimethyl- | Terpenoids | 0.19 | 0.02 | | 0.14 | 0.04 | |
| 550 | Bicyclo[3.1.1]hept-3-en-2-one, 4,6,6-trimethyl-, (1S)- | Terpenoids | 0.47 | 0.05 | | 0.42 | 0.02 | |
| 551 | Bicyclo[3.1.1]heptan-2-one, 6,6-dimethyl- | Ketone | 0.15 | 0.00 | | 0.13 | 0.01 | |
| 552 | Bicyclo[3.1.1]heptan-3-ol, 6,6-dimethyl-2-methylene- | Terpenoids | 0.25 | 0.03 | | 0.23 | 0.02 | |
| 553 | Bicyclo[3.1.1]heptan-3-ol, 6,6-dimethyl-2-methylene-, [1S-(1.alpha.,3.alpha.,5.alpha.)]- | Terpenoids | 0.25 | 0.03 | | 0.23 | 0.02 | |
| 554 | Bicyclo[3.1.1]heptane, 6,6-dimethyl-2-methylene-, (1S)- | Terpenoids | 0.00 | 0.00 | | 0.00 | 0.00 | |
| 555 | Bicyclo[3.2.1]oct-2-ene, 3-methyl-4-methylene- | Terpenoids | 0.34 | 0.00 | | 0.22 | 0.15 | |
| 556 | Bicyclo[4.4.0]dec-1-ene, 2-isopropyl-5-methyl-9-methylene- | Terpenoids | 0.05 | 0.01 | | 0.03 | 0.00 | |
| 557 | Bicyclo[7.2.0]undecane, 10,10-dimethyl-2,6-bis(methylene)-, [1S-(1R*,9S*)]- | Terpenoids | 0.01 | 0.00 | | 0.01 | 0.00 | |
| 558 | Biphenyl | Aromatics | 0.01 | 0.00 | | 0.01 | 0.00 | |
| 559 | Bornyl acetate | Terpenoids | 0.58 | 0.02 | | 0.51 | 0.04 | |
| 560 | Butanedioic acid, dipropyl ester | Ester | 0.00 | 0.00 | | 0.00 | 0.00 | |
| 561 | Butanoic Acid, 2-methyl- | Acid | 1.54 | 0.15 | | 1.54 | 0.13 | |
| 562 | Butanoic Acid, 3-hydroxy-3-methyl- | Acid | 0.00 | 0.00 | | 0.00 | 0.00 | |
| 563 | Butanoic Acid, 3-methylbutyl ester | Ester | 0.01 | 0.01 | | 0.01 | 0.01 | |
| 564 | Butanoic Acid, 4-hydroxy- | Acid | 0.02 | 0.04 | | 0.00 | 0.00 | |
| 565 | Butanoic acid, 1-methyloctyl ester | Ester | 0.02 | 0.00 | | 0.02 | 0.00 | |
| 566 | Butanoic acid, 1-phenylethyl ester | Ester | 0.07 | 0.01 | | 0.07 | 0.01 | |
| 567 | Butanoic acid, 2-methyl-, 2-methylpropyl ester | Ester | 0.07 | 0.01 | | 0.06 | 0.00 | |
| 568 | Butanoic acid, 2-methyl-, hexyl ester | Ester | 0.01 | 0.01 | | 0.01 | 0.00 | |
| 569 | Butanoic acid, 2-methyl-, phenylmethyl ester | Ester | 0.00 | 0.00 | | 0.00 | 0.00 | |
| 570 | Butanoic acid, 2-methyl-, propyl ester | Ester | 0.01 | 0.00 | | 0.01 | 0.00 | |
| 571 | Butanoic acid, 3-hexenyl ester, (E)- | Ester | 0.07 | 0.00 | | 0.06 | 0.00 | |
| 572 | Butanoic acid, 3-hexenyl ester, (Z)- | Ester | 0.48 | 0.02 | | 0.41 | 0.03 | |
| 573 | Butanoic acid, 3-methyl-, 2-methylbutyl ester | Ester | 0.27 | 0.02 | | 0.25 | 0.04 | |
| 574 | Butanoic acid, 3-methyl-, hexyl ester | Ester | 0.18 | 0.05 | | 0.15 | 0.00 | |
| 575 | Butanoic acid, 3-methyl-3-nitroso-, methyl ester | Ester | 0.01 | 0.00 | | 0.01 | 0.00 | |
| 576 | Butanoic acid, anhydride | Others | 3.04 | 0.28 | | 2.49 | 0.40 | |
| 577 | Butanoic acid, butyl ester | Ester | 0.12 | 0.00 | | 0.11 | 0.00 | |
| 578 | Butanoic acid, heptyl ester | Ester | 0.04 | 0.00 | | 0.03 | 0.00 | |
| 579 | Butanoic acid, hexyl ester | Ester | 0.01 | 0.00 | | 0.01 | 0.00 | |
| 580 | Butanoic acid, octyl ester | Ester | 0.04 | 0.00 | | 0.04 | 0.01 | |
| 581 | Camphene | Terpenoids | 0.29 | 0.02 | | 0.26 | 0.02 | |
| 582 | Camphenone, 6- | Ketone | 0.92 | 0.12 | | 0.84 | 0.09 | |
| 583 | Camphor | Terpenoids | 1.22 | 0.11 | | 1.09 | 0.07 | |
| 584 | Caprolactam | Amine | 0.15 | 0.03 | | 0.17 | 0.01 | |
| 585 | Carbamic acid, 2-chloroethyl ester | Ester | 0.01 | 0.01 | | 0.01 | 0.00 | |
| 586 | Carbonochloridodithioic acid, methyl ester | Others | 0.02 | 0.00 | | 0.02 | 0.00 | |
| 587 | Carvenone | Terpenoids | 0.50 | 0.03 | | 0.45 | 0.01 | |
| 588 | Carveol | Terpenoids | 0.74 | 0.01 | | 0.83 | 0.07 | |
| 589 | Carvone | Terpenoids | 0.03 | 0.00 | | 0.03 | 0.00 | |
| 590 | Carvone oxide, trans- | Heterocyclic compound | 0.10 | 0.02 | | 0.09 | 0.00 | |
| 591 | Citral | Terpenoids | 0.04 | 0.01 | | 0.03 | 0.00 | |
| 592 | Citronellal | Terpenoids | 0.03 | 0.01 | | 0.03 | 0.00 | |
| 593 | Citronellyl isobutyrate | Ester | 0.60 | 0.02 | | 0.50 | 0.01 | |
| 594 | Coumarin | Heterocyclic compound | 0.02 | 0.00 | | 0.02 | 0.00 | |
| 595 | Cubenene | Terpenoids | 0.01 | 0.00 | | 0.01 | 0.00 | |
| 596 | Cyclobutanecarboxylic acid chloride | Halogenated hydrocarbons | 0.13 | 0.09 | | 0.16 | 0.11 | |
| 597 | Cycloheptanone, 3-methyl- | Ketone | 1.13 | 0.09 | | 1.01 | 0.20 | |
| 598 | Cyclohexane, (1,1-dimethylpropyl)- | Hydrocarbons | 0.19 | 0.03 | | 0.14 | 0.06 | |
| 599 | Cyclohexane, 1-methylene-4-(1-methylethenyl)- | Terpenoids | 0.01 | 0.00 | | 0.01 | 0.00 | |
| 600 | CyclohexanecarboxAldehyde | Aldehyde | 0.03 | 0.00 | | 0.03 | 0.00 | |
| 601 | Cyclohexanecarboxylic Acid | Acid | 0.02 | 0.01 | | 0.01 | 0.01 | |
| 602 | Cyclohexanecarboxylic acid, ethyl ester | Ester | 0.01 | 0.00 | | 0.01 | 0.00 | |
| 603 | Cyclohexanemethanol, .alpha.,.alpha.-dimethyl-4-methylene- | Alcohol | 0.27 | 0.07 | | 0.32 | 0.12 | |
| 604 | Cyclohexanepropanoic Acid, 2-propenyl ester | Ester | 0.02 | 0.02 | | 0.03 | 0.02 | |
| 605 | Cyclohexanol, 1-methyl-4-(1-methylethyl)-, cis- | Terpenoids | 0.54 | 0.05 | | 0.50 | 0.07 | |
| 606 | Cyclohexanol, 1-methyl-4-(1-methylethylidene)- | Terpenoids | 0.14 | 0.03 | | 0.16 | 0.04 | |
| 607 | Cyclohexanol, 2-methyl-3-(1-methylethenyl)-, (1.alpha.,2.alpha.,3.alpha.)- | Alcohol | 0.23 | 0.02 | | 0.18 | 0.03 | |
| 608 | Cyclohexanol, 3,5-dimethyl- | Alcohol | 0.00 | 0.00 | | 0.00 | 0.00 | |
| 609 | Cyclohexanone, 5-methyl-2-(1-methylethenyl)- | Terpenoids | 1.03 | 0.04 | | 0.97 | 0.03 | |
| 610 | Cyclohexanone, 5-methyl-2-(1-methylethyl)- | Terpenoids | 0.27 | 0.02 | | 0.23 | 0.02 | |
| 611 | Cyclohexanone, 5-methyl-2-(1-methylethylidene)- | Terpenoids | 0.17 | 0.11 | | 0.22 | 0.01 | |
| 612 | Cyclohexene, 1-methoxy- | Aromatics | 0.30 | 0.07 | | 0.32 | 0.02 | |
| 613 | Cyclohexene, 1-methyl-4-(1-methylethylidene)- | Terpenoids | 0.17 | 0.10 | | 0.10 | 0.01 | |
| 614 | Cyclohexene, 2,4-dimethyl-1-(1-methylethenyl)- | Hydrocarbons | 0.17 | 0.01 | | 0.15 | 0.02 | |
| 615 | Cyclohexene, 2-ethenyl-1,3,3-trimethyl- | Hydrocarbons | 0.44 | 0.03 | | 0.39 | 0.03 | |
| 616 | Cyclohexene, 3,4-diethenyl-1,6-dimethyl- | Hydrocarbons | 0.16 | 0.04 | | 0.15 | 0.01 | |
| 617 | Cyclohexene, 4-ethenyl- | Hydrocarbons | 2.50 | 0.12 | | 1.92 | 0.13 | |
| 618 | Cyclohexene, 6-(2-butenylidene)-1,5,5-trimethyl-, (E,Z)- | Hydrocarbons | 0.13 | 0.01 | | 0.12 | 0.01 | |
| 619 | Cyclooctanemethanol | Alcohol | 0.06 | 0.01 | | 0.05 | 0.01 | |
| 620 | Cyclopentanecarbonitrile, 3-(1-methylethylidene)- | Nitrogen compounds | 0.36 | 0.00 | | 0.31 | 0.02 | |
| 621 | Cyclopentanone, 2-(2-hexenyl)- | Ketone | 0.03 | 0.00 | | 0.03 | 0.00 | |
| 622 | Cyclopentanone, 2-(3-methyl-2-buten-1-yl)- | Ketone | 0.07 | 0.01 | | 0.06 | 0.01 | |
| 623 | D-Limonene | Terpenoids | 0.39 | 0.06 | | 0.37 | 0.06 | |
| 624 | D-Verbenone | Terpenoids | 0.34 | 0.04 | | 0.33 | 0.03 | |
| 625 | DL-Norvaline, ethyl ester | Ester | 0.02 | 0.00 | | 0.01 | 0.00 | |
| 626 | Decanal | Aldehyde | 0.03 | 0.00 | | 0.03 | 0.00 | |
| 627 | Decane, 2,4-dimethyl- | Hydrocarbons | 0.14 | 0.01 | | 0.12 | 0.01 | |
| 628 | Decane, 5-ethyl-5-methyl- | Hydrocarbons | 0.17 | 0.06 | | 0.12 | 0.01 | |
| 629 | Decanoic acid, methyl ester | Ester | 0.02 | 0.01 | | 0.02 | 0.00 | |
| 630 | Di-epi-.alpha.-cedrene | Terpenoids | 0.09 | 0.01 | | 0.07 | 0.00 | |
| 631 | Di-epi-.alpha.-cedrene-(I) | Terpenoids | 0.02 | 0.01 | | 0.01 | 0.00 | |
| 632 | DiSulfur compounds, dipropyl | Sulfur compounds | 0.02 | 0.00 | | 0.01 | 0.00 | |
| 633 | Diallyl disulphide | Sulfur compounds | 0.04 | 0.03 | | 0.02 | 0.02 | |
| 634 | Dicyclopentadiene | Hydrocarbons | 1.59 | 0.07 | | 1.43 | 0.06 | |
| 635 | Diethyl Phthalate | Ester | 0.01 | 0.01 | | 0.01 | 0.00 | |
| 636 | Diethyl adipate | Ester | 0.08 | 0.01 | | 0.07 | 0.01 | |
| 637 | Diethyl diSulfur compounds | Sulfur compounds | 0.02 | 0.00 | | 0.02 | 0.00 | |
| 638 | Dihydro-3-(2H)-thiophenone | Heterocyclic compound | 0.05 | 0.01 | | 0.05 | 0.00 | |
| 639 | Dihydrocarvyl acetate | Terpenoids | 0.28 | 0.01 | | 0.25 | 0.01 | |
| 640 | Dimethyl Sulfoxide | Sulfur compounds | 0.05 | 0.00 | | 0.06 | 0.01 | |
| 641 | Dimethyl phthalate | Ester | 0.93 | 0.16 | | 0.83 | 0.07 | |
| 642 | Dimethyl sulfone | Sulfur compounds | 0.45 | 0.11 | | 0.39 | 0.08 | |
| 643 | Dimethyl triSulfur compounds | Sulfur compounds | 0.25 | 0.02 | | 0.25 | 0.03 | |
| 644 | Dodecanal | Aldehyde | 0.02 | 0.00 | | 0.02 | 0.00 | |
| 645 | Dodecane | Hydrocarbons | 0.01 | 0.00 | | 0.01 | 0.00 | |
| 646 | E-2-Hexenyl benzoate | Ester | 0.02 | 0.00 | | 0.02 | 0.00 | |
| 647 | Ethanediamide | Amine | 0.01 | 0.00 | | 0.01 | 0.00 | |
| 648 | Ethanethioic acid, S-(2-furanylmethyl) ester | Heterocyclic compound | 0.07 | 0.01 | | 0.06 | 0.00 | |
| 649 | Ethanol, 2,2'-oxybis- | Alcohol | 0.04 | 0.01 | | 0.05 | 0.00 | |
| 650 | Ethanol, 2-(2-hydroxyethoxy)-, 1-nitrate | Alcohol | 0.08 | 0.01 | | 0.07 | 0.00 | |
| 651 | Ethanol, 2-(3,3-dimethylcyclohexylidene)-, (Z)- | Alcohol | 0.17 | 0.02 | | 0.15 | 0.02 | |
| 652 | Ethanol, 2-(4-chlorophenyl)amino- | Alcohol | 0.04 | 0.01 | | 0.04 | 0.01 | |
| 653 | Ethanol, 2-(4-ethylphenoxy)- | Alcohol | 0.37 | 0.02 | | 0.32 | 0.02 | |
| 654 | Ethanone, 1-(1,3-dimethyl-3-cyclohexen-1-yl)- | Ketone | 0.15 | 0.02 | | 0.11 | 0.01 | |
| 655 | Ethanone, 1-(1,4-dimethyl-3-cyclohexen-1-yl)- | Terpenoids | 0.37 | 0.09 | | 0.36 | 0.06 | |
| 656 | Ethanone, 1-(1H-pyrazol-4-yl)- | Heterocyclic compound | 0.06 | 0.01 | | 0.05 | 0.01 | |
| 657 | Ethanone, 1-(1H-pyrrol-2-yl)- | Heterocyclic compound | 14.09 | 0.58 | | 12.77 | 0.86 | |
| 658 | Ethanone, 1-(2,3,4-trihydroxyphenyl)- | Ketone | 0.22 | 0.02 | | 0.19 | 0.01 | |
| 659 | Ethanone, 1-(2,4-dihydroxyphenyl)- | Ketone | 0.03 | 0.01 | | 0.02 | 0.00 | |
| 660 | Ethanone, 1-(2,4-dimethyl-1H-pyrrol-3-yl)- | Heterocyclic compound | 1.02 | 0.23 | | 0.91 | 0.06 | |
| 661 | Ethanone, 1-(2-aminophenyl)- | Ketone | 26.51 | 1.87 | | 25.81 | 0.52 | |
| 662 | Ethanone, 1-(2-furanyl)- | Heterocyclic compound | 34.76 | 2.31 | | 31.46 | 0.37 | |
| 663 | Ethanone, 1-(2-hydroxy-5-methylphenyl)- | Ketone | 0.13 | 0.03 | | 0.15 | 0.02 | |
| 664 | Ethanone, 1-(2-methyl-1-cyclopenten-1-yl)- | Ketone | 7.72 | 0.31 | | 6.51 | 0.55 | |
| 665 | Ethanone, 1-(2-methylphenyl)- | Ketone | 0.17 | 0.01 | | 0.15 | 0.01 | |
| 666 | Ethanone, 1-(2-pyridinyl)- | Heterocyclic compound | 0.58 | 0.02 | | 0.47 | 0.02 | |
| 667 | Ethanone, 1-(3,5-dimethylpyrazinyl)- | Heterocyclic compound | 0.11 | 0.01 | | 0.09 | 0.01 | |
| 668 | Ethanone, 1-(3-methylphenyl)- | Ketone | 1.07 | 0.33 | | 0.80 | 0.27 | |
| 669 | Ethanone, 1-(4-ethylphenyl)- | Ketone | 0.09 | 0.01 | | 0.08 | 0.00 | |
| 670 | Ethanone, 1-(4-hydroxy-3-thienyl)- | Heterocyclic compound | 0.07 | 0.00 | | 0.06 | 0.01 | |
| 671 | Ethanone, 1-(5-methyl-1-phenyl-1H-pyrazol-4-yl)- | Heterocyclic compound | 0.01 | 0.00 | | 0.01 | 0.00 | |
| 672 | Ethinamate | Ester | 0.27 | 0.13 | | 0.25 | 0.10 | |
| 673 | Ethyl (methylthio)acetate | Ester | 0.02 | 0.00 | | 0.02 | 0.00 | |
| 674 | Ethyl 2-hexenoate, trans- | Ester | 0.23 | 0.09 | | 0.08 | 0.10 | |
| 675 | Ethyl hydrogen succinate | Ester | 1.28 | 0.68 | | 0.80 | 0.14 | |
| 676 | Ethyl mandelate | Ester | 0.10 | 0.01 | | 0.09 | 0.00 | |
| 677 | Eugenol | Phenol | 0.92 | 0.02 | | 0.80 | 0.06 | |
| 678 | Fenchol | Terpenoids | 1.93 | 0.57 | | 1.38 | 0.06 | |
| 679 | Formic acid, octyl ester | Ester | 0.30 | 0.05 | | 0.28 | 0.03 | |
| 680 | Formic acid, phenylmethyl ester | Ester | 0.76 | 0.10 | | 0.72 | 0.02 | |
| 681 | Fumaric Acid | Acid | 0.12 | 0.01 | | 0.11 | 0.04 | |
| 682 | Furan, 2-ethyl-5-methyl- | Heterocyclic compound | 0.08 | 0.01 | | 0.06 | 0.01 | |
| 683 | Furan, 2-hexyl- | Heterocyclic compound | 0.07 | 0.00 | | 0.07 | 0.01 | |
| 684 | Furan, 2-propyl- | Heterocyclic compound | 0.01 | 0.01 | | 0.01 | 0.01 | |
| 685 | Furanodienone | Terpenoids | 0.01 | 0.00 | | 0.01 | 0.00 | |
| 686 | Geranic acid | Terpenoids | 0.02 | 0.00 | | 0.02 | 0.00 | |
| 687 | Geranyl acetate | Terpenoids | 0.53 | 0.02 | | 0.49 | 0.03 | |
| 688 | Geranyl formate | Ester | 0.34 | 0.02 | | 0.29 | 0.01 | |
| 689 | Germacrene D | Terpenoids | 0.02 | 0.00 | | 0.02 | 0.00 | |
| 690 | Glycerin | Alcohol | 0.10 | 0.04 | | 0.08 | 0.01 | |
| 691 | Glycylsarcosine | Acid | 0.07 | 0.01 | | 0.05 | 0.00 | |
| 692 | Heptane, 1,1-dimethoxy- | Hydrocarbons | 0.01 | 0.00 | | 0.01 | 0.00 | |
| 693 | Heptane, 2,2,4,6,6-pentamethyl- | Hydrocarbons | 0.32 | 0.10 | | 0.18 | 0.01 | |
| 694 | Hexadecane | Hydrocarbons | 0.02 | 0.00 | | 0.02 | 0.00 | |
| 695 | Hexanal | Aldehyde | 0.14 | 0.01 | | 0.17 | 0.02 | |
| 696 | Hexanoic acid, 1-methylhexyl ester | Ester | 0.01 | 0.00 | | 0.01 | 0.00 | |
| 697 | Hexanoic acid, 2-methylbutyl ester | Ester | 0.03 | 0.01 | | 0.03 | 0.01 | |
| 698 | Hexanoic acid, 2-methylpropyl ester | Ester | 0.84 | 0.06 | | 0.57 | 0.37 | |
| 699 | Hexanoic acid, 3-hexenyl ester | Ester | 0.02 | 0.00 | | 0.02 | 0.00 | |
| 700 | Hexanoic acid, 3-hexenyl ester, (Z)- | Ester | 0.03 | 0.01 | | 0.02 | 0.00 | |
| 701 | Hexanoic acid, 3-hydroxy-, ethyl ester | Ester | 0.04 | 0.01 | | 0.03 | 0.02 | |
| 702 | Hexanoic acid, 3-oxo-, ethyl ester | Ester | 3.04 | 0.28 | | 2.49 | 0.40 | |
| 703 | Hexanoic acid, cyclopentyl ester | Ester | 0.01 | 0.01 | | 0.01 | 0.01 | |
| 704 | Hexanoic acid, ethyl ester | Ester | 0.69 | 0.48 | | 0.41 | 0.47 | |
| 705 | Hexanoic acid, hexyl ester | Ester | 0.02 | 0.02 | | 0.00 | 0.00 | |
| 706 | Hinesol | Terpenoids | 0.01 | 0.00 | | 0.00 | 0.00 | |
| 707 | Hydrocinnamic Acid | Acid | 0.04 | 0.01 | | 0.04 | 0.00 | |
| 708 | Hydrocoumarin | Heterocyclic compound | 0.05 | 0.00 | | 0.04 | 0.00 | |
| 709 | Ibuprofen | Acid | 0.01 | 0.00 | | 0.01 | 0.00 | |
| 710 | Indan, 1-methyl- | Aromatics | 0.01 | 0.00 | | 0.01 | 0.00 | |
| 711 | Indane | Aromatics | 0.03 | 0.00 | | 0.02 | 0.00 | |
| 712 | Indole | Heterocyclic compound | 2.74 | 0.30 | | 2.56 | 0.16 | |
| 713 | Indole, 3-methyl- | Heterocyclic compound | 0.28 | 0.03 | | 0.22 | 0.03 | |
| 714 | Indole-3-acetAldehyde | Heterocyclic compound | 0.00 | 0.00 | | 0.00 | 0.00 | |
| 715 | Ionone | Terpenoids | 0.08 | 0.01 | | 0.06 | 0.01 | |
| 716 | Iridomyrmecin | Terpenoids | 0.13 | 0.01 | | 0.11 | 0.01 | |
| 717 | Iso-3-thujyl acetate | Ester | 0.18 | 0.02 | | 0.17 | 0.01 | |
| 718 | Isoborneol | Terpenoids | 0.45 | 0.02 | | 0.41 | 0.02 | |
| 719 | Isobornyl acetate | Ester | 0.58 | 0.02 | | 0.51 | 0.04 | |
| 720 | Isobutyl isovalerate | Ester | 0.07 | 0.01 | | 0.06 | 0.00 | |
| 721 | Isophorone | Ketone | 0.39 | 0.02 | | 0.35 | 0.04 | |
| 722 | Isopropenyl ethyl ketone | Ketone | 0.22 | 0.01 | | 0.20 | 0.02 | |
| 723 | Isoquinoline | Heterocyclic compound | 0.03 | 0.00 | | 0.03 | 0.00 | |
| 724 | Isoquinoline, 1-butyl-3,4-dihydro- | Heterocyclic compound | 0.01 | 0.00 | | 0.01 | 0.00 | |
| 725 | Kessane | Terpenoids | 0.03 | 0.01 | | 0.02 | 0.00 | |
| 726 | Ketone, methyl 2,2,3-trimethylcyclopentyl | Ketone | 0.05 | 0.00 | | 0.05 | 0.00 | |
| 727 | L-.alpha.-Terpineol | Terpenoids | 1.24 | 0.06 | | 1.09 | 0.09 | |
| 728 | L-Norleucine, methyl ester | Ester | 0.02 | 0.01 | | 0.02 | 0.00 | |
| 729 | Lilac Aldehyde C | Aldehyde | 0.08 | 0.01 | | 0.19 | 0.01 | |
| 730 | Lilac Aldehyde D | Aldehyde | 0.08 | 0.01 | | 0.19 | 0.01 | |
| 731 | Linalool | Terpenoids | 0.08 | 0.00 | | 0.07 | 0.01 | |
| 732 | Linalyl acetate | Terpenoids | 0.78 | 0.50 | | 0.98 | 0.02 | |
| 733 | Longifolenaldehyde | Terpenoids | 0.02 | 0.01 | | 0.02 | 0.00 | |
| 734 | Maltol | Heterocyclic compound | 12.94 | 2.10 | | 9.26 | 0.60 | |
| 735 | Megastigmatrienone | Ketone | 0.07 | 0.00 | | 0.06 | 0.00 | |
| 736 | Memantine | Amine | 0.28 | 0.00 | | 0.25 | 0.03 | |
| 737 | Methyl 5-hydroxynicotinate | Ester | 0.96 | 0.13 | | 0.74 | 0.05 | |
| 738 | Methyl 6,6-dimethylbicyclo[3.1.1]hept-2-ene-2-carboxylate | Ester | 0.03 | 0.00 | | 0.02 | 0.00 | |
| 739 | Methyl 6-chloronicotinate | Ester | 0.04 | 0.01 | | 0.04 | 0.00 | |
| 740 | Methyl ethyl diSulfur compounds | Sulfur compounds | 0.03 | 0.00 | | 0.02 | 0.00 | |
| 741 | Methyl methacrylate | Ester | 0.63 | 0.05 | | 0.60 | 0.04 | |
| 742 | Methyl p-tolyloxyacetate | Ester | 0.04 | 0.01 | | 0.03 | 0.00 | |
| 743 | Methyl salicylate | Ester | 0.23 | 0.07 | | 0.21 | 0.03 | |
| 744 | Methylparaben | Ester | 0.01 | 0.00 | | 0.01 | 0.00 | |
| 745 | Myroxide | Terpenoids | 0.29 | 0.07 | | 0.20 | 0.05 | |
| 746 | N-Benzyloxy-2-carbomethoxyaziridine | Heterocyclic compound | 0.02 | 0.00 | | 0.02 | 0.00 | |
| 747 | Naphthalene | Aromatics | 0.34 | 0.04 | | 0.27 | 0.03 | |
| 748 | Naphthalene, 1,2,3,4,4a,7-hexahydro-1,6-dimethyl-4-(1-methylethyl)- | Terpenoids | 0.01 | 0.00 | | 0.01 | 0.00 | |
| 749 | Naphthalene, 1,2,3,4-tetrahydro- | Aromatics | 0.02 | 0.01 | | 0.01 | 0.00 | |
| 750 | Naphthalene, 1,2,3,4-tetrahydro-1,6-dimethyl-4-(1-methylethyl)-, (1S-cis)- | Terpenoids | 0.15 | 0.02 | | 0.13 | 0.01 | |
| 751 | Naphthalene, 1,2,3,4-tetrahydro-2,6-dimethyl- | Aromatics | 0.26 | 0.02 | | 0.21 | 0.04 | |
| 752 | Naphthalene, 1,2,3,5,6,8a-hexahydro-4,7-dimethyl-1-(1-methylethyl)-, (1S-cis)- | Terpenoids | 0.01 | 0.00 | | 0.01 | 0.00 | |
| 753 | Naphthalene, 1,2,4a,5,6,8a-hexahydro-4,7-dimethyl-1-(1-methylethyl)-, [1S-(1.alpha.,4a.beta.,8a.alpha.)]- | Terpenoids | 0.01 | 0.00 | | 0.01 | 0.00 | |
| 754 | Naphthalene, 1,2,4a,5,6,8a-hexahydro-4,7-dimethyl-1-(1-methylethyl)-,[1R-(1.alpha.,4a.alpha.,8a.alpha.)]- | Terpenoids | 0.03 | 0.00 | | 0.03 | 0.00 | |
| 755 | Naphthalene, 1,2,4a,5,8,8a-hexahydro-4,7-dimethyl-1-(1-methylethyl)-, (1.alpha.,4a.beta.,8a.alpha.)-(.+/-.)- | Terpenoids | 0.03 | 0.02 | | 0.03 | 0.00 | |
| 756 | Naphthalene, 1,2,4a,5,8,8a-hexahydro-4,7-dimethyl-1-(1-methylethyl)-, [1S-(1.alpha.,4a.beta.,8a.alpha.)]- | Terpenoids | 0.01 | 0.00 | | 0.01 | 0.00 | |
| 757 | Naphthalene, 1,2-dihydro-1,1,6-trimethyl- | Aromatics | 0.07 | 0.01 | | 0.05 | 0.00 | |
| 758 | Naphthalene, 2,3,6-trimethyl- | Aromatics | 0.10 | 0.02 | | 0.10 | 0.01 | |
| 759 | Naphthalene, 2,6-dimethyl- | Aromatics | 0.01 | 0.00 | | 0.01 | 0.00 | |
| 760 | Naphthalene, decahydro-4a-methyl-1-methylene-7-(1-methylethenyl)-, [4aR-(4a.alpha.,7.alpha.,8a.beta.)]- | Terpenoids | 0.01 | 0.00 | | 0.01 | 0.00 | |
| 761 | Nonane | Hydrocarbons | 0.02 | 0.00 | | 0.02 | 0.00 | |
| 762 | Nonane, 3,7-dimethyl- | Hydrocarbons | 0.08 | 0.07 | | 0.08 | 0.05 | |
| 763 | Nonane, 4,5-dimethyl- | Hydrocarbons | 0.00 | 0.00 | | 0.00 | 0.00 | |
| 764 | Nonane, 5-(2-methylpropyl)- | Hydrocarbons | 0.32 | 0.02 | | 0.19 | 0.12 | |
| 765 | Nonanoic Acid | Acid | 0.05 | 0.01 | | 0.04 | 0.00 | |
| 766 | Nonanoic acid, 9-oxo-, methyl ester | Ester | 0.02 | 0.00 | | 0.01 | 0.00 | |
| 767 | Nonanoic acid, methyl ester | Ester | 0.01 | 0.00 | | 0.01 | 0.00 | |
| 768 | Norbornadieone | Ketone | 1.11 | 0.27 | | 0.83 | 0.39 | |
| 769 | Octanal | Aldehyde | 0.30 | 0.01 | | 0.31 | 0.02 | |
| 770 | Octane | Hydrocarbons | 0.36 | 0.02 | | 0.36 | 0.01 | |
| 771 | Octane, 2,3,3-trimethyl- | Hydrocarbons | 0.03 | 0.01 | | 0.03 | 0.01 | |
| 772 | Octane, 2,6,6-trimethyl- | Hydrocarbons | 0.02 | 0.01 | | 0.02 | 0.00 | |
| 773 | Octane, 5-ethyl-2-methyl- | Hydrocarbons | 0.03 | 0.00 | | 0.03 | 0.00 | |
| 774 | Octanoic acid, ethyl ester | Ester | 0.01 | 0.00 | | 0.00 | 0.00 | |
| 775 | Octanoic acid-tert butyl ester | Ester | 0.20 | 0.02 | | 0.18 | 0.01 | |
| 776 | Orcinol | Phenol | 0.11 | 0.00 | | 0.10 | 0.01 | |
| 777 | OxiranecarboxAldehyde, 3-methyl-3-(4-methyl-3-pentenyl)- | Aldehyde | 0.79 | 0.24 | | 0.59 | 0.04 | |
| 778 | Panaxene | Terpenoids | 0.23 | 0.04 | | 0.23 | 0.01 | |
| 779 | Pantolactone | Ester | 0.05 | 0.04 | | 0.03 | 0.04 | |
| 780 | Paroxypropione | Ketone | 0.06 | 0.00 | | 0.05 | 0.00 | |
| 781 | Pentadecane | Hydrocarbons | 0.05 | 0.01 | | 0.04 | 0.00 | |
| 782 | Pentadecane, 2,6,10,14-tetramethyl- | Hydrocarbons | 0.02 | 0.01 | | 0.01 | 0.00 | |
| 783 | Pentanal O-benzyloxime | Nitrogen compounds | 0.04 | 0.01 | | 0.04 | 0.00 | |
| 784 | Pentanoic Acid, 2-methyl- | Acid | 0.05 | 0.01 | | 0.04 | 0.01 | |
| 785 | Pentanoic acid, 2-hydroxy-3-methyl-, methyl ester | Ester | 0.06 | 0.00 | | 0.06 | 0.00 | |
| 786 | Pentanoic acid, 2-hydroxy-4-methyl-, methyl ester | Ester | 0.03 | 0.02 | | 0.01 | 0.02 | |
| 787 | Pentanoic acid, 2-methyl-, ethyl ester | Ester | 0.29 | 0.01 | | 0.23 | 0.06 | |
| 788 | Pentanoic acid, 4-methyl-, ethyl ester | Ester | 0.35 | 0.06 | | 0.34 | 0.01 | |
| 789 | Phenol | Phenol | 0.59 | 0.03 | | 0.50 | 0.06 | |
| 790 | Phenol, 2,3,6-trimethyl- | Phenol | 0.80 | 0.07 | | 0.69 | 0.11 | |
| 791 | Phenol, 2,4-dichloro- | Phenol | 1.15 | 0.04 | | 1.01 | 0.05 | |
| 792 | Phenol, 2,4-dinitro- | Phenol | 0.00 | 0.00 | | 0.00 | 0.00 | |
| 793 | Phenol, 2-ethyl- | Phenol | 0.10 | 0.01 | | 0.09 | 0.01 | |
| 794 | Phenol, 2-methoxy-4-propyl- | Aromatics | 0.67 | 0.19 | | 0.44 | 0.07 | |
| 795 | Phenol, 2-methyl- | Phenol | 0.39 | 0.03 | | 0.32 | 0.03 | |
| 796 | Phenol, 2-methyl-5-(1-methylethyl)- | Terpenoids | 0.97 | 0.04 | | 0.86 | 0.09 | |
| 797 | Phenol, 2-nitro- | Phenol | 0.14 | 0.00 | | 0.13 | 0.01 | |
| 798 | Phenol, 3,5-dimethyl- | Phenol | 0.14 | 0.03 | | 0.15 | 0.01 | |
| 799 | Phenol, 3-ethyl- | Phenol | 0.14 | 0.03 | | 0.15 | 0.01 | |
| 800 | Phenol, 4-butyl- | Phenol | 0.04 | 0.00 | | 0.05 | 0.03 | |
| 801 | Phenol, 4-ethyl-2-methoxy- | Phenol | 12.70 | 1.94 | | 8.54 | 0.35 | |
| 802 | Phenol, 4-hexyl- | Phenol | 0.01 | 0.00 | | 0.01 | 0.00 | |
| 803 | Phenol, 4-methyl-2-nitro- | Phenol | 0.32 | 0.14 | | 0.24 | 0.13 | |
| 804 | Phenol, m-tert-butyl- | Phenol | 0.14 | 0.01 | | 0.10 | 0.03 | |
| 805 | Phenol, p-tert-butyl- | Phenol | 0.14 | 0.01 | | 0.10 | 0.03 | |
| 806 | Phenylacetic acid propyl ester | Ester | 0.31 | 0.02 | | 0.28 | 0.02 | |
| 807 | Picolinamide | Heterocyclic compound | 0.03 | 0.02 | | 0.04 | 0.00 | |
| 808 | Pinocarvone | Terpenoids | 2.27 | 0.12 | | 2.22 | 0.10 | |
| 809 | Piperidin-3-one, 2,2,5-methyl- | Heterocyclic compound | 0.04 | 0.01 | | 0.03 | 0.00 | |
| 810 | Piperitenone oxide | Ketone | 0.03 | 0.00 | | 0.02 | 0.00 | |
| 811 | Propanedioic acid, amino-, diethyl ester | Ester | 0.01 | 0.00 | | 0.01 | 0.00 | |
| 812 | Propanethial, S-oxide | Sulfur compounds | 0.01 | 0.00 | | 0.01 | 0.00 | |
| 813 | Propanoic Acid, 2-oxo- | Acid | 0.10 | 0.00 | | 0.09 | 0.00 | |
| 814 | Propanoic acid, 2-methyl-, 2-methylbutyl ester | Ester | 0.18 | 0.12 | | 0.23 | 0.04 | |
| 815 | Propanoic acid, 2-methyl-, 2-phenylethyl ester | Ester | 0.05 | 0.00 | | 0.04 | 0.00 | |
| 816 | Propanoic acid, 2-methyl-, 3-phenylpropyl ester | Ester | 0.13 | 0.01 | | 0.12 | 0.00 | |
| 817 | Propanoic acid, 3-(methylthio)- | Sulfur compounds | 0.00 | 0.00 | | 0.00 | 0.00 | |
| 818 | Propanoic acid, butyl ester | Ester | 0.07 | 0.01 | | 0.04 | 0.02 | |
| 819 | Pyrazine, 2,3-dimethyl- | Heterocyclic compound | 3.92 | 0.23 | | 3.76 | 0.73 | |
| 820 | Pyrazine, 2,3-dimethyl-5-(1-methylpropyl)- | Heterocyclic compound | 1.36 | 0.04 | | 1.45 | 0.17 | |
| 821 | Pyrazine, 2,5-dimethyl- | Heterocyclic compound | 136.34 | 7.98 | | 121.54 | 5.73 | |
| 822 | Pyrazine, 2,6-dimethyl- | Heterocyclic compound | 136.34 | 7.98 | | 121.54 | 5.73 | |
| 823 | Pyrazine, 2-ethyl-3,5-dimethyl- | Heterocyclic compound | 13.12 | 1.63 | | 13.27 | 1.90 | |
| 824 | Pyrazine, 2-ethyl-3-methyl- | Heterocyclic compound | 73.14 | 3.20 | | 67.17 | 3.43 | |
| 825 | Pyrazine, 2-ethyl-5-methyl- | Heterocyclic compound | 73.14 | 3.20 | | 67.17 | 3.43 | |
| 826 | Pyrazine, 2-methoxy-3-(1-methylethyl)- | Heterocyclic compound | 1.52 | 0.06 | | 1.69 | 0.22 | |
| 827 | Pyrazine, 2-methoxy-3-(1-methylpropyl)- | Heterocyclic compound | 1.13 | 0.10 | | 1.01 | 0.12 | |
| 828 | Pyrazine, 2-methoxy-3-methyl- | Heterocyclic compound | 0.21 | 0.04 | | 0.17 | 0.06 | |
| 829 | Pyrazine, 2-methoxy-6-methyl- | Heterocyclic compound | 0.21 | 0.04 | | 0.17 | 0.06 | |
| 830 | Pyrazine, 2-methyl-3-(methylthio)- | Heterocyclic compound | 5.15 | 0.34 | | 4.67 | 0.29 | |
| 831 | Pyrazine, 2-methyl-5-(1-methylethyl)- | Heterocyclic compound | 0.32 | 0.05 | | 0.28 | 0.02 | |
| 832 | Pyrazine, 2-methyl-6-(methylthio)- | Heterocyclic compound | 5.15 | 0.34 | | 4.67 | 0.29 | |
| 833 | Pyrazine, 3,5-diethyl-2-methyl- | Heterocyclic compound | 1.97 | 0.22 | | 1.83 | 0.10 | |
| 834 | Pyrazine, 3-ethyl-2,5-dimethyl- | Heterocyclic compound | 13.12 | 1.63 | | 13.27 | 1.90 | |
| 835 | Pyrazine, methoxy- | Heterocyclic compound | 0.52 | 0.02 | | 0.53 | 0.08 | |
| 836 | Pyrazine, methyl- | Heterocyclic compound | 72.28 | 3.00 | | 65.41 | 2.94 | |
| 837 | Pyrazine, tetramethyl- | Heterocyclic compound | 0.34 | 0.06 | | 0.34 | 0.02 | |
| 838 | Pyrazine, trimethyl- | Heterocyclic compound | 4.10 | 0.37 | | 3.36 | 0.43 | |
| 839 | Pyridine, 2-ethyl- | Heterocyclic compound | 0.83 | 0.03 | | 0.79 | 0.01 | |
| 840 | Pyridine, 2-methyl- | Heterocyclic compound | 2.49 | 0.14 | | 2.32 | 0.10 | |
| 841 | Pyridine, 2-pentyl- | Heterocyclic compound | 0.54 | 0.16 | | 0.51 | 0.09 | |
| 842 | Pyridine, 3-(3,4-dihydro-2H-pyrrol-5-yl)- | Heterocyclic compound | 0.03 | 0.00 | | 0.03 | 0.00 | |
| 843 | Quinoline | Heterocyclic compound | 0.21 | 0.16 | | 0.21 | 0.06 | |
| 844 | Quinoline, 2,7-dimethyl- | Heterocyclic compound | 0.11 | 0.01 | | 0.09 | 0.00 | |
| 845 | Quinoxaline, 2-methyl- | Heterocyclic compound | 0.45 | 0.08 | | 0.44 | 0.03 | |
| 846 | Salvial-4(14)-en-1-one | Terpenoids | 0.04 | 0.00 | | 0.02 | 0.02 | |
| 847 | Santolina triene | Hydrocarbons | 0.15 | 0.01 | | 0.13 | 0.02 | |
| 848 | Styrene | Aromatics | 0.11 | 0.02 | | 0.09 | 0.02 | |
| 849 | Sulfurous acid, dimethyl ester | Sulfur compounds | 0.12 | 0.00 | | 0.11 | 0.01 | |
| 850 | TRANS-ANETHOLE | Aromatics | 0.21 | 0.01 | | 0.19 | 0.01 | |
| 851 | Terpinen-4-ol | Terpenoids | 0.04 | 0.03 | | 0.02 | 0.00 | |
| 852 | Tetradecane | Hydrocarbons | 0.46 | 0.10 | | 0.38 | 0.06 | |
| 853 | Tetradecane, 4-methyl- | Hydrocarbons | 0.17 | 0.04 | | 0.14 | 0.03 | |
| 854 | Thiazole, 2,4,5-trimethyl- | Heterocyclic compound | 0.08 | 0.02 | | 0.07 | 0.01 | |
| 855 | Thiazole, 2,4-dimethyl- | Heterocyclic compound | 0.10 | 0.01 | | 0.08 | 0.02 | |
| 856 | Thiazole, 4,5-dimethyl- | Heterocyclic compound | 0.09 | 0.06 | | 0.05 | 0.05 | |
| 857 | Thieno[2,3-b]thiophene,2-methyl- | Heterocyclic compound | 0.02 | 0.00 | | 0.02 | 0.00 | |
| 858 | Thiirane, (methoxymethyl)- | Heterocyclic compound | 0.00 | 0.00 | | 0.00 | 0.00 | |
| 859 | Thiophene, 2-butyl-5-ethyl- | Heterocyclic compound | 0.02 | 0.00 | | 0.01 | 0.01 | |
| 860 | Thiophene, 2-methyl- | Heterocyclic compound | 0.04 | 0.01 | | 0.04 | 0.01 | |
| 861 | Thiophene, 2-pentyl- | Heterocyclic compound | 0.32 | 0.06 | | 0.27 | 0.07 | |
| 862 | Thiophene, 3-methyl- | Heterocyclic compound | 0.04 | 0.01 | | 0.04 | 0.01 | |
| 863 | Thujone | Terpenoids | 0.02 | 0.00 | | 0.02 | 0.01 | |
| 864 | Thymol | Terpenoids | 0.97 | 0.04 | | 0.86 | 0.09 | |
| 865 | Tolbutamide | Others | 0.02 | 0.00 | | 0.02 | 0.00 | |
| 866 | TriSulfur compounds, dipropyl | Sulfur compounds | 0.00 | 0.00 | | 0.00 | 0.00 | |
| 867 | TriSulfur compounds, methyl 2-propenyl | Sulfur compounds | 0.35 | 0.03 | | 0.31 | 0.02 | |
| 868 | Tricyclo[2.2.1.0(2,6)]heptane-3-methanol, 2,3-dimethyl- | Terpenoids | 0.30 | 0.01 | | 0.25 | 0.03 | |
| 869 | Tricyclo[3.3.1.1(3,7)]decane, 2-nitro- | Nitrogen compounds | 0.21 | 0.01 | | 0.18 | 0.03 | |
| 870 | Tridecane, 2-methyl- | Hydrocarbons | 0.04 | 0.01 | | 0.03 | 0.01 | |
| 871 | Undecane, 2,4-dimethyl- | Hydrocarbons | 0.17 | 0.06 | | 0.12 | 0.01 | |
| 872 | Undecane, 2,5-dimethyl- | Hydrocarbons | 0.17 | 0.06 | | 0.12 | 0.01 | |
| 873 | Undecane, 2,6-dimethyl- | Hydrocarbons | 0.17 | 0.06 | | 0.12 | 0.01 | |
| 874 | Undecane, 2,9-dimethyl- | Hydrocarbons | 0.17 | 0.06 | | 0.12 | 0.01 | |
| 875 | Undecane, 3,5-dimethyl- | Hydrocarbons | 0.17 | 0.06 | | 0.12 | 0.01 | |
| 876 | Undecane, 3,6-dimethyl- | Hydrocarbons | 0.17 | 0.06 | | 0.12 | 0.01 | |
| 877 | Undecane, 4,4-dimethyl- | Hydrocarbons | 0.05 | 0.03 | | 0.04 | 0.02 | |
| 878 | Undecane, 4-methyl- | Hydrocarbons | 0.21 | 0.15 | | 0.12 | 0.15 | |
| 879 | Undecane, 6-ethyl- | Hydrocarbons | 0.17 | 0.06 | | 0.12 | 0.01 | |
| 880 | Undecanoic acid, methyl ester | Ester | 0.01 | 0.00 | | 0.01 | 0.00 | |
| 881 | Undecanol-5 | Alcohol | 0.15 | 0.03 | | 0.14 | 0.01 | |
| 882 | Vanillin | Aldehyde | 0.65 | 0.10 | | 0.48 | 0.01 | |
| 883 | Verbenone | Terpenoids | 0.47 | 0.05 | | 0.42 | 0.02 | |
| 884 | [1aR-(1a.alpha.,4.alpha.,4a.beta.,7b.alpha.)]-1a,2,3,4,4a,5,6,7b-octahydro-1,1,4,7-tetramethyl-1H-Cycloprop[e]azulene | Terpenoids | 0.01 | 0.00 | | 0.01 | 0.00 | |
| 885 | aR-Himachalene | Terpenoids | 0.04 | 0.02 | | 0.04 | 0.01 | |
| 886 | cis-.alpha.-Bergamotene | Terpenoids | 0.05 | 0.01 | | 0.05 | 0.01 | |
| 887 | cis-3-Hexenyl cis-3-hexenoate | Ester | 0.02 | 0.00 | | 0.02 | 0.00 | |
| 888 | cis-Dihydrocarvone | Terpenoids | 0.73 | 0.05 | | 0.70 | 0.02 | |
| 889 | dl-Camphoroquinone | Terpenoids | 0.13 | 0.02 | | 0.13 | 0.01 | |
| 890 | exo-7-(trans-1-Propenyl)bicyclo[4.2.0]oct-1(2)-ene | Hydrocarbons | 0.06 | 0.00 | | 0.05 | 0.00 | |
| 891 | n-Caproic acid vinyl ester | Ester | 0.04 | 0.00 | | 0.03 | 0.00 | |
| 892 | n-Decanoic Acid | Acid | 0.01 | 0.00 | | 0.00 | 0.00 | |
| 893 | n-Heptyl hexanoate | Ester | 0.03 | 0.00 | | 0.02 | 0.00 | |
| 894 | n-Hexylacetoacetic acid ethyl ester | Ester | 0.02 | 0.01 | | 0.02 | 0.00 | |
| 895 | n-Pentyl methylphosphonofluoridate | Others | 0.02 | 0.02 | | 0.02 | 0.02 | |
| 896 | o-Hydroxybiphenyl | Phenol | 0.01 | 0.00 | | 0.01 | 0.00 | |
| 897 | o-Mentha-1(7),8-dien-3-ol | Terpenoids | 0.00 | 0.00 | | 0.00 | 0.00 | |
| 898 | o-Xylene | Aromatics | 0.01 | 0.00 | | 0.01 | 0.00 | |
| 899 | p-Cresol | Phenol | 3.20 | 0.04 | | 3.00 | 0.41 | |
| 900 | p-Cymen-7-ol | Alcohol | 1.74 | 0.12 | | 1.52 | 0.16 | |
| 901 | p-Isopropoxyaniline | Amine | 0.08 | 0.02 | | 0.09 | 0.01 | |
| 902 | p-Menth-2-en-7-ol, cis- | Phenol | 0.14 | 0.03 | | 0.16 | 0.04 | |
| 903 | p-Menth-8-en-3-ol, acetate | Ester | 0.51 | 0.06 | | 0.50 | 0.08 | |
| 904 | p-Mentha-1,8-dien-7-ol | Terpenoids | 0.02 | 0.00 | | 0.02 | 0.00 | |
| 905 | p-Menthane-3,8-diol, cis-1,3,trans-1,4- | Terpenoids | 2.08 | 0.04 | | 1.83 | 0.21 | |
| 906 | p-Tolylacetic Acid | Acid | 0.18 | 0.02 | | 0.15 | 0.01 | |
| 907 | p-Xylene | Aromatics | 0.11 | 0.02 | | 0.10 | 0.03 | |
| 908 | trans,cis-2,6-Nonadien-1-ol | Alcohol | 0.05 | 0.00 | | 0.04 | 0.00 | |
| 909 | trans-.alpha.-Bergamotene | Terpenoids | 0.06 | 0.01 | | 0.04 | 0.00 | |
| 910 | trans-.beta.-Ocimene | Terpenoids | 0.15 | 0.08 | | 0.09 | 0.07 | |
| 911 | trans-3-Methyl-4-octanolide | Ester | 0.08 | 0.01 | | 0.06 | 0.01 | |
| 912 | trans-4-MethoxycinnamAldehyde | Aldehyde | 0.21 | 0.04 | | 0.17 | 0.06 | |
| 913 | trans-Geranic acid methyl ester | Terpenoids | 0.09 | 0.02 | | 0.10 | 0.00 | |
| 914 | trans-Isoeugenol | Phenol | 0.29 | 0.05 | | 0.28 | 0.02 | |
| 915 | trans-O-Dithiane-4,5-diol | Heterocyclic compound | 0.01 | 0.00 | | 0.00 | 0.00 | |
| 916 | β-Isocomene | Terpenoids | 0.07 | 0.01 | | 0.06 | 0.00 | |

**Table S2.** The odor of volatile compounds in coffee samples.

| No. | Compounds | Class I | Odor |
| --- | --- | --- | --- |
| 1 | (+)-3-Carene | Terpenoids | sweet |
| 2 | (+)-Diethyl L-tartrate | Ester | mild, fruity, wine, caramel |
| 3 | (-)-.beta.-Bourbonene | Terpenoids | herbal, woody, floral, balsamic |
| 4 | (-)-Myrtenol | Terpenoids | woody, pine, balsamic, sweet, minty |
| 5 | (1.alpha.,4a.beta.,8a.alpha.)-1,2,3,4,4a,5,6,8a-octahydro-7-methyl-4-methylene-1-(1-methylethyl)-Naphthalene | Terpenoids | herbal, woody |
| 6 | (1R)-(-)-Myrtenal | Aldehyde | sweet, cinnamon, tonka, spicy, terpene, camphor, jammy |
| 7 | (2E,4Z)-2,4-Decadienal | Aldehyde | fried, fatty, geranium, green, waxy |
| 8 | (3R,6R)-2,2,6-Trimethyl-6-vinyltetrahydro-2H-pyran-3-ol | Heterocyclic compound | citrus, green |
| 9 | (4aR-trans)-decahydro-4a-methyl-1-methylene-7-(1-methylethylidene)-Naphthalene | Terpenoids | woody |
| 10 | (6,6-dimethyl-2-bicyclo[3.1.1]hept-2-enyl)methyl 2-methylpropanoate | Ester | fruity, woody, pine |
| 11 | (6Z)-Nonen-1-ol | Alcohol | fresh, green, melon, waxy, honeydew, cantaloupe, cucumber, clean |
| 12 | (E)-2-Decenal | Aldehyde | waxy, fatty, earthy, green, cilantro, mushroom, aldehydic, fried, chicken, fatty, tallow |
| 13 | (E)-2-Heptenal | Aldehyde | pungent, green, vegetable, fresh, fatty |
| 14 | (E)-sec-Butyl propenyl diSulfur compounds | Sulfur compounds | garlic |
| 15 | (E,E)-2,4-Undecadienal | Aldehyde | oily, caramel, spicy, citrus, buttery, baked |
| 16 | .alpha.-Cubebene | Terpenoids | herbal, waxy |
| 17 | .alpha.-Ionone | Terpenoids | sweet, woody, floral, violet, orris, tropical, fruity |
| 18 | .alpha.-Irone | Terpenoids | orris, floral, berry, violet, woody, powdery |
| 19 | .alpha.-Muurolene | Terpenoids | woody |
| 20 | .alpha.-Phellandrene 1 | Terpenoids | citrus, herbal, terpene, green, woody, peppery |
| 21 | .alpha.-Terpineol | Terpenoids | pine, iris, teil |
| 22 | .beta.-Ocimene | Terpenoids | apple, pear, fruity |
| 23 | .beta.-Phellandrene | Terpenoids | terpenic, herbal |
| 24 | .beta.-Phenylethyl butyrate | Ester | musty, sweet, floral, yeasty, strawberry |
| 25 | .beta.-Phenylethylmethylethylcarbinol | Alcohol | sweet, fresh, cayloxol, lilac, anisic, earthy, narcissus, peony |
| 26 | .beta.-Pinene | Terpenoids | dry, woody, resinous, pine, hay, green |
| 27 | .beta.-Santalol | Terpenoids | woody |
| 28 | .delta.-Dodecalactone | Ester | peachy, oily, creamy, soapy |
| 29 | .delta.-Nonalactone | Ester | coconut, creamy, sweet, milky, coumarin |
| 30 | .gamma.-Elemene | Terpenoids | green, woody, oily |
| 31 | 1,1'-Biphenyl, 3-methyl- | Aromatics | green, rose |
| 32 | 1,2-Cyclohexanedione | Ketone | sweet, acorn, nut skin, maple, caramel, brothy |
| 33 | 1,2-Propanedione, 1-phenyl- | Ketone | plastic, buttery, honey |
| 34 | 1,3,6-Octatriene, 3,7-dimethyl-, (Z)- | Terpenoids | warm, floral, herbal, flowery, sweet |
| 35 | 1,3,7-Octatriene, 3,7-dimethyl- | Terpenoids | fruity, floral, wet, cloth |
| 36 | 1,3-Cyclohexadiene, 5-(1,5-dimethyl-4-hexenyl)-2-methyl-, [S-(R*,S*)]- | Terpenoids | spice, fresh, sharp |
| 37 | 1,3-Cyclohexadiene-1-carboxaldehyde, 2,6,6-trimethyl- | Terpenoids | fresh, herbal, phenol, metallic, rosemary, tobacco, spicy |
| 38 | 1,3-Cyclohexadiene-1-carboxylic acid, 2,6,6-trimethyl-, ethyl ester | Ester | fresh, natural, herbal, damascone, rose, apple, woody |
| 39 | 1,3-Dioxolane, 4-methyl-2-(2-methylpropyl)- | Heterocyclic compound | sweet, ethereal, fruity, tropical, rummy, sour, dairy, buttery |
| 40 | 1,3-Hexadiene, 3-ethyl-2-methyl- | Hydrocarbons | nutty |
| 41 | 1,4-Dithiane | Amine | sulfury, solvent, garlic, onion, pyridine |
| 42 | 1,5-Dimethyl-1-vinyl-4-hexenyl butyrate | Ester | bergamot, fruity, banana, berry |
| 43 | 1,5-Heptadien-4-one, 3,3,6-trimethyl- | Terpenoids | herbal, honey, minty, berry |
| 44 | 1-(4-methylphenyl)-Ethanone | Ketone | green, pea, bell pepper, galbanum |
| 45 | 1-Butanol, 3-methyl-, formate | Ester | plum, black currant, ethereal, vinegar, dry earthy, fruity, green |
| 46 | 1-Cyclohexene-1-carboxAldehyde, 4-(1-methylethenyl)- | Aldehyde | fresh, green, herbal, grassy, sweet, minty, cumin |
| 47 | 1-Cyclohexene-1-carboxAldehyde, 4-(1-methylethenyl)-, (S)- | Aldehyde | fresh, green, oily, grassy, fatty, minty, cherry |
| 48 | 1-Dodecanol | Alcohol | earthy, soapy, waxy, fatty, honey, coconut |
| 49 | 1-Octen-3-one | Ketone | mushroom |
| 50 | 1-Oxaspiro[4.5]dec-6-ene, 2,6,10,10-tetramethyl- | Terpenoids | tea, herbal, green, wet, tobacco, leafy, metallic, woody, spicy |
| 51 | 1-Propanone, 1-(2-furanyl)- | Heterocyclic compound | caramel, sweet |
| 52 | 1-Propanone, 1-(4-methoxyphenyl)- | Ketone | musty, anisic |
| 53 | 1-Undecanol | Alcohol | fresh, waxy, rose, soapy, clean, cloth, floral, citrus |
| 54 | 1-methyl-4-(1-methylethenyl)-1,2-Cyclohexanediol | Terpenoids | cool, minty |
| 55 | 1H-3a,7-Methanoazulene, 2,3,4,7,8,8a-hexahydro-3,6,8,8-tetramethyl-, [3R-(3.alpha.,3a.beta.,7.beta.,8a.alpha.)]- | Terpenoids | woody, cedary, sweet, fresh |
| 56 | 1H-Cyclopenta[1,3]cyclopropa[1,2]benzene, octahydro-7-methyl-3-methylene-4-(1-methylethyl)-, [3aS-(3a.alpha.,3b.beta.,4.beta.,7.alpha.,7aS*)]- | Terpenoids | citrus, fruity, radish |
| 57 | 1H-Cycloprop[e]azulen-7-ol, decahydro-1,1,7-trimethyl-4-methylene-, [1ar-(1a.alpha.,4a.alpha.,7.beta.,7a.beta.,7b.alpha.)]- | Terpenoids | earthy, herbal, fruity |
| 58 | 1H-Pyrrole-2-carboxAldehyde | Heterocyclic compound | musty, beefy, coffee |
| 59 | 2(3H)-Furanone, 5-butyldihydro- | Ester | sweet, coconut, waxy, creamy, tonka, dairy, fatty |
| 60 | 2(3H)-Furanone, dihydro-5-propyl- | Ester | sweet, coconut, nutty, caramel, tonka, hay |
| 61 | 2,3-Dehydro-1,8-cineole | Terpenoids | minty, lemon |
| 62 | 2,3-Dimethyl-5-ethylpyrazine | Heterocyclic compound | burnt, popcorn, roasted, cocoa |
| 63 | 2,3-Pentanedione | Ketone | pungent, sweet, buttery, creamy, caramel, nutty, cheese |
| 64 | 2,4,6-Octatriene, 2,6-dimethyl- | Terpenoids | sweet, floral, nut skin, peppery, herbal, tropical |
| 65 | 2,4,6-Octatriene, 2,6-dimethyl-, (E,E)- | Terpenoids | terpene, sweet, fresh, floral |
| 66 | 2,4-Cycloheptadien-1-one, 2,6,6-trimethyl- | Terpenoids | minty |
| 67 | 2,4-Decadien-1-ol | Alcohol | fatty, waxy, citrus, melon |
| 68 | 2,4-Decadienoic acid, ethyl ester, (E,Z)- | Ester | green, waxy, pear, apple, sweet, fruity, tropical |
| 69 | 2,4-Di-tert-butylphenol | Phenol | phenol |
| 70 | 2,4-Heptadien-1-ol, (E,E)- | Alcohol | green, fruity, nutty, cheese |
| 71 | 2,4-Hexadienoic acid, ethyl ester, (2E,4E)- | Ester | warm, fruity, anisic, licorice, ether |
| 72 | 2,4-Octadienal, (E,E)- | Aldehyde | green, fatty, pear, melon, peel |
| 73 | 2,6,6-Trimethyl-2-cyclohexene-1,4-dione | Ketone | musty, woody, sweet, tea, tobacco, leafy |
| 74 | 2,6-Dodecadien-1-al | Aldehyde | citrus, mandarin, orange, melon |
| 75 | 2,6-Nonadien-1-ol | Alcohol | green, cucumber, vegetable |
| 76 | 2,6-Nonadienal, (E,E)- | Aldehyde | fresh, citrus, green, cucumber, melon |
| 77 | 2,6-Nonadienal, (E,Z)- | Aldehyde | cucumber, green |
| 78 | 2,6-Octadien-1-ol, 3,7-dimethyl-, acetate, (Z)- | Ester | floral, rose, soapy, citrus, dewy, pear |
| 79 | 2,6-Octadienal, 3,7-dimethyl-, (E)- | Terpenoids | citrus, lemon |
| 80 | 2,6-Octadienenitrile, 3,7-dimethyl-, (Z)- | Nitrogen compounds | citral, lemon, aldehydic, metallic |
| 81 | 2-((3,3-Dimethyloxiran-2-yl)methyl)-3-methylfuran | Heterocyclic compound | green, earthy, citrus |
| 82 | 2-Acetyl-1,4,5,6-tetrahydropyridine | Heterocyclic compound | creamy, bread |
| 83 | 2-Acetyl-1-pyrroline | Heterocyclic compound | popcorn, toasted, grain, malty |
| 84 | 2-Acetyl-3-ethylpyrazine | Heterocyclic compound | earthy, potato, chip, nutty, popcorn, corn |
| 85 | 2-Acetyl-3-methylpyrazine | Heterocyclic compound | nutty, flesh, roasted hazelnut, toasted grain, corn, chip, vegetable, nut skin, caramel |
| 86 | 2-Acetyl-5-methylfuran | Heterocyclic compound | strong, musty, nutty, hay, coconut, coumarin, milky |
| 87 | 2-Acetylthiazole | Heterocyclic compound | nutty, popcorn, roasted, peanut, hazelnut |
| 88 | 2-Butanone, 4-(4-hydroxyphenyl)- | Ketone | sweet, berry, jammy, raspberry, ripe, floral |
| 89 | 2-Buten-1-one, 1-(2,6,6-trimethyl-1,3-cyclohexadien-1-yl)-, (E)- | Terpenoids | apple, rose, honey, tobacco, sweet |
| 90 | 2-Buten-1-one, 1-(2,6,6-trimethyl-1-cyclohexen-1-yl)- | Ketone | fruity, floral, black currant, currant, plum, rose, honey, tobacco |
| 91 | 2-Butenal, 2-methyl-4-(2,6,6-trimethyl-1-cyclohexen-1-yl)- | Aldehyde | dry, sweet, tobacco, nutty, violet, boronia, dry leaf, hay, woody, acorn |
| 92 | 2-Butenoic acid, 3-hexenyl ester, (E,Z)- | Ester | green, vegetable |
| 93 | 2-Butenoic acid, ethyl ester, (E)- | Ester | pungent, chemical, diffusive, sweet, alliaceous, caramel, rummy |
| 94 | 2-Butenoic acid, hexyl ester | Ester | sweet, caramel, green, walnut, oily, radish |
| 95 | 2-Cyclohexen-1-ol, 1-methyl-4-(1-methylethyl)-, cis- | Alcohol | herbal |
| 96 | 2-Cyclohexen-1-ol, 2-methyl-5-(1-methylethenyl)-, acetate | Ester | green, minty, spearmint, nasturtium, herbal, rummy, grape, pear, spicy |
| 97 | 2-Cyclohexen-1-one | Ketone | roasted, savory, green |
| 98 | 2-Cyclohexen-1-one, 2-hydroxy-3-methyl-6-(1-methylethyl)- | Terpenoids | minty, buchu, leafy, black currant, bud, phenol, corn mint, menthol, herbal, woody |
| 99 | 2-Cyclohexen-1-one, 3-methyl- | Ketone | nutty, caramel, phenol, cherry |
| 100 | 2-Cyclohexen-1-one, 3-methyl-6-(1-methylethyl)- | Terpenoids | herbal, minty, camphor, medicinal |
| 101 | 2-Cyclopenten-1-one, 2-pentyl- | Ketone | woody, isojasmone, jasmin, tuberose |
| 102 | 2-Cyclopenten-1-one, 3-methyl-2-(2-pentenyl)-, (Z)- | Ketone | woody, herbal, floral, spicy, jasmin, celery |
| 103 | 2-Ethoxy-3-methylpyrazine | Heterocyclic compound | hazelnut, roasted, almond, pineapple, earthy |
| 104 | 2-Ethyl-3-methoxypyrazine | Heterocyclic compound | raw, potato, earthy, bell pepper, nutty |
| 105 | 2-FurancarboxAldehyde, 5-methyl- | Aldehyde | spice, caramel, maple |
| 106 | 2-Furancarboxylic acid, octyl ester | Ester | oily, waxy, earthy, mushroom, creamy, tallow |
| 107 | 2-Furanmethanethiol, 5-methyl- | Heterocyclic compound | sulfury, roasted, coffee |
| 108 | 2-Heptanol | Alcohol | fruity, mouldy, musty, mushroom |
| 109 | 2-Hexanol | Alcohol | chemical, winey, fruity, fatty, terpenic, cauliflower |
| 110 | 2-Isobutylthiazole | Heterocyclic compound | green, wasabi, privet, tomato, leafy, earthy, vegetable, metallic |
| 111 | 2-Methoxy-4-vinylphenol | Aromatics | spicy, raisin |
| 112 | 2-Methyl-1,3-dithiacyclopentane | Heterocyclic compound | sulfury, alliaceous, smoky, savory, vegetable |
| 113 | 2-Methyl-3(2-furyl)acrolein | Heterocyclic compound | spice, cinnamon, woody, herbal |
| 114 | 2-Methyl-3-furanthiol | Heterocyclic compound | sulfury, meaty, fishy, metallic |
| 115 | 2-Nonanol | Alcohol | rose |
| 116 | 2-Nonanone | Ketone | fruity, floral, fatty |
| 117 | 2-Nonen-1-ol | Alcohol | sweet, fatty, melon, cucumber, vegetable |
| 118 | 2-Nonen-1-ol, (E)- | Alcohol | waxy, green, violet, melon |
| 119 | 2-Octanol | Alcohol | fresh, spicy, green, woody, herbal, earthy |
| 120 | 2-Octanone | Ketone | earthy, weedy, natural, woody, herbal |
| 121 | 2-Octen-1-ol, (E)- | Alcohol | green, citrus, vegetable, fatty |
| 122 | 2-Octenal, 2-butyl- | Alcohol | aldehydic, green, watery, fruity, metallic, oily, tropical, fatty, sweaty, goaty |
| 123 | 2-Phenoxyethyl isobutyrate | Ester | green, fruity, waxy, apple, nuances |
| 124 | 2-Propen-1-ol, 3-phenyl- | Alcohol | sweet, balsamic, hyacinth, spicy, green, powdery, cinnamon |
| 125 | 2-Propen-1-ol, 3-phenyl-, (E)- | Alcohol | sweet, balsamic, hyacinth, spicy, green, powdery, cinnamyl |
| 126 | 2-Propenal, 2-methyl-3-phenyl- | Aldehyde | sweet, cinnamon, spicy, cassia |
| 127 | 2-Propenoic Acid, 3-phenyl- | Acid | balsamic, sweet, storax |
| 128 | 2-Propenoic acid, 3-phenyl-, methyl ester, (E)- | Ester | sweet, balsamic, strawberry, cherry |
| 129 | 2-Thiophenemethanethiol | Heterocyclic compound | roasted, coffee, fishy |
| 130 | 2-Tridecanone | Ketone | fatty, waxy, dairy, milky, coconut, nutty, herbal, earthy |
| 131 | 2-Undecanone | Ketone | waxy, fruity, creamy, fatty, orris, floral |
| 132 | 2-Undecanone, 6,10-dimethyl- | Ketone | dry, musty |
| 133 | 2-hydroxy-BenzAldehyde | Aldehyde | medicnal, spicy, cinnamon, wintergreen, cool |
| 134 | 2-methoxy-Phenol | Phenol | nutty |
| 135 | 2-methyl-5-(1-methylethenyl)-Cyclohexanol | Terpenoids | minty, menthol, spearmint, herbal |
| 136 | 2H-Pyran, 3,6-dihydro-4-methyl-2-(2-methyl-1-propenyl)- | Terpenoids | green, weedy, cortex, herbal, diphenyl, narcissus, celery |
| 137 | 2H-Pyran-2-one, 6-hexyltetrahydro- | Ester | creamy, fatty, coconut, fruity, peach, waxy |
| 138 | 2H-Pyran-2-one, 6-pentyl- | Heterocyclic compound | tonka, lactonic, coconut, creamy, fatty, waxy |
| 139 | 2H-Pyran-2-one, tetrahydro- | Heterocyclic compound | estery, fruity, sweet |
| 140 | 2H-Pyran-2-one, tetrahydro-6-pentyl- | Ester | creamy, coconut, fruity |
| 141 | 2H-Pyran-2-one, tetrahydro-6-propyl- | Heterocyclic compound | coconut |
| 142 | 3(2H)-Furanone, 4-methoxy-2,5-dimethyl- | Heterocyclic compound | sweet, moldy, mushroom, vegetable, potato, burnt, sugar, nut skin, wasabi, caramel, fruity, brandy |
| 143 | 3(2H)-Thiophenone, dihydro-2-methyl- | Heterocyclic compound | sulfury, fruity, berry |
| 144 | 3,4-Hexanedione | Ketone | buttery, toasted, almond, nutty, caramel |
| 145 | 3,5-Octadien-2-one | Ketone | fruity, fatty, mushroom |
| 146 | 3,5-Octadien-2-one, (E,E)- | Ketone | fruity, green, grassy |
| 147 | 3,6-Dimethyl-2,3,3a,4,5,7a-hexahydrobenzofuran | Heterocyclic compound | herbal, dill |
| 148 | 3,6-Nonadien-1-ol, (E,Z)- | Alcohol | fatty, green, cucumber, green pepper, fruity, watermelon |
| 149 | 3-Acetyl-2,5-dimethyl furan | Heterocyclic compound | sweet, musty, nutty, earthy, cocoa, corn, leathery |
| 150 | 3-Buten-2-ol, 4-(2,6,6-trimethyl-2-cyclohexen-1-yl)-, (3E)- | Alcohol | ionone, tropical, sweet, floral, violet, woody |
| 151 | 3-Buten-2-one, 4-(2,2,6-trimethyl-7-oxabicyclo[4.1.0]hept-1-yl)- | Terpenoids | fruity, sweet, berry, woody, violet, orris, powdery |
| 152 | 3-Carene | Terpenoids | citrus, terpenic, herbal, pine, solvent, resinous, phenol, cypress, medicinal, woody |
| 153 | 3-Cyclohexen-1-ol, 1-methyl-4-(1-methylethyl)- | Terpenoids | dry, woody, musty |
| 154 | 3-Cyclohexene-1-acetAldehyde, .alpha.,4-dimethyl- | Aldehyde | spicy, herbal |
| 155 | 3-Cyclohexene-1-ethanol, .beta.,4-dimethyl- | Alcohol | fruity, herbal |
| 156 | 3-Cyclohexene-1-methanethiol, .alpha.,.alpha.,4-trimethyl- | Sulfur compounds | sulfury, aromatic, grapefruit, naphthyl, resinous, woody |
| 157 | 3-Cyclohexene-1-methanol, .alpha.,.alpha.,4-trimethyl-, propanoate | Terpenoids | herbal, green, old wood, citrus, geranium, tropical |
| 158 | 3-Hexen-1-ol, acetate, (E)- | Ester | sharp, fruity, green, cortex, hyacinth, narcissus, rummy, unripe banana, pear |
| 159 | 3-Hexen-1-ol, acetate, (Z)- | Ester | fresh, green, sweet, fruity, banana, apple, grassy |
| 160 | 3-Hexen-1-ol, formate, (Z)- | Ester | green, waxy, fresh, vegetable, fruity, apple, guava, green, banana |
| 161 | 3-Hexen-1-ol, propanoate, (Z)- | Ester | green, fresh, fruity, apple, pear, vegetable, melon, banana, peach |
| 162 | 3-Hexenal, (Z)- | Aldehyde | green, fatty, grassy, weedy, fruity, apple |
| 163 | 3-Hexene, 1-(1-ethoxyethoxy)-, (Z)- | Aldehyde | green, violet, natural, green, leafy, mushroom, earthy |
| 164 | 3-Mercaptohexanol | Alcohol | sulfury, fruity, tropical |
| 165 | 3-Mercaptohexyl acetate | Ester | sulfury, grapefruit, fruity |
| 166 | 3-Methoxy-2,5-dimethylpyrazine | Heterocyclic compound | earthy |
| 167 | 3-Methoxy-5-methylphenol | Phenol | oakmoss, fruity, iodine, woody, hay |
| 168 | 3-Nonen-1-ol, (E)- | Alcohol | green, waxy, melon, cucumber |
| 169 | 3-Octanol | Alcohol | earthy, mushroom, herbal, melon, citrus, woody, spicy, minty |
| 170 | 3-Octanone | Ketone | fresh, herbal, lavender, sweet, mushroom |
| 171 | 3-Octen-2-one | Ketone | earthy, spicy, herbal, sweet, mushroom, hay, blueberry |
| 172 | 3-Octen-2-one, (E)- | Ketone | herbal, mushroom |
| 173 | 3-Octen-2-one, 7-methyl- | Ketone | fatty, oily, lactonic, nutty, meaty, tallow, fatty |
| 174 | 3-Oxatricyclo[4.1.1.0(2,4)]octane, 2,7,7-trimethyl- | Terpenoids | green |
| 175 | 3-Penten-2-one, 4-methyl- | Ketone | pungent, earthy, vegetable, acrylate |
| 176 | 3-Phenyl-1-propanol, acetate | Ester | sweet, balsamic, storax, spicy, cinnamon |
| 177 | 3-Phenylpropanol | Alcohol | sweet, spicy, cinnamyl, mignonette, hyacinth, balsamic |
| 178 | 4-Decenal, (E)- | Aldehyde | fresh, aldehydic, citrus, orange, mandarin, tangerine, green, fatty |
| 179 | 4-Decenoic acid, methyl ester, Z- | Ester | fruity, pear, mango, fishy, peach skin, green |
| 180 | 4-Heptenal, (Z)- | Aldehyde | oily, fatty, green, dairy, milky, creamy |
| 181 | 4-Hexen-1-ol, 5-methyl-2-(1-methylethenyl)-, (R)- | Terpenoids | herbal |
| 182 | 4-Hydroxy-3-methoxybenzyl Alcohol | Alcohol | sweet, creamy, vanilla, caramel, coconut, graham, cracker, tonka, milky, powdery |
| 183 | 4-Isopropylcyclohexa-1,3-dienecarbAldehyde | Aldehyde | fatty, spicy |
| 184 | 4-MethoxycinnamAldehyde | Aldehyde | spicy, cinnamon, sweet, cherry, vanilla, floral |
| 185 | 4-Methylthiazole | Heterocyclic compound | nutty, green, vegetable, tomato |
| 186 | 4-Nonenal, (E)- | Aldehyde | fruity |
| 187 | 4-Phenyl-2-butanol | Alcohol | floral, peony, foliage, sweet, mimosa, heliotrope |
| 188 | 4-Undecanone | Ketone | fruity |
| 189 | 4-tert-Butylcyclohexyl acetate | Ester | woody, cedary, floral, oily, herbal, balsamic, green, fruity |
| 190 | 5,6,7,8-Tetrahydroquinoxaline | Heterocyclic compound | musty, nutty, roasted, beany, cereal, corn, chip, cheese |
| 191 | 5,9-Undecadien-2-one, 6,10-dimethyl- | Terpenoids | fresh, rose, leafy, floral, green, magnolia, aldehydic, fruity |
| 192 | 5,9-Undecadien-2-one, 6,10-dimethyl-, (E)- | Ketone | fresh, green, fruity, waxy, rose, woody, magnolia, tropical |
| 193 | 5-Acetyl-2,4-dimethylthiazole | Heterocyclic compound | meaty, sulfury, nutty, boiled, meaty |
| 194 | 5-Azulenemethanol, 1,2,3,4,5,6,7,8-octahydro-.alpha.,.alpha.,3,8-tetramethyl-, acetate, [3S-(3.alpha.,5.alpha.,8.alpha.)]- | Ester | tea, rose, woody, spicy, green, fatty |
| 195 | 5-Hepten-2-ol, 6-methyl- | Alcohol | sweet, oily, green, coriander |
| 196 | 5-Methyl-(E)-2-hepten-4-one | Ketone | hazelnut, nutty |
| 197 | 5-Methyl-2-thiophenecarboxaldehyde | Heterocyclic compound | sweet, almond, cherry, furfural, woody, acetophenone |
| 198 | 5H-5-Methyl-6,7-dihydrocyclopentapyrazine | Heterocyclic compound | earthy, baked, potato, peanut, roasted |
| 199 | 6-Nonenal, (Z)- | Aldehyde | green, cucumber, melon, cantaloupe, honeydew, waxy, vegetable, orris, violet, leafy |
| 200 | 6-Octen-1-ol, 3,7-dimethyl-, acetate | Ester | floral, green, rose, fruity, citrus, woody, tropical, fruity |
| 201 | 6-Octen-1-ol, 3,7-dimethyl-, formate | Terpenoids | bergamot, cucumber, rose, apricot, peach, plum |
| 202 | 6-Octenal, 7-methyl-3-methylene- | Aldehyde | juicy, aldehydic, lemon grass, floral, lemon, bois, rose |
| 203 | Acetic Acid, phenoxy- | Acid | sour, sweet |
| 204 | Acetic acid, 2-ethylhexyl ester | Ester | earthy, herbal, humus, undergrowth |
| 205 | Acetic acid, 2-phenylethyl ester | Ester | floral, rose, sweet, honey, fruity, tropical |
| 206 | Acetic acid, cyclohexyl ester | Ester | fruity, sweet, musty, ethereal |
| 207 | Acetic acid, nonyl ester | Ester | waxy, clean, green, tropical, fruity |
| 208 | Acetic acid, phenyl ester | Ester | phenol, medicinal, animalic, resinous, castoreum, woody, smoky, burnt |
| 209 | Acetoacetic acid isoamyl ester | Ester | ethereal, sweet, wine, green, fermented |
| 210 | Acetophenone | Ketone | sweet, pungent, hawthorn, mimosa, almond, acacia |
| 211 | Anethole | Aromatics | sweet, exotic, flowery, stewed |
| 212 | Apocynin | Ketone | faint, sweet, vanillin |
| 213 | BenzAldehyde | Aldehyde | sweet, bitter, almond, cherry |
| 214 | BenzAldehyde diethylacetal | Aldehyde | green, bitter, almond |
| 215 | BenzAldehyde, 3,4-dimethoxy- | Aldehyde | sweet, woody, vanilla |
| 216 | BenzAldehyde, 4-hydroxy- | Aldehyde | sweet, nutty, almond, balsamic, woody |
| 217 | BenzAldehyde, 4-methoxy- | Aldehyde | sweet, powdery, mimosa, floral, hawthorn, balsamic |
| 218 | BenzAldehyde, 4-methyl- | Aldehyde | fruity, cherry, deep, phenol |
| 219 | Benzene, (2,2-dimethoxyethyl)- | Aromatics | green, foliage, floral, rosy, earthy, mushroom |
| 220 | Benzene, (2-methoxyethyl)- | Aromatics | green, floral, chrysanthemum, jasmin, metallic, rose |
| 221 | Benzene, (2-nitroethyl)- | Aromatics | flowery, spice |
| 222 | Benzene, (butoxymethyl)- | Ether | floral, rose |
| 223 | Benzene, (isothiocyanatomethyl)- | Sulfur compounds | mild, watercress, dusty, medicinal, horseradish, oily |
| 224 | Benzene, (methoxymethyl)- | Ether | fruity, ethereal, ylang-ylang, green, floral, hyacinth |
| 225 | Benzene, (methylthio)- | Sulfur compounds | toluene, solvent, spicy, woody, sawdust |
| 226 | Benzene, 1,2,4,5-tetramethyl- | Aromatics | rancid, sweet |
| 227 | Benzene, 1,3-dimethyl- | Aromatics | plastic |
| 228 | Benzene, 1-(1,5-dimethyl-4-hexenyl)-4-methyl- | Terpenoids | herbal |
| 229 | Benzene, 1-ethyl-4-methoxy- | Aromatics | anisic |
| 230 | Benzene, 1-methoxy-4-(1-propenyl)-, (Z)- | Ether | anisic |
| 231 | BenzeneacetAldehyde | Aldehyde | floral, honey, rose, cherry |
| 232 | BenzeneacetAldehyde, .alpha.-(2-methylpropylidene)- | Aldehyde | sweet, cocoa, nutty, rose, powdery |
| 233 | BenzeneacetAldehyde, .alpha.-ethylidene- | Aldehyde | sweet, narcissus, cortex, beany, honey, cocoa, nutty, radish |
| 234 | Benzeneacetic acid | Acid | sweet, honey, floral, honeysuckle, sour, waxy, civet |
| 235 | Benzeneacetic acid, 2-methylpropyl ester | Ester | sweet, floral, honey, chocolate, amber |
| 236 | Benzeneacetic acid, ethyl ester | Ester | minty |
| 237 | Benzeneethanol, 4-hydroxy- | Alcohol | mild, sweet, floral, fruity |
| 238 | Benzenemethanol, .alpha.,.alpha.,4-trimethyl- | Terpenoids | sweet, fruity, cherry, coumarin, floral, camphor |
| 239 | Benzenemethanol, .alpha.,4-dimethyl- | Alcohol | sweet, hawthorn, floral, nutty, powdery |
| 240 | Benzenemethanol, .alpha.-methyl- | Alcohol | fresh, sweet, gardenia, hyacinth |
| 241 | Benzenemethanol, 4-methoxy- | Phenol | sweet, powdery, hawthorn, lilac, rose, floral, hyacinth |
| 242 | Benzenemethanol, 4-methyl- | Alcohol | mild, floral |
| 243 | Benzenepropanoic acid, ethyl ester | Ester | caramel, fruity |
| 244 | Benzofuran | Heterocyclic compound | aromatic |
| 245 | Benzofuran, 4,5,6,7-tetrahydro-3,6-dimethyl- | Terpenoids | pungent, musty, nutty, earthy, coffee |
| 246 | Benzoic acid | Acid | faint, balsamic, urine |
| 247 | Benzoic acid, 1-methylethyl ester | Ester | sweet, fruity, floral, balsamic |
| 248 | Benzoic acid, 2-(dimethylamino)-, methyl ester | Ester | fruity, orange, leafy, petitgrain |
| 249 | Benzoic acid, 2-(methylamino)-, methyl ester | Ester | fruity, musty, sweet, neroli, powdery, phenol, wine |
| 250 | Benzoic acid, 2-hydroxy-, ethyl ester | Ester | caramel, pepperminty |
| 251 | Benzoic acid, 2-methylpropyl ester | Ester | sweet, fruity, musty, powdery, balsamic |
| 252 | Benzoic acid, 2-propenyl ester | Ester | sweet, floral, cherry, berry |
| 253 | Benzoic acid, ethyl ester | Ester | fruity, dry, musty, sweet, wintergreen |
| 254 | Benzoic acid, hexyl ester | Ester | fresh, balsamic, sappy, woody |
| 255 | Benzophenone | Ketone | balsamic, rose, metallic, powdery, geranium |
| 256 | Benzothiazole | Heterocyclic compound | meaty, vegetable, brown, cooked, beefy, coffee |
| 257 | Benzoxazole, 2-methyl- | Heterocyclic compound | tobacco, burnt, phenol, meaty, powdery, capers |
| 258 | Benzyl Alcohol | Alcohol | floral, rose, phenol, balsamic |
| 259 | Bicyclo[2.2.1]heptan-2-ol, 1,7,7-trimethyl-, formate, endo- | Ester | green, earthy, herbal, balsamic, pine |
| 260 | Bicyclo[2.2.1]heptan-2-ol, 2,3,3-trimethyl- | Terpenoids | menthol, musty, camphor |
| 261 | Bicyclo[2.2.1]heptane, 7,7-dimethyl-2-methylene- | Terpenoids | camphor |
| 262 | Bicyclo[3.1.0]hex-3-en-2-one, 4-methyl-1-(1-methylethyl)- | Terpenoids | minty, pungent |
| 263 | Bicyclo[3.1.0]hexane, 4-methylene-1-(1-methylethyl)- | Terpenoids | woody, terpene, citrus, pine, spice |
| 264 | Bicyclo[3.1.1]hept-2-ene-2-carboxaldehyde, 6,6-dimethyl- | Terpenoids | sweet, cinnamon, tonka, spicy, terpene, camphor, jammy |
| 265 | Bicyclo[3.1.1]hept-2-ene-2-methanol, 6,6-dimethyl- | Terpenoids | woody, minty |
| 266 | Bicyclo[3.1.1]hept-3-en-2-one, 4,6,6-trimethyl-, (1S)- | Terpenoids | spicy, minty, camphor |
| 267 | Bicyclo[3.1.1]heptan-3-ol, 6,6-dimethyl-2-methylene- | Terpenoids | herbal, camphor, woody, pine, balsamic |
| 268 | Bicyclo[3.1.1]heptan-3-ol, 6,6-dimethyl-2-methylene-, [1S-(1.alpha.,3.alpha.,5.alpha.)]- | Terpenoids | warm, woody, balsamic, fennel |
| 269 | Bicyclo[3.1.1]heptane, 6,6-dimethyl-2-methylene-, (1S)- | Terpenoids | dry, woody, fresh, pine, hay, green, resinous |
| 270 | Biphenyl | Aromatics | pungent, rose, green, geranium |
| 271 | Bornyl acetate | Terpenoids | woody, pine, herbal, cedary, spice |
| 272 | Butanoic Acid, 2-methyl- | Acid | pungent, acid, roquefort, cheese |
| 273 | Butanoic Acid, 3-hydroxy-3-methyl- | Acid | amber, woody, cedarwood, ambergris |
| 274 | Butanoic Acid, 3-methylbutyl ester | Ester | fruity, green, apricot, pear, banana |
| 275 | Butanoic acid, 1-phenylethyl ester | Ester | sweet, fruity, berry, juicy, earthy, floral, jasmin |
| 276 | Butanoic acid, 2-methyl-, 2-methylpropyl ester | Ester | sweet, fruity |
| 277 | Butanoic acid, 2-methyl-, hexyl ester | Ester | green, waxy, fruity, apple, spicy, tropical |
| 278 | Butanoic acid, 2-methyl-, propyl ester | Ester | winey, fruity, apple, pineapple |
| 279 | Butanoic acid, 3-hexenyl ester, (Z)- | Ester | fresh, green, apple, fruity, wine, metallic, buttery |
| 280 | Butanoic acid, 3-methyl-, 2-methylbutyl ester | Ester | herbal, fruity, earthy, cheese, apple, green |
| 281 | Butanoic acid, 3-methyl-, hexyl ester | Ester | sweet, green fruit, apple, unripe apple skin, strawberry |
| 282 | Butanoic acid, anhydride | Others | buttery |
| 283 | Butanoic acid, butyl ester | Ester | fruity, banana, pineapple, green, cherry, tropical fruit, ripe fruit, juicy fruity |
| 284 | Butanoic acid, hexyl ester | Ester | green, sweet, fruity, apple, waxy, soapy |
| 285 | Butanoic acid, octyl ester | Ester | fresh, waxy, fruity, green, oily, earthy, creamy, natural, jasmin |
| 286 | Camphene | Terpenoids | woody, herbal, fir, needle, camphor, terpenic |
| 287 | Camphor | Terpenoids | camphor |
| 288 | Caprolactam | Amine | amine, spicy |
| 289 | Carvenone | Terpenoids | spearmint |
| 290 | Carveol | Terpenoids | minty, spearmint, cool, green, herbal, caraway, spicy |
| 291 | Carvone | Terpenoids | minty, licorice |
| 292 | Citral | Terpenoids | sharp, lemon, sweet |
| 293 | Citronellal | Terpenoids | sweet, dry, floral, herbal, waxy, aldehydic, citrus |
| 294 | Citronellyl isobutyrate | Ester | sweet, fruity, floral, geranium, tropical |
| 295 | Coumarin | Heterocyclic compound | sweet, hay, tonka, new-mown hay |
| 296 | Cubenene | Terpenoids | spicy, fruity, mango |
| 297 | Cyclohexanecarboxylic Acid | Acid | fruity, acid, metallic, cheese, tropical |
| 298 | Cyclohexanecarboxylic acid, ethyl ester | Ester | fruity, cheese, winey |
| 299 | Cyclohexanepropanoic Acid, 2-propenyl ester | Ester | sweet, pineapple, tropical, fruity, candy, waxy |
| 300 | Cyclohexanol, 1-methyl-4-(1-methylethylidene)- | Terpenoids | terpineol, lilac |
| 301 | Cyclohexanone, 5-methyl-2-(1-methylethyl)- | Terpenoids | minty |
| 302 | Cyclohexanone, 5-methyl-2-(1-methylethylidene)- | Terpenoids | minty |
| 303 | Cyclohexene, 1-methoxy- | Aromatics | herbal, spice |
| 304 | Cyclohexene, 1-methyl-4-(1-methylethylidene)- | Terpenoids | citrus, pine |
| 305 | Cyclopentanone, 2-(2-hexenyl)- | Ketone | oily, jasmin, coconut, milky, dairy, floral |
| 306 | Cyclopentanone, 2-(3-methyl-2-buten-1-yl)- | Ketone | light floral, jasmin |
| 307 | D-Limonene | Terpenoids | citrus |
| 308 | Decanal | Aldehyde | sweet, aldehydic, waxy, orange peel, citrus, floral |
| 309 | Decanoic acid, methyl ester | Ester | oily, wine, fruity, floral |
| 310 | DiSulfur compounds, dipropyl | Sulfur compounds | sulfury, earthy, burnt, green, onion |
| 311 | Diallyl disulphide | Sulfur compounds | alliaceous, onion, garlic, metallic |
| 312 | Diethyl Phthalate | Ester | bitter |
| 313 | Diethyl diSulfur compounds | Sulfur compounds | gassy, ripe onion, greasy, garlic |
| 314 | Dihydro-3-(2H)-thiophenone | Heterocyclic compound | garlic, meaty, green, vegetable, clam, buttery |
| 315 | Dihydrocarvyl acetate | Terpenoids | floral, rose, cumin, sweet, minty |
| 316 | Dimethyl Sulfoxide | Sulfur compounds | fatty, oily, cheese, garlic, mushroom |
| 317 | Dimethyl sulfone | Sulfur compounds | sulfury, burnt |
| 318 | Dimethyl triSulfur compounds | Sulfur compounds | sulfury, cooked onion, savory, meaty |
| 319 | Dodecanal | Aldehyde | soapy, waxy, aldehydic, citrus, green, floral |
| 320 | Dodecane | Hydrocarbons | alkane |
| 321 | Ethanethioic acid, S-(2-furanylmethyl) ester | Heterocyclic compound | sulfury, burnt, roasted, nutty, coffee |
| 322 | Ethanone, 1-(1,4-dimethyl-3-cyclohexen-1-yl)- | Terpenoids | fruity |
| 323 | Ethanone, 1-(1H-pyrrol-2-yl)- | Heterocyclic compound | musty, nut skin, maraschino, cherry, coumarin, licorice, walnut, bread |
| 324 | Ethanone, 1-(2-aminophenyl)- | Ketone | grape, sweet |
| 325 | Ethanone, 1-(2-furanyl)- | Heterocyclic compound | nutty, sweet, roasted |
| 326 | Ethanone, 1-(2-hydroxy-5-methylphenyl)- | Ketone | sweet, heavy, floral, herbal |
| 327 | Ethanone, 1-(2-methylphenyl)- | Ketone | sweet, hawthorn, powdery, anisic, coumarin, phenol, burnt, nutty, honey |
| 328 | Ethanone, 1-(2-pyridinyl)- | Heterocyclic compound | popcorn, heavy, corn, chip, fatty, tobacco |
| 329 | Ethanone, 1-(3,5-dimethylpyrazinyl)- | Heterocyclic compound | nutty, roasted, hazelnut |
| 330 | Ethanone, 1-(4-ethylphenyl)- | Ketone | floral, hawthorn |
| 331 | Ethyl (methylthio)acetate | Ester | sulfury, green, fruity, tropical |
| 332 | Ethyl 2-hexenoate, trans- | Ester | green, fruity, tropical, juicy, papaya, quince, winey, rummy, orange, vegetable |
| 333 | Eugenol | Phenol | floral, clove |
| 334 | Fenchol | Terpenoids | camphor, borneol, pine, woody, dry, sweet, lemon |
| 335 | Formic acid, octyl ester | Ester | fruity, rose, orange, waxy, cucumber |
| 336 | Formic acid, phenylmethyl ester | Ester | floral, fruity, spicy, almond, cranberry, black currant, tea |
| 337 | Furan, 2-ethyl-5-methyl- | Heterocyclic compound | fresh, gassy, burnt |
| 338 | Geranic acid | Terpenoids | green |
| 339 | Geranyl acetate | Terpenoids | lemon |
| 340 | Geranyl formate | Ester | fresh, rose, neroli, tea, rose, green |
| 341 | Germacrene D | Terpenoids | woody, spice |
| 342 | Glycerin | Alcohol | sweet |
| 343 | Heptane, 1,1-dimethoxy- | Hydrocarbons | green, privet, fruity, tropical, orange |
| 344 | Hexadecane | Hydrocarbons | alkane |
| 345 | Hexanal | Aldehyde | aldehyde, grassy, green, leafy, vinegar |
| 346 | Hexanoic acid, 2-methylbutyl ester | Ester | ethereal |
| 347 | Hexanoic acid, 2-methylpropyl ester | Ester | fruity, pineapple, green, apple skin, sour, tropical, peach, earthy |
| 348 | Hexanoic acid, 3-hexenyl ester, (Z)- | Ester | fruity, green, waxy, pear, winey, tropical, grassy, pineapple |
| 349 | Hexanoic acid, 3-hydroxy-, ethyl ester | Ester | fruity, grape, burnt, woody, hay, spicy, pineapple, cranberry, dusty, woody |
| 350 | Hexanoic acid, 3-oxo-, ethyl ester | Ester | fruity, pineapple, green, sweet, licorice, vanilla |
| 351 | Hexanoic acid, ethyl ester | Ester | apple, pear, fruity |
| 352 | Hexanoic acid, hexyl ester | Ester | herbal, fresh, grassy, vegetable, fruity |
| 353 | Hinesol | Terpenoids | spicy, peppery, woody |
| 354 | Hydrocinnamic Acid | Acid | sweet, fatty, rose, musky, cinnamon |
| 355 | Hydrocoumarin | Heterocyclic compound | sweet, tonka, coumarin, coconut, herbal, cinnamon |
| 356 | Indole | Heterocyclic compound | animalic, floral, moth, mothball, fecal, naphthelene |
| 357 | Indole, 3-methyl- | Heterocyclic compound | animalic, fecal, indole, civet |
| 358 | Ionone | Terpenoids | violet, sweet, floral, woody |
| 359 | Isoborneol | Terpenoids | balsamic, camphor, herbal, woody |
| 360 | Isobornyl acetate | Ester | balsamic, camphor, herbal, woody, sweet |
| 361 | Isobutyl isovalerate | Ester | sweet, fruity, apple, raspberry, green, banana |
| 362 | Isophorone | Ketone | cool, woody, sweet, green, camphor, fruity, musty, cedarwood, tobacco, leathery |
| 363 | Isoquinoline | Heterocyclic compound | sweet, balsamic, herbal, benzaldehyde, anisic |
| 364 | L-.alpha.-Terpineol | Terpenoids | lilac, floral, terpenic |
| 365 | Lilac Aldehyde C | Aldehyde | sweet, flowery |
| 366 | Lilac Aldehyde D | Aldehyde | sweet, flowery |
| 367 | Linalool | Terpenoids | floral, green |
| 368 | Linalyl acetate | Terpenoids | sweet, green, citrus, bergamot, lavender, woody |
| 369 | Maltol | Heterocyclic compound | sweet, caramel |
| 370 | Methyl ethyl diSulfur compounds | Sulfur compounds | sulfury, truffle |
| 371 | Methyl methacrylate | Ester | acrylate, aromatic, fruity |
| 372 | Methyl p-tolyloxyacetate | Ester | wild, fruity, fatty |
| 373 | Methyl salicylate | Ester | caramel, pepperminty |
| 374 | Naphthalene | Aromatics | pungent, dry, tarry |
| 375 | Naphthalene, 1,2,3,4-tetrahydro-1,6-dimethyl-4-(1-methylethyl)-, (1S-cis)- | Terpenoids | herbal, spice |
| 376 | Naphthalene, 1,2,3,5,6,8a-hexahydro-4,7-dimethyl-1-(1-methylethyl)-, (1S-cis)- | Terpenoids | thyme, herbal, woody, dry |
| 377 | Naphthalene, 1,2,4a,5,6,8a-hexahydro-4,7-dimethyl-1-(1-methylethyl)-, [1S-(1.alpha.,4a.beta.,8a.alpha.)]- | Terpenoids | woody, dry |
| 378 | Naphthalene, 1,2,4a,5,8,8a-hexahydro-4,7-dimethyl-1-(1-methylethyl)-, [1S-(1.alpha.,4a.beta.,8a.alpha.)]- | Terpenoids | green, woody |
| 379 | Naphthalene, 1,2-dihydro-1,1,6-trimethyl- | Aromatics | licorice |
| 380 | Naphthalene, 2,3,6-trimethyl- | Aromatics | fruity, dry |
| 381 | Naphthalene, 2,6-dimethyl- | Aromatics | grassy |
| 382 | Naphthalene, decahydro-4a-methyl-1-methylene-7-(1-methylethenyl)-, [4aR-(4a.alpha.,7.alpha.,8a.beta.)]- | Terpenoids | herbal |
| 383 | Nonane | Hydrocarbons | linseed, oily, oily, sweaty |
| 384 | Nonanoic Acid | Acid | waxy, dirty, cheese, cultured, dairy |
| 385 | Nonanoic acid, methyl ester | Ester | sweet, fruity, pear, waxy, tropical, wine |
| 386 | Octanal | Aldehyde | lemon, citrus, green grass |
| 387 | Octane | Hydrocarbons | gasoline |
| 388 | Octanoic acid, ethyl ester | Ester | fruity, banana |
| 389 | Pantolactone | Ester | burnt, roasted bread |
| 390 | Paroxypropione | Ketone | phenol |
| 391 | Pentadecane | Hydrocarbons | waxy |
| 392 | Pentanoic acid, 2-hydroxy-3-methyl-, methyl ester | Ester | fruity, estery, caramel |
| 393 | Pentanoic acid, 2-hydroxy-4-methyl-, methyl ester | Ester | sweet, fruity, musty |
| 394 | Pentanoic acid, 2-methyl-, ethyl ester | Ester | fresh fruit, green, melon, apple skin, pineapple, natural, waxy |
| 395 | Pentanoic acid, 4-methyl-, ethyl ester | Ester | fruity |
| 396 | Phenol | Phenol | phenol, medicinal |
| 397 | Phenol, 2-ethyl- | Phenol | phenol |
| 398 | Phenol, 2-methoxy-4-propyl- | Aromatics | clove, sharp, spicy, sweet, phenol, powdery, allspice |
| 399 | Phenol, 2-methyl- | Phenol | phenol |
| 400 | Phenol, 2-methyl-5-(1-methylethyl)- | Terpenoids | spice, woody, camphor, thymol |
| 401 | Phenol, 3,5-dimethyl- | Phenol | balsamic, coffee |
| 402 | Phenol, 3-ethyl- | Phenol | musty |
| 403 | Phenol, 4-ethyl-2-methoxy- | Phenol | clove, candy |
| 404 | Phenol, p-tert-butyl- | Phenol | oakmoss, leathery |
| 405 | Phenylacetic acid propyl ester | Ester | sweet, honey, floral, apricot, rose |
| 406 | Pinocarvone | Terpenoids | minty |
| 407 | Piperitenone oxide | Ketone | herbal, minty |
| 408 | Propanoic Acid, 2-oxo- | Acid | sharp, sour, acetic, caramel |
| 409 | Propanoic acid, 2-methyl-, 2-methylbutyl ester | Ester | fruity, ethereal, tropical, banana |
| 410 | Propanoic acid, 2-methyl-, 2-phenylethyl ester | Ester | floral, fruity, rose, tea, rose, peach, pastry |
| 411 | Propanoic acid, 2-methyl-, 3-phenylpropyl ester | Ester | fruity, tropical, apricot, plum, balsamic, jammy, honey, bread, osmanthus, dry |
| 412 | Propanoic acid, 3-(methylthio)- | Sulfur compounds | sweet, sulfury |
| 413 | Propanoic acid, butyl ester | Ester | earthy, sweet, weak, rose |
| 414 | Pyrazine, 2,3-dimethyl- | Heterocyclic compound | nutty, nut skin, cocoa, peanut, buttery, coffee, walnut, caramel, roasted |
| 415 | Pyrazine, 2,3-dimethyl-5-(1-methylpropyl)- | Heterocyclic compound | marine, burnt, roasted |
| 416 | Pyrazine, 2,5-dimethyl- | Heterocyclic compound | cocoa, roasted, nutty, roasted, beefy, woody, grassy, medicinal |
| 417 | Pyrazine, 2,6-dimethyl- | Heterocyclic compound | ethereal, cocoa, nutty, roasted, roasted, meaty, beefy, brown, coffee, buttermilky |
| 418 | Pyrazine, 2-ethyl-3,5-dimethyl- | Heterocyclic compound | burnt, almond, roasted, nutty, coffee |
| 419 | Pyrazine, 2-ethyl-3-methyl- | Heterocyclic compound | nutty, peanut, musty, corn, raw, earthy, bread |
| 420 | Pyrazine, 2-ethyl-5-methyl- | Heterocyclic compound | coffee, beany, nutty, grassy, roasted |
| 421 | Pyrazine, 2-methoxy-3-(1-methylethyl)- | Heterocyclic compound | beany, pea, earthy, chocolate, nutty |
| 422 | Pyrazine, 2-methoxy-3-(1-methylpropyl)- | Heterocyclic compound | musty, green, pea, galbanum, bell pepper, pepper |
| 423 | Pyrazine, 2-methoxy-3-methyl- | Heterocyclic compound | roasted almond, hazelnut, peanut |
| 424 | Pyrazine, 2-methoxy-6-methyl- | Heterocyclic compound | roasted hazelnut, almond, peanut |
| 425 | Pyrazine, 2-methyl-3-(methylthio)- | Heterocyclic compound | roasted meat, nutty, almond, vegetable |
| 426 | Pyrazine, 2-methyl-5-(1-methylethyl)- | Heterocyclic compound | green, coffee, nutty, earthy |
| 427 | Pyrazine, 3,5-diethyl-2-methyl- | Heterocyclic compound | nutty, meaty, vegetable |
| 428 | Pyrazine, 3-ethyl-2,5-dimethyl- | Heterocyclic compound | potato, cocoa, roasted, nutty |
| 429 | Pyrazine, methoxy- | Heterocyclic compound | sweet, nutty, cocoa |
| 430 | Pyrazine, methyl- | Heterocyclic compound | nutty, cocoa, roasted, chocolate, peanut, green |
| 431 | Pyrazine, tetramethyl- | Heterocyclic compound | nutty, musty, chocolate, coffee, cocoa, brown, lard, burnt |
| 432 | Pyrazine, trimethyl- | Heterocyclic compound | nut skin, earthy, powdery, cocoa, baked, potato, roasted, peanut, hazelnut, musty |
| 433 | Pyridine, 2-ethyl- | Heterocyclic compound | green, grassy |
| 434 | Pyridine, 2-methyl- | Heterocyclic compound | sweaty |
| 435 | Pyridine, 2-pentyl- | Heterocyclic compound | fatty, tallow, green, pepper, mushroom, herbal |
| 436 | Quinoline | Heterocyclic compound | musty, tobacco, rubbery, earthy |
| 437 | Quinoxaline, 2-methyl- | Heterocyclic compound | toasted, coffee, nutty, fruity |
| 438 | Styrene | Aromatics | penetrating, balsamic, gasoline |
| 439 | TRANS-ANETHOLE | Aromatics | sweet, anisic, licorice, mimosa |
| 440 | Terpinen-4-ol | Terpenoids | turpentine, nutmeg, musty |
| 441 | Tetradecane | Hydrocarbons | mild, waxy |
| 442 | Thiazole, 2,4,5-trimethyl- | Heterocyclic compound | musty, nutty, vegetable, cocoa, hazelnut, chocolate, coffee |
| 443 | Thiazole, 2,4-dimethyl- | Heterocyclic compound | coffee, tea, beefy, roasted, barley |
| 444 | Thiazole, 4,5-dimethyl- | Heterocyclic compound | roasted, nutty, fishy, green, boiled, shrimp |
| 445 | Thiophene, 2-butyl-5-ethyl- | Heterocyclic compound | fruity, berry, earthy |
| 446 | Thiophene, 2-methyl- | Heterocyclic compound | sulfury, alliaceous, onion, roasted, green |
| 447 | Thiophene, 2-pentyl- | Heterocyclic compound | fatty, chicken, cranberry, roasted hazelnut, corn, roasted barley, fermented, meaty, blood |
| 448 | Thiophene, 3-methyl- | Heterocyclic compound | fatty, winey |
| 449 | Thujone | Terpenoids | cedar leaf |
| 450 | Thymol | Terpenoids | herbal, thyme, phenol, medicinal, camphor |
| 451 | TriSulfur compounds, dipropyl | Sulfur compounds | sulfury, green, onion, garlic, tropical |
| 452 | TriSulfur compounds, methyl 2-propenyl | Sulfur compounds | alliaceous, creamy, garlic, onion |
| 453 | Undecanoic acid, methyl ester | Ester | fatty, waxy, fruity |
| 454 | Vanillin | Aldehyde | sweet, vanilla, creamy, chocolate |
| 455 | Verbenone | Terpenoids | camphor, menthol, celery |
| 456 | [1aR-(1a.alpha.,4.alpha.,4a.beta.,7b.alpha.)]-1a,2,3,4,4a,5,6,7b-octahydro-1,1,4,7-tetramethyl-1H-Cycloprop[e]azulene | Terpenoids | woody, balsamic |
| 457 | cis-3-Hexenyl cis-3-hexenoate | Ester | green, tomato, leafy, pear, melon, metallic, fennel, tropical |
| 458 | cis-Dihydrocarvone | Terpenoids | herbal, warm |
| 459 | n-Decanoic Acid | Acid | fatty, rancid, soapy, unpleasant, rancid, sour, fatty, citrus |
| 460 | n-Heptyl hexanoate | Ester | damp, green, sappy, fresh, bruised, mallow, dock, leafy |
| 461 | n-Hexylacetoacetic acid ethyl ester | Ester | fruity, jasmin, waxy, herbal |
| 462 | o-Xylene | Aromatics | geranium |
| 463 | p-Cresol | Phenol | phenol, narcissus, animalic, mimosa |
| 464 | p-Cymen-7-ol | Alcohol | mild, amber, balsamic, fruity |
| 465 | p-Menth-8-en-3-ol, acetate | Ester | minty, leafy |
| 466 | p-Mentha-1,8-dien-7-ol | Terpenoids | green, cumin, spicy, aromatic, woody, cardamom, floral, waxy, violet |
| 467 | p-Menthane-3,8-diol, cis-1,3,trans-1,4- | Terpenoids | herbal, eucalyptus, minty |
| 468 | p-Xylene | Aromatics | strong, sweetish |
| 469 | trans,cis-2,6-Nonadien-1-ol | Alcohol | green, cucumber, oily, violet, leafy |
| 470 | trans-.alpha.-Bergamotene | Terpenoids | woody, warm, tea |
| 471 | trans-.beta.-Ocimene | Terpenoids | sweet, herbal |
| 472 | trans-3-Methyl-4-octanolide | Ester | spicy, coconut, clove, celery, incense |
| 473 | trans-4-MethoxycinnamAldehyde | Aldehyde | spicy, floral, sweet, cinnamon, cherry, vanilla |
| 474 | trans-Geranic acid methyl ester | Terpenoids | waxy, green, fruity, flowery |
| 475 | trans-Isoeugenol | Phenol | floral, clove |
